# Two Total Syntheses of Trigoxyphins K and L

**DOI:** 10.1021/acs.orglett.3c02796

**Published:** 2023-10-06

**Authors:** Shuyang Li, Jack A. O’Hanlon, Andrew Mattimoe, Helena D. Pickford, Lucy A. Harwood, Luet L. Wong, Jeremy Robertson

**Affiliations:** † Department of Chemistry, University of Oxford, Chemistry Research Laboratory, Mansfield Road, Oxford OX1 3TA, United Kingdom; ‡ Department of Chemistry, University of Oxford, Inorganic Chemistry Laboratory, South Parks Road, Oxford OX1 3QR, United Kingdom; § Oxford Suzhou Centre for Advanced Research, Ruo Shui Road, Suzhou Industrial Park, Suzhou, Jiangsu 215123, People’s Republic of China

## Abstract

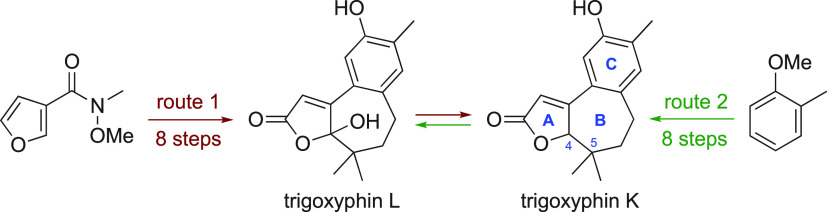

Two total syntheses are presented for trigoxyphins K and L, tricyclic terpenoids from *Trigonostemon xyphophylloides*. The first proceeds via electrophlic cyclization in A/C-ring substrates to close the B ring at C4–C5 and then ^1^O_2_-mediated hydroxybutenolide formation to trigoxyphin L, with Luche reduction leading to trigoxyphin K. The second route develops from tetralone ring expansion to a B/C-ring intermediate that, by one-step O-demethylation–lactonization–isomerization, affords trigoxyphin K and then trigoxyphin L following enolate oxygenation.

The “degraded diterpenes” trigoxyphins K and L (Figure 1)^1^ are two of many secondary metabolites of terpenoid origin to be isolated from *Trigonostemon xyphophylloides*, a flowering plant of the Euphorbiaeceae family. The structures were established by extensive nuclear magnetic resonance (NMR) spectroscopic analysis; although both are chiral, no specific rotation data are reported, and therefore, it is not known whether trigoxyphin K, at least, is obtained as a single enantiomer (trigoxyphin L would likely racemize rapidly in solution). As a result of near simultaneous reports from different researchers of further metabolites from the same plant, the names trigoxyphins J and K were attributed to two daphnane diterpenoids^2^ unrelated in structure to trigoxyphins J and K reported by Wu and Han. These trigoxyphins have been found in other plants of the Euphorbiaceae family; for example, trigoxyphin K was isolated from the stem bark of *Sagotia racemosa*,^3^ and trigoxyphin L was isolated from the roots and leaves of *Strophioblachia glandulosa*,^4^ although in the publication the structure depicted was incorrectly attributed to trigoxyphin K. Many of the metabolites are appreciably toxic against human cancer cell lines, and beneficial cardiovascular effects have been attributed to trigoxyphin K in a series of patents.^5^

**Figure 1:**
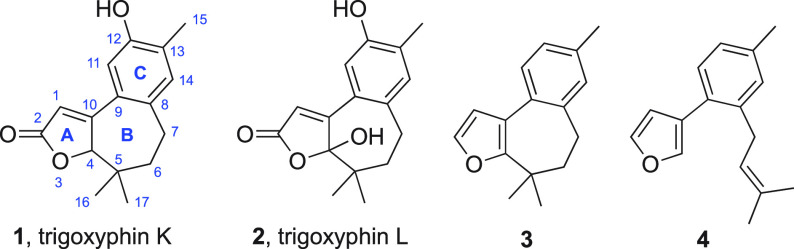
Trigoxyphins K and L and potential precursor **3** via compound **4**.

Arising from our research on harnessing engineered cytochrome P450_BM3_ variants for a variety of synthetic applications, we considered the relatively simple structures of trigoxyphins K and L to provide an arena for evaluating a double enzymatic oxidation of precursors, such as tricyclic furan **3**. Here, the furan would be oxidized^6,7^ to the (hydroxy)butenolide and the benzene ring would be hydroxylated^8^ with reactivity and selectivity tuned by choice of the P450_BM3_ variant. This paper reports the synthesis of tricycle **3**, planned to be obtained by Brønsted-acid-initiated cyclization of biaryl **4**, and two separate chemical total syntheses of trigoxyphins K and L. The metabolites generated by enzymatic oxidation of compound **3** and related compounds will be described elsewhere.

A short synthesis of oxidation precursor **3** was envisaged, in which the cycloheptane (B) ring would be obtained by connection of the C4–C5 bond by acidic activation of the prenyl substituent in biaryl derivative **4**, which, in turn, was planned to be prepared by prenylation of Suzuki coupling^9^ product **5** (Scheme 1). Lithium–halogen exchange in compound **5** and alkylation with prenyl bromide proved unsatisfactory because the organometallic compound had marginal stability at a temperature much above that of its generation; however, complete alkylation with the more reactive electrophile prenal was achieved at −78 °C to give alcohol **6**. Attempts to employ this alcohol as a cationic cyclization precursor resulted in either simple elimination or intractable product mixtures. Instead, Lewis acid activation^10^ of epoxide **7**, formed from compound **6** as a single diastereomer,^11^ afforded tricyclic product **8** (from which the relative configuration in epoxide **7** was confirmed retrospectively). Pinacol-type rearrangement^12^ (→ **9**) and Wolff–Kishner reduction completed the synthesis, in six steps overall. Neither enzymatic nor chemical installation of the 12-OH substituent could be achieved from tricycle **3**, but furan oxidation under Faulkner’s conditions^13^ afforded 12-deoxytrigoxyphin L **10**, which gave the trigoxyphin K analogue **11** upon Luche reduction.^14^

**Scheme 1:**
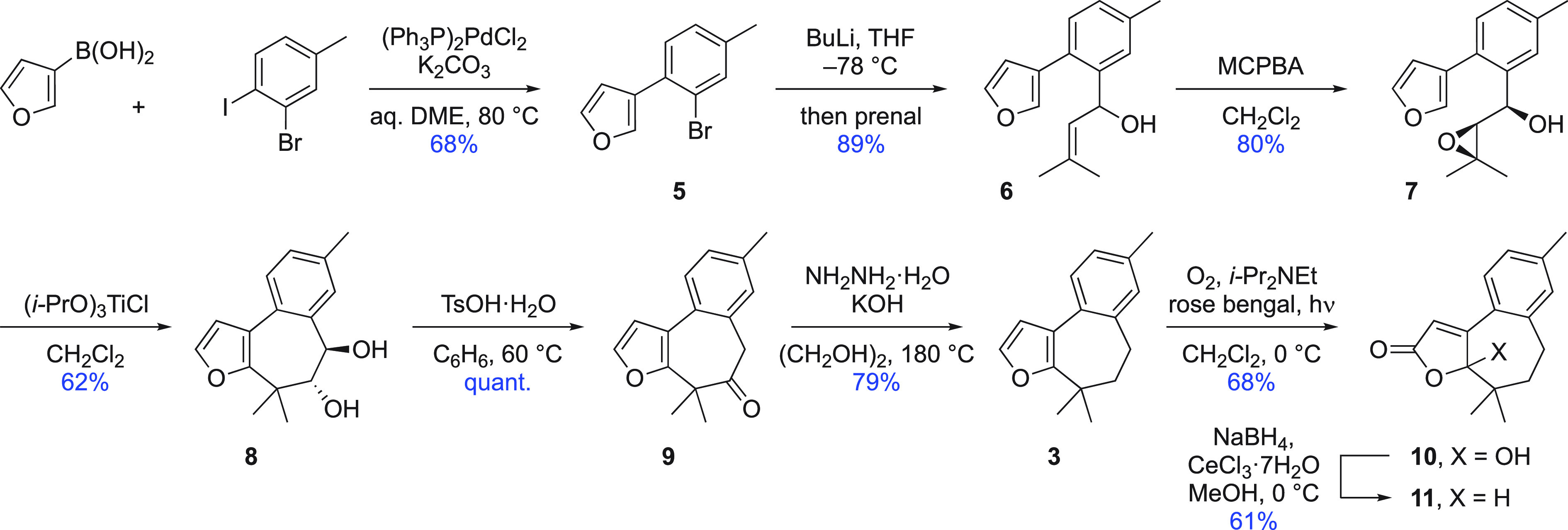
Synthesis of 12-Deoxytrigoxyphins K and L

Adapting this route to incorporate the C12 phenol at the outset was expected to excessively frontload the synthesis with extra steps needed to access the appropriate benzenoid partner for the Suzuki coupling. Accordingly, the next iteration sought to establish the C ring by Robinson annulation, in which the prenyl side chain would already be present. This new sequence began with Grignard addition to Weinreb amide **12**(15) (Scheme 2) and then silylation of the so-formed ketone **13** in readiness for 1,4-addition to methyl vinyl ketone (MVK). This step (→ **14**) was most effectively achieved by a modification of Loh’s method with indium(III) chloride.^16^ For this application, to avoid extensive tarring of the furan derivative, the reaction was moderated by including a solvent and keeping the catalyst loading to 2 mol % (cf. reported conditions: neat and 20 mol % catalyst, respectively). Intramolecular aldol condensation under classical conditions gave cyclohexenone **15**, which was efficiently methylenated^17^ (→ **16**) and aromatized under basic conditions^18^ to generate phenol derivative **17**. The crucial acid-catalyzed cyclization to close the B ring was expected to require carefully chosen conditions because of the natural tendency of furans to decompose in the presence of both protic and Lewis acids, which, here, would be exacerbated by attachment to a free phenol. The lack of simple alkenes as electrophiles in Tanis’ work^10^ and a 0% yield in a related cyclization^19^ gave further cause for concern. Noting the particular effectiveness of 1,1,1,3,3,3-hexafluoroisopropanol (HFIP)^20^ as an additive in promoting camphor sulfonic acid (CSA)-catalyzed tandem cyclizations, Spivey’s reported conditions^21^ were applied to intermediate **17**. In the event, cyclization progressed steadily to give tricyclic phenol **18**, with the only complication arising from competing deprenylation of the substrate. Trigoxyphins L and K were then obtained by the singlet oxidation and Luche reduction steps used previously.

**Scheme 2:**
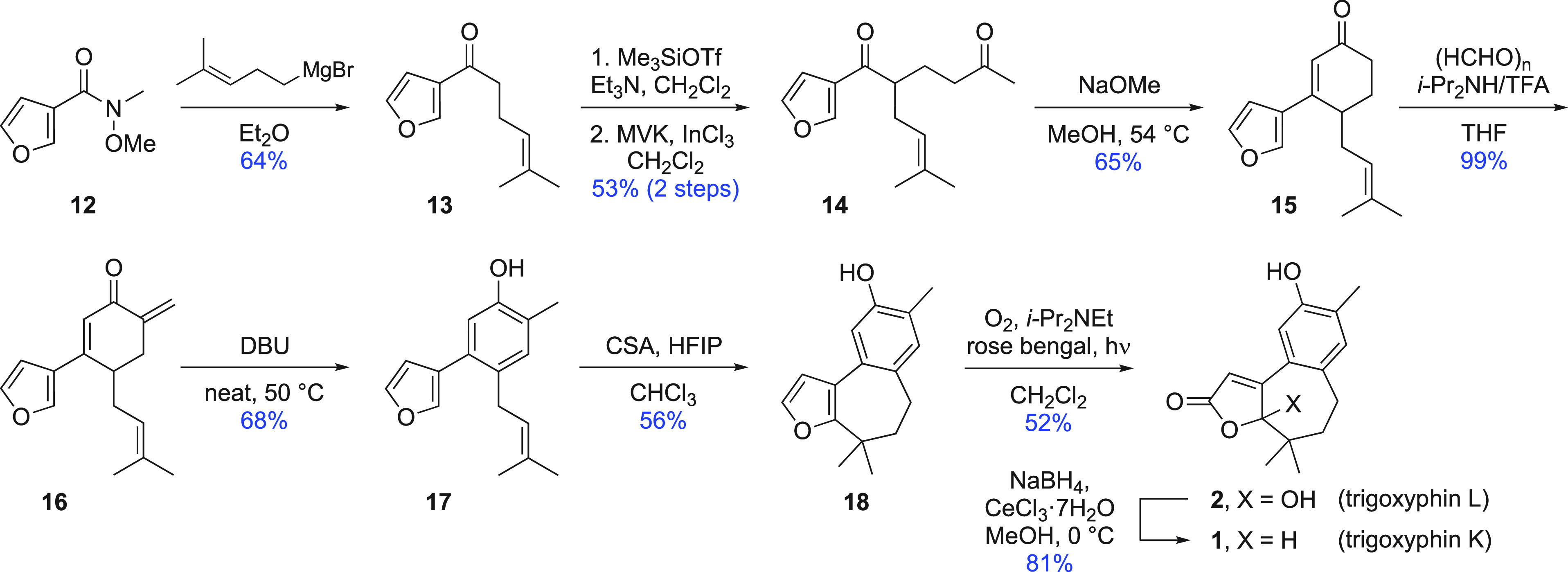
Initial Synthesis of Trigoxyphins K and L (Route 1)

Both sequences developed to this point started with relatively expensive 3-substituted furan derivatives: the boronic acid in the first route and the carboxylic acid in the second route. Routes originating with the more accessible 2-substituted furans are, however, complicated by the *gem*-dimethyl functionality, and therefore, an alternative strategy was considered. In this third approach, the A ring would be formed as the final step from an appropriately functionalized 2-benzosuberone that, in turn, would be obtained from the ring expansion of known tetralone derivative **19** (Scheme 3). This ketone, obtained in three steps (∼50% yield) from 2-methylanisole,^22^ was dimethylated^23^ (→ **20**) and converted into the corresponding alkene **21** by Wittig methylenation under standard conditions. In model studies of the ring expansion of 1-methylene-1,2,3,4-tetrahydronaphthalene and its 2,2-dimethyl derivative, Silva’s modification^24^ of Koser’s method with [hydroxy(tosyloxy)iodo]benzene (HTIB)^25^ was found to work well. With alkene **21**, however, isolated yields were much reduced because of the ease of oxidation of the methylene group flanked by carbonyl functionality and an electron-rich aromatic system in the product. Efficient reaction was restored by replacing Silva’s combination of iodobenzene and *meta*-chloroperbenzoic acid (*m*CPBA) with a slight excess of (diacetoxy)iodobenzene, affording cycloheptanone derivative **22**. The reaction conditions for a one-step method^26^ using glyoxylic acid to introduce the hydroxybutenolide functionality required for trigoxyphin L worked well in the above-mentioned model study; however, this process proved too harsh for ketone **22**, and complex reaction mixtures resulted. Eventually, the most direct solution was found in enolate alkylation and then treatment of the so-formed keto ester **23** with boron tribromide. This latter reagent not only removed the phenolic *O*-methyl substituent as expected^27^ but also promoted lactonization and alkene isomerization^28^ to deliver trigoxyphin K directly.^29^ In a reversal of the final end-game steps in the previous two routes, trigoxyphin K was converted into trigoxyphin L by oxygenation of the extended enolate formed under reversible conditions.^30^

**Scheme 3:**
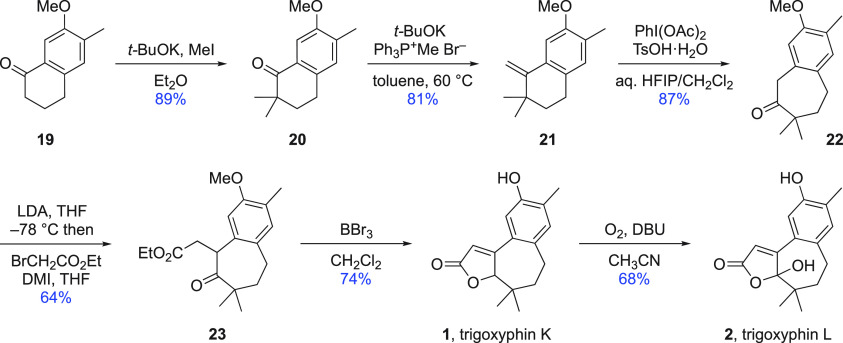
Improved Synthesis of Trigoxyphins K and L (Route 2)

This project was predicated on the general idea that late-stage oxidation of (mainly) hydrocarbon precursors could deconstrain the analysis of target synthesis problems. Had our initial efforts (Scheme 1) led to a direct incorporation of the bare prenyl side chain rather than the benzylic alcohol (in compound **6**), access to target **3** would have been achieved in just three steps. Complications arising from this unwanted hydroxyl substituent necessitated raising the oxidation level (to the epoxide **7**) to enable clean cyclization of the B ring; in turn, this meant that two further steps were necessary to remove the diol functionality. Conceptually, then, this project taught that the advantage that a late-stage oxidation approach may bring to synthesis can be undone by redox inefficiencies in accessing low-oxidation-state substrates.

In the first complete route (Scheme 2), direct methylenation and isomerization of the Robinson annulation product **15** streamlined access to the B-ring cyclization precursor, leading to a highlight of this route in the CSA/HFIP-mediated cyclization, the first case of such a furan-terminated 7-*endo*-*trig* cyclization onto a simple (unconjugated) alkene. Essentially all the steps in the sequence are strategic C–C bond-forming or redox processes, and the total syntheses are just one step longer than the route to the 12-deoxy analogues.

The second complete route (Scheme 3) dispensed with the previous “furan first” approach, which enabled a much more satisfying synthesis that would, in principle, be shortened further by an efficient direct ring expansion from **20** → **22**. Here, the finding that the conditions for de-O-methylation would also complete the butenolide formation led to a direct synthesis of trigoxyphin K and then trigoxyphin L in a logical order from a redox perspective. The overall route is short (8–9 steps), efficient (∼10% overall; >75% average per step), and both practical and scalable, appropriate for the production of analogues by variation of the initial benzocycloalkanone and the introduced alkyl substituents.

Further supporting synthetic studies in this project and the outcomes of enzymatic screening applied to compound **3** and related substrates will be reported in due course.

## Data Availability

The data underlying this study are available in the published article and its Supporting Information.

## Supporting Information Available

Experimental procedures, characterization data, and copies of ^1^H and ^13^C NMR spectra for all compounds (PDF)
