# Supplementary material for: Two Total Syntheses of Trigoxyphins K and L
Source: Org Lett. 2023 Oct 6;25(41):7507–11. doi: 10.1021/acs.orglett.3c02796 (PMC10594648; doi:10.1021/acs.orglett.3c02796)

Supporting Information

Two Total Syntheses of Trigoxyphins K and L

Shuyang Li,[1] Jack A. O'Hanlon,[1] Andrew Mattimoe,[1] Helena D. Pickford,[1] Lucy A. Harwood,[1] Luet L. Wong,[2,3] and Jeremy Robertson[1,3]*

[1]Department of Chemistry, University of Oxford, Chemistry Research Laboratory, Mansfield Road, Oxford, OX1 3TA (UK)
[2]Department of Chemistry, University of Oxford, Inorganic Chemistry Laboratory, South Parks Road, Oxford, OX1 3QR (UK)
[3]Oxford Suzhou Centre for Advanced Research, Ruo Shui Road, Suzhou Industrial Park, Jiangsu, 215123 (P. R. China)

Table of Contents

General methods S2

Experimental procedures and characterisation data [Scheme order] S3

NMR spectra for synthesised compounds [Numerical order] S15–S37

Trigoxyphin K (**1**) S15–S16

Trigoxyphin L (**2**) S17–S19

4,4,8-Trimethyl-5,6-dihydro-4*H*-benzo[3,4]cyclohepta[1,2-*b*]furan (**3**) S20

3-(2-Bromo-4-methylphenyl)furan (**5**) S21

1-[2-(Furan-3-yl)-5-methylphenyl]-3-methylbut-2-en-1-ol (**6**) S22

(*R**)-[(*S**)-3,3-Dimethyloxiran-2-yl][2-(furan-3-yl)-5-methylphenyl]methanol (**7**) S23

(5*R**,6*R**)-4,4,8-Trimethyl-5,6-dihydro-4*H*-benzo[3,4]cyclohepta[1,2-*b*]furan-5,6-diol (**8**) S24

4,4,8-Trimethyl-4,6-dihydro-5*H*-benzo[3,4]cyclohepta[1,2-*b*]furan-5-one (**9**) S25

3a-Hydroxy-4,4,8-trimethyl-3a,4,5,6-tetrahydro-2*H*-benzo[3,4]cyclohepta[1,2-*b*]furan-2-one (**10**) S26

4,4,8-Trimethyl-3a,4,5,6-tetrahydro-2*H*-benzo[3,4]cyclohepta[1,2-*b*]furan-2-one (**11**) S27

1-(Furan-3-yl)-5-methylhex-4-en-1-one (**13**) S28

1-(Furan-3-yl)-2-(3-methylbut-2-en-1-yl)hexane-1,5-dione (**14**) S29

3-(Furan-3-yl)-4-(3-methylbut-2-en-1-yl)cyclohex-2-en-1-one (**15**) S30

3-(Furan-3-yl)-4-(3-methylbut-2-en-1-yl)-6-methylenecyclohex-2-en-1-one (**16**) S31

5-(Furan-3-yl)-2-methyl-4-(3-methylbut-2-en-1-yl)phenol (**17**) S32

4,4,8-Trimethyl-5,6-dihydro-4*H*-benzo[3,4]cyclohepta[1,2-*b*]furan-9-ol (**18**) S33

7-Methoxy-2,2,6-trimethyl-3,4-dihydronaphthalen-1(2*H*)-one (**20**) S34

7-Methoxy-2,2,6-trimethyl-1-methylene-1,2,3,4-tetrahydronaphthalene (**21**) S35

3-Methoxy-2,7,7-trimethyl-5,7,8,9-tetrahydro-6*H*-benzo[7]annulen-6-one (**22**) S36

Ethyl 2-(3-methoxy-2,7,7-trimethyl-6-oxo-6,7,8,9-tetrahydro-5*H*-benzo[7]annulen-5-yl)acetate (**23**) S37

General methods

Procedures are presented in the order given in the Schemes except for the natural products which are described at the end of the section.

All solvents for anhydrous reactions were obtained dry from Grubbs solvent dispenser units after being passed through an activated alumina column under argon. THF was additionally distilled from sodium/benzophenone ketyl under argon. Commercially available reagents were, in general, used as supplied; amines and dipolar aprotic solvents were purified by standard methods before use. “Petrol” refers to the fraction of light petroleum ether boiling in the range of 30–40 °C; “ether” refers to diethyl ether. Unless stated otherwise, all reactions were carried out in oven-dried glassware and under an inert atmosphere (N_2 or Ar as specified); reactions performed above ambient temperature were heated using a thermostatically-controlled oil bath. Silica gel chromatography was carried out using Geduran Silicagel 60, particle size 40–63 µm. Thin-layer chromatography (TLC) was conducted after all reactions whenever practical, using Merck aluminium-backed Silicagel 60 F254 fluorescent treated silica; visualisation was enabled by UV light (λ_{max} = 254 nm) and staining with potassium permanganate or phosphomolybdic acid solution to give the retention factors (R_f) quoted. Compound names are as generated by PerkinElmer ChemDraw Professional 22.2. Melting points (mp) were recorded (uncorrected) in degrees Celsius (°C), using a Griffin MFB-700-010U melting point apparatus. IR spectra were recorded on a Bruker Tensor 27 FT-IR spectrometer as a thin film on a diamond ATR module. Only selected absorption maxima (ν_{max}) are reported, in wavenumbers (cm^{-1}). ^1H and ^{13}C NMR spectra were recorded using a Bruker AVIIIHD-400 spectrometer using the solvents specified. Chemical shifts are quoted in ppm downfield of tetramethylsilane (δ = 0) and referenced in MestReNova to the appropriate solvent peak: $CDCl_3$, 7.26/77.16; C_6D_6, 7.16/128.06; $(CD_3)_2CO$, 2.05/29.84, CD_3OD, 3.31/49.00. Coupling constants (J) are quoted in Hz, rounded to the nearest 0.5 Hz. All ^1H NMR spectra are reported as follows: ppm (number of protons, multiplicity, coupling constants). High-resolution mass spectra (HRMS) were recorded by the staff at the Chemistry Research Laboratory (University of Oxford) using a Bruker Daltonics MicroTOF spectrometer; mass-to-charge ratios (m/z) are reported in Daltons.

Experimental procedures and characterisation data

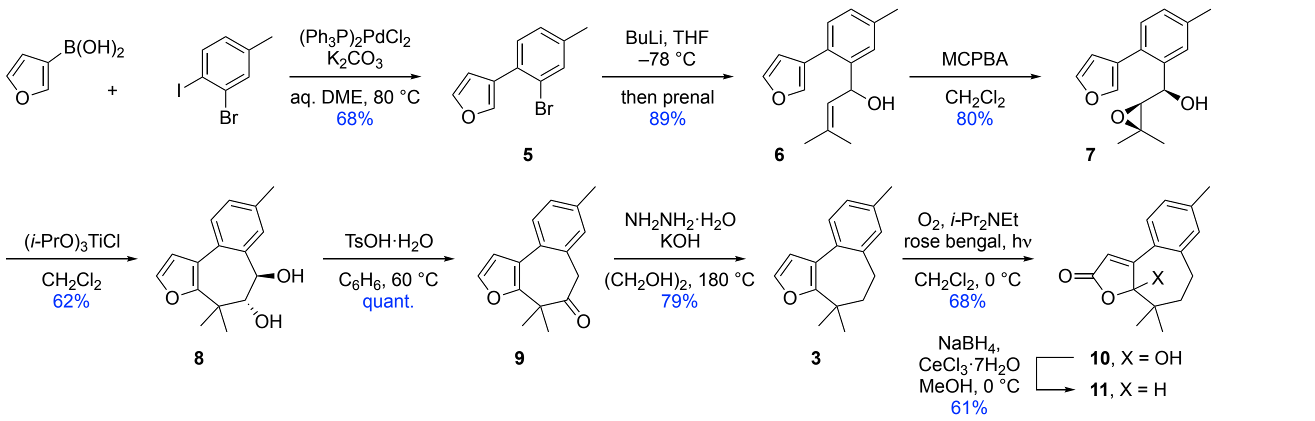

*3-(2-Bromo-4-methylphenyl)furan (**5**)*

Ar was bubbled through a solution of water (1.7 mL) and dimethoxyethane (11 mL) for 5 min. Furan-3-ylboronic acid (223 mg, 2.00 mmol), 2-bromo-1-iodo-4-methylbenzene (493 mg, 1.67 mmol), and K_2CO_3 (574 mg, 4.16 mmol) were added and Ar was bubbled through the solution for a further 5 min. $(Ph_3P)_2PdCl_2$ (58 mg, 0.083 mmol) was added and the solution was heated at 80 °C for 19 h then cooled to RT and water (10 mL) added. The solution was extracted with ether (3 × 20 mL) and the combined organic layers were washed with brine (15 mL), dried ($MgSO_4$), filtered, and concentrated. The crude product was purified by flash chromatography (petrol) to afford the title compound (**5**) as a colourless oil (268 mg, 68%). R_f 0.20 (petrol); IR (thin film) ν_{max}/cm^{-1} 2923m, 1511m, 1165m, 1064m, 1036m, 1015m, 874s, 824m, 789s; ^{1}H NMR (400 MHz, $CDCl_3$) δ_H 7.76 (1H, dd, *J* = 1.5, 1.0 Hz), 7.49 (1H, br s), 7.48 (1H, t, *J* = 1.5 Hz), 7.28 (1H, d, *J* = 8.0 Hz), 7.13 (1H, ddd, *J* = 8.0, 1.5, 0.5 Hz), 6.70 (1H, dd, *J* = 1.5, 1.0 Hz), 2.35 (3H, s); ^{13}C NMR (101 MHz, $CDCl_3$) δ_C 142.4, 140.8, 138.8, 134.1, 130.7, 130.6, 128.4, 125.1, 122.3, 111.6, 20.8; HRMS (CI+) *m/z* $[M+H]^+$ calcd for $C_{11}H_{10}{}^{79}BrO$, 236.9910; found, 236.9915.

*1-[2-(Furan-3-yl)-5-methylphenyl]-3-methylbut-2-en-1-ol (**6**)*

A solution of furan derivative **5** (500 mg, 2.11 mmol) in tetrahydrofuran (14 mL) was cooled to –78 °C and butyllithium (0.93 mL, 2.5 M in hexanes, 2.3 mmol) was added dropwise. The solution was stirred for 10 min then prenal (3,3-dimethylacrolein, 0.22 mL, 2.3 mmol) was added dropwise. The solution was stirred at –78 °C for 1 h then warmed to RT and quenched with saturated aqueous $NaHCO_3$ solution (10 mL). The solution was extracted with ethyl acetate (3 × 20 mL) and the combined organic layers were washed with brine (20 mL), dried (Na_2SO_4), filtered, and concentrated. The crude product was purified by flash chromatography (petrol/ether, 9:1 to 4:1) to afford the title compound (**6**) as a

colourless oil (455 mg, 89%). R_f 0.15 (petrol/ether, 4:1); IR (thin film) ν_{max}/cm^{-1} 3343br m, 2980w, 2916w, 1509w, 1448w, 1377w, 1160m, 1083m, 1014m, 873s, 823m, 790s; ^{1}H NMR (400 MHz, C_6D_6) δ_H 7.63 (1H, s), 7.36 (1H, dd, *J* = 1.5, 1.0 Hz), 7.14 (1H, t, *J* = 1.5 Hz) overlaying 7.13 (1H, d, *J* = 8.0 Hz), 6.93 (1H, br d, *J* = 8.0 Hz), 6.37 (1H, dd, *J* = 1.5, 1.0 Hz), 5.61 (1H, dd, *J* = 8.5, 3.5 Hz), 5.48–5.44 (1H, m), 2.19 (3H, s), 1.48 (3H, d, *J* = 1.5 Hz), 1.36 (3H, d, *J* = 1.0 Hz), 1.19 (1H, br t, *J* = 3.5 Hz); ^{13}C NMR (101 MHz, C_6D_6) δ_C 143.1, 142.8, 140.6, 137.6, 134.6, 130.2, 129.4, 128.7, 128.1, 127.7, 125.3, 112.6, 67.8, 25.6, 21.3, 18.0; HRMS (ESI+) *m/z* $[M+Na]^+$ calcd for $C_{16}H_{18}NaO_2$, 265.1199; found, 265.1198.

(R)-[(S*)-3,3-Dimethyloxiran-2-yl][2-(furan-3-yl)-5-methylphenyl]methanol (7)*

A solution of alcohol **6** (532 mg, 2.20 mmol) in dichloromethane (22 mL) was cooled to 0 °C and 3-chloroperbenzoic acid (523 mg, 76 wt %, 2.30 mmol) was added. The solution was stirred for 1 h then quenched with aqueous K_2CO_3 solution (10% w/v, 10 mL) and extracted with dichloromethane (3 × 10 mL). The combined organic layers were washed with brine (15 mL), dried (Na_2SO_4), filtered, and concentrated. The crude product was purified by flash chromatography (petrol/ether, 4:1) to afford the title compound (**7**) as a pale yellow oil (457 mg, 80%). R_f 0.10 (petrol/ether, 5:2); IR (thin film) ν_{max}/cm^{-1} 3427br m, 2964w, 1510 w, 1379w, 1167m, 1125w, 1048m, 1015s, 874s, 813s, 794s; ^{1}H NMR (400 MHz, $CDCl_3$) δ_H 7.49–7.46 (3H, m), 7.20 (1H, d, *J* = 7.5 Hz), 7.16 (1H, dd, *J* = 7.5, 1.0 Hz), 6.50 (1H, dd, *J* = 2.0, 1.0 Hz), 4.77 (1H, dd, *J* = 7.0, 3.0 Hz), 3.07 (1H, d, *J* = 7.0 Hz), 2.49 (1H, d, *J* = 3.0 Hz), 2.41 (3H, s), 1.25 (3H, s), 1.03 (3H, s); ^{13}C NMR (101 MHz, $CDCl_3$) δ_C 143.0, 140.1, 138.8, 138.1, 130.4, 129.0, 127.6, 124.6, 112.2, 68.9, 67.9, 60.7, 24.8, 21.4, 18.9 (one peak not resolved); HRMS (ESI+) *m/z* $[M+Na]^+$ calcd for $C_{16}H_{18}NaO_3$, 281.1148; found, 281.1148.

(5R,6R*)-4,4,8-Trimethyl-5,6-dihydro-4H-benzo[3,4]cyclohepta[1,2-b]furan-5,6-diol (**8**)*

Chlorotriisopropoxytitanium(IV) (6.0 mL, 1.0 M in hexanes, 6.0 mmol) was added to a solution of epoxide **7** (520 mg, 2.01 mmol) in dichloromethane (20 mL). The solution was stirred for 1 h then quenched with hydrochloric acid (20 mL, 1.0 M), stirred for 10 min, and extracted with ethyl acetate (3 × 20 mL). The combined organic layers were washed with brine (20 mL), dried (Na_2SO_4), filtered, and concentrated. The crude product was purified by flash chromatography (petrol/ether, 4:1) to afford the title compound (**8**) as a colourless solid (321 mg, 62%). R_f 0.30 (petrol/ether, 3:2); mp 115 °C (amorphous); IR (thin film) ν_{max}/cm^{-1} 3384br m, 2975m, 2934m, 1524m, 1499w, 1167s, 1071s, 1055s, 990m, 822 m, 750m, 733w; ^{1}H NMR (400 MHz, $CDCl_3$) δ_H 7.52 (1H, s), 7.40 (1H, d, *J* = 2.0 Hz), 7.37 (1H, d, *J* = 8.0 Hz), 7.10

(1H, br d, *J* = 8.0 Hz), 6.55 (1H, d, *J* = 2.0 Hz), 4.61 (1H, d, *J* = 8.5 Hz), 3.57 (1H, d, *J* = 8.5 Hz), 3.39 (1H, br s), 3.06 (1H, br s), 2.38 (3H, s), 1.45 (3H, s), 1.38 (3H, s); ^{13}C NMR (101 MHz, $CDCl_3$) δ_C 154.7, 141.6, 140.0, 136.5, 127.8, 126.9, 125.8, 123.6, 117.2, 110.1, 78.1, 69.3, 42.4, 27.8, 21.5, 20.8; HRMS (ESI+) *m/z* $[M+H]^+$ calcd for $C_{16}H_{19}O_3$, 259.1329; found, 259.1328.

*4,4,8-Trimethyl-4,6-dihydro-5H-benzo[3,4]cyclohepta[1,2-b]furan-5-one (**9**)*

A solution of *para*-toluenesulfonic acid monohydrate (22 mg, 0.12 mmol) and diol **8** (89 mg, 0.34 mmol) in benzene (2.6 mL) was heated at 60 °C for 18 h then cooled to RT, diluted with ether (8 mL) and washed with saturated aqueous $NaHCO_3$ solution (10 mL) then water (10 mL). The organic layer was dried (Na_2SO_4), filtered, and concentrated to afford the title compound (**9**) as a colourless solid which required no further purification (83 mg, quant.). R_f 0.90 (petrol/ether, 3:2); mp 92–94 °C; IR (thin film) ν_{max}/cm^{-1} 2981w, 2930w, 1713s, 1521w, 1285w, 1164w, 1070m, 821m, 756m; ^{1}H NMR (400 MHz, $CDCl_3$) δ_H 7.47 (1H, d, *J* = 2.0 Hz), 7.36 (1H, d, *J* = 8.0 Hz), 7.15 (1H, d, *J* = 8.0 Hz), 7.12 (1H, s), 6.62 (1H, d, *J* = 2.0 Hz), 3.80 (2H, s), 2.36 (3H, s), 1.45 (6H, s); ^{13}C NMR (101 MHz, $CDCl_3$) δ_C 207.1, 152.9, 142.0, 137.7, 130.5, 130.4, 128.6, 126.8, 119.1, 110.5, 50.4, 48.1, 30.5, 25.0, 21.2; HRMS (ESI+) *m/z* $[M+H]^+$ calcd for $C_{16}H_{17}O_2$, 241.1223; found, 241.1223.

*4,4,8-Trimethyl-5,6-dihydro-4H-benzo[3,4]cyclohepta[1,2-b]furan (**3**)*

Hydrazine hydrate (0.25 mL, 5.2 mmol) and KOH (94 mg, 1.7 mmol) were added to a solution of ketone **9** (135 mg, 0.562 mmol) in ethylene glycol (0.84 mL). The solution was heated at 130 °C for 2 h then at 180 °C for a further 20 h before being cooled to RT. Water (10 mL) was added, the solution was extracted with hexane (3 × 10 mL) and the combined organic layers were dried ($MgSO_4$), filtered, and concentrated to afford the title compound (**3**) as a colourless solid which required no further purification (101 mg, 79%). R_f 0.65 (petrol/ether, 3:2); mp 60–62 °C (amorphous); IR (thin film) ν_{max}/cm^{-1} 2962m, 2923s, 2857w, 1525m, 1455w, 1154m, 1068w, 931w, 891w, 820m, 756w, 736m; ^{1}H NMR (400 MHz, $CDCl_3$) δ_H 7.39 (1H, d, *J* = 8.0 Hz), 7.36 (1H, d, *J* = 2.0 Hz), 7.04 (1H, dd, *J* = 8.0, 1.0 Hz), 6.96 (1H, s), 6.58 (1H, d, *J* = 2.0 Hz), 2.79–2.74 (2H, m), 2.33 (3H, s), 1.85–1.80 (2H, m), 1.36 (6H, s); ^{13}C NMR (101 MHz, $CDCl_3$) δ_C 157.8, 141.6, 140.6, 135.7, 129.8, 129.1, 127.1, 127.0, 117.9, 110.2, 40.8, 38.1, 31.4, 29.9, 29.6, 21.1; HRMS (EI+) *m/z* $[M]^{\bullet+}$ calcd for $C_{16}H_{18}O$, 226.1352; found, 226.1353.

*3a-Hydroxy-4,4,8-trimethyl-3a,4,5,6-tetrahydro-2H-benzo[3,4]cyclohepta[1,2-b]furan-2-one (**10**)*

O_2 gas was bubbled through a solution of *N,N*-diisopropylethylamine (70 µL, 0.40 mmol), rose bengal (~4 mg), and tricycle **3** (20 mg, 0.088 mmol) in dichloromethane (8.3 mL) at 0 °C. The solution was irradiated for 5 min [unfiltered 160 W daylight 'Solar Glo Sun Simulating' lamp; www.exo-terra.com; lamp ~20 cm from reaction flask] then the O_2 flow was stopped and irradiation continued under an atmosphere of O_2 for 2.5 h. The solution was washed with saturated aqueous NH_4Cl solution (5 mL), dried (Na_2SO_4), filtered, and concentrated. The crude product was purified by flash chromatography (petrol/ether, 4:1 to 7:3) to afford the title compound (**10**) as an off-white solid (15.5 mg, 68%). R_f 0.15 (petrol/ether, 3:2); mp 154–156 °C (amorphous); IR (thin film) ν_{max}/cm^{-1} 3240br m, 2978w, 2935w, 1731s, 1623w, 1609w, 1266m, 1201m, 1176m, 1053m, 949m, 909w, 870m, 821m, 731s; ^1H NMR (400 MHz, $CDCl_3$) δ_H 7.23 (1H, d, J = 7.5 Hz), 7.07 (1H, d, J = 7.5 Hz), 7.01 (1H, s), 6.06 (1H, s), 3.38 (1H, br s), 3.00 (1H, t, J = 14.0 Hz), 2.67 (1H, dd, J = 14.0, 7.0 Hz), 2.35 (3H, s), 2.25 (1H, t, J = 14.0 Hz), 1.57 (1H, ddd, J = 14.0, 7.0, 2.0 Hz), 1.28 (3H, s), 1.10 (3H, s); ^{13}C NMR (101 MHz, $CDCl_3$) δ_C 170.6, 169.6, 141.5, 141.1, 131.5, 130.9, 127.6 (two peaks), 117.1, 110.5, 41.3, 38.2, 31.6, 24.9, 21.4, 21.3; HRMS (EI+) *m/z* $[M]^{\bullet+}$ calcd for $C_{16}H_{18}O_3$, 258.1250; found, 258.1253.

*4,4,8-Trimethyl-3a,4,5,6-tetrahydro-2H-benzo[3,4]cyclohepta[1,2-b]furan-2-one (**11**)*

$NaBH_4$ (8.0 mg, 0.21 mmol) was added to a solution of hydroxybutenolide **10** (13 mg, 0.05 mmol) and $CeCl_3{\cdot}7H_2O$ (2.0 mg, 5.4 µmol) in methanol (0.63 mL) at 0 °C. The solution was stirred at 0 °C for 80 min then quenched with concentrated hydrochloric acid (5.0 mL) and extracted with chloroform (3 × 5 mL). The organic extracts were washed with water (10 mL), dried ($MgSO_4$), filtered, and concentrated. The crude product was purified by flash chromatography (petrol/ether, 9:1 to 4:1) to afford the title compound (**11**) as a colourless oil (7.5 mg, 61%). R_f 0.25 (petrol/ether, 3:2); IR (thin film) ν_{max}/cm^{-1} 2920w, 1755s, 1662w, 1455w, 1315w, 1166w, 1025w, 917w, 854w, 819w; ^1H NMR (400 MHz, $CDCl_3$) δ_H 7.33 (1H, d, J = 8.0 Hz), 7.08 (1H, d, J = 8.0 Hz), 7.00 (1H, s), 6.17 (1H, d, J = 2.0 Hz), 4.87 (1H, d, J = 2.0 Hz), 2.93 (1H, dd, J = 16.0, 11.5 Hz), 2.71 (1H, ddd, J = 16.0, 8.0, 2.0 Hz), 2.35 (3H, s), 1.94 (1H, ddd, J = 14.5, 11.5, 2.0 Hz), 1.72 (1H, ddd, J = 14.5, 8.0, 2.0 Hz), 1.28 (3H, s), 0.93 (3H, s); ^{13}C NMR (101 MHz, $CDCl_3$) δ_C 173.3, 169.0, 141.8, 141.3, 131.5, 130.2, 128.0, 127.8, 115.6, 90.2, 40.0, 38.3, 31.3, 29.9, 21.4, 18.9; HRMS (ESI+) *m/z* $[M+H]^+$ calcd for $C_{16}H_{19}O_2$, 243.1380; found, 243.1380.

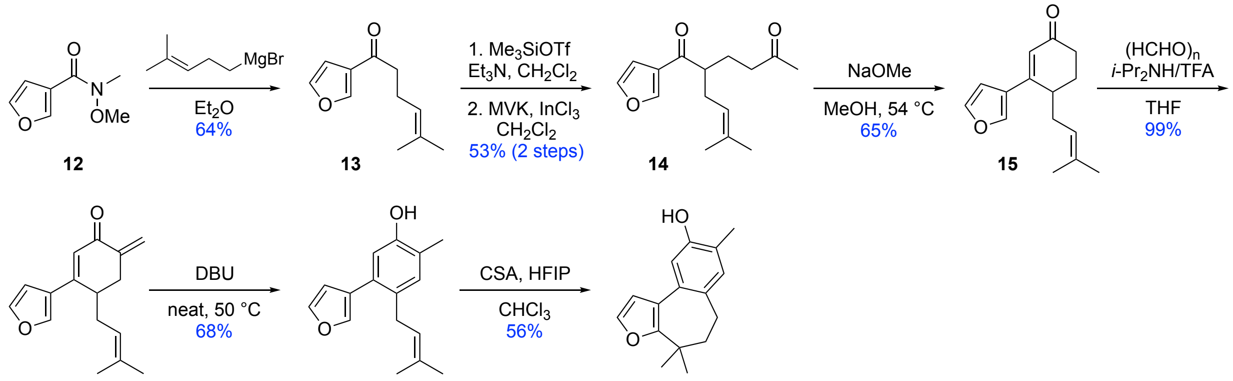

*1-(Furan-3-yl)-5-methylhex-4-en-1-one (**13**)*

I_2 (127 mg, 0.500 mmol) was added to a suspension of activated Mg turnings (758 mg, 31.2 mmol) in ether (3 mL). The mixture was warmed and stirred gently until the brown colour disappeared then a solution of 5-bromo-2-methylpent-2-ene (2.79 g, 17.1 mmol) in ether (15 mL) was added dropwise with vigorous stirring. The resulting mixture was heated to a gentle reflux for another 1 h before being cooled to 0 °C, then a solution of Weinreb amide **12** (2.42 g, 15.6 mmol) in ether (3 mL) was added dropwise. After stirring for 2 h, the mixture was quenched with saturated aqueous NH_4Cl solution (20 mL) and hydrochloric acid (5 mL, 1.0 M); the two phases were separated, and the aqueous phase was extracted with ether (3 × 20 mL). The combined extracts were washed with brine (30 mL), dried (Na_2SO_4), filtered, and concentrated. The crude product was purified by silica gel chromatography (pentane/ether, 4:1) to afford the title compound (**13**) (1.77 g, 64%) as a colourless oil. R_f 0.87 (pentane/ether, 3:1); IR (thin film) ν_{max}/cm^{-1} 3130w, 2967w, 1673s, 1563s, 1510s, 1154s; ^{1}H NMR (400 MHz, $CDCl_3$) δ_H 8.01 (1H, dd, *J* = 1.5, 1.0 Hz), 7.43 (1H, app t, *J* = 1.5 Hz), 6.77 (1H, dd, *J* = 2.0, 1.0 Hz), 5.13 (1H, tsept, *J* = 7.0, 1.5 Hz), 2.76 (2H, app t, *J* = 7.5 Hz), 2.39 (2H, br q, *J* = 7.5 Hz), 1.68 (3H, q, *J* = 1.5 Hz), 1.63 (3H, br s); ^{13}C NMR (101 MHz, $CDCl_3$) δ_C 194.9, 147.2, 144.2, 133.0, 127.9, 122.8, 108.8, 40.7, 25.8, 23.0, 17.8; HRMS (ESI+) *m/z* $[M+H]^+$ calcd for $C_{11}H_{15}O_2$, 179.1067; found, 179.1066.

*1-(Furan-3-yl)-2-(3-methylbut-2-en-1-yl)hexane-1,5-dione (**14**)*

To a stirred solution of ketone **13** (2.01 g, 11.3 mmol) in dichloromethane (35 mL) at 0 °C under Ar was added triethylamine (2.83 mL, 20.3 mmol) followed by trimethylsilyl triflate (2.15 mL, 12.4 mmol) dropwise. The mixture was allowed to warm slowly to RT and stirred for 15 h, after which the mixture was concentrated. The biphasic residual liquid was separated, the upper layer was retained and the lower layer was extracted with pentane (4 × 10 mL). The combined pentane extracts were combined with the upper layer, which was concentrated to give the silyl enol ether (2.63 g, 93%),

a pale yellow oil, as a 4:1 mixture of diastereomers, used directly in the next step without purification. IR (thin film) ν_{max}/cm^{-1} 3121w, 1618s, 1150s, 797s; ^{1}H NMR (400 MHz, C_6D_6) [major diastereomer] δ_H 7.44 (1H, br s), 6.98 (1H, t, J = 2.0 Hz), 6.26 (1H, dd, J = 2.0, 1.0 Hz), 5.29 (1H, tsept, J = 7.0, 1.5 Hz), 5.09 (2H, t, J = 7.0 Hz), 3.00 (2H, t, J = 7.0 Hz), 1.67–1.65 (3H, m), 1.61 (3H, br s), 0.15 (9H, s); ^{13}C NMR (101 MHz, C_6D_6) δ_C 143.3, 142.9, 139.6, 131.9, 123.4, 109.3, 108.5, 25.8, 25.4, 17.8, 0.6 [one resonance not resolved]; HRMS (ESI+) *m/z* $[M+H]^+$ calcd for $C_{14}H_{23}O_2Si$, 251.1462; found, 251.1463. A mixture of $InCl_3$ (47 mg, 0.21 mmol) and methyl vinyl ketone (0.96 mL, 0.12 mmol) was added dropwise to stirred solution of this crude silyl enol ether (2.63 g, 10.5 mmol) in dichloromethane (30 mL) at 0 °C under N_2. The mixture was stirred at 0 °C for 1 h, then water (50 mL) was added. The separated aqueous phase was extracted with ethyl acetate (3 × 20 mL) and the combined organic portions were washed with brine (50 mL), dried (Na_2SO_4), filtered, and concentrated. The crude product was purified by silica gel chromatography (pentane/ethyl acetate, 7:3) to afford the title compound (**14**) (1.49 g, 57%) as a colourless oil. R_f 0.61 (pentane/ethyl acetate, 7:3); IR (thin film) ν_{max}/cm^{-1} 3134w, 2927w, 1713s, 1669s, 1561s, 1510s, 1153s, 872s; ^{1}H NMR (400 MHz, $CDCl_3$) δ_H 8.01 (1H, s), 7.41 (1H, s), 6.74–6.72 (1H, m), 5.01 (1H, tsept, J = 7.5, 1.5 Hz), 3.02–2.93 (1H, m), 2.45 (1H, ddd, J = 17.5, 8.5, 6.0 Hz), 2.40–2.26 (2H, m, 1H), 2.19–2.08 (1H, m), 2.05 (3H, s), 2.00–1.89 (1H, m), 1.81–1.72 (1H, m), 1.60 (3H, s), 1.54 (3H, s); ^{13}C NMR (101 MHz, $CDCl_3$) δ_C 208.4, 198.3, 147.6, 144.4, 134.0, 128.2, 121.1, 108.8, 48.7, 40.9, 31.1, 30.0, 25.9, 25.8, 17.9; HRMS (ESI+) *m/z* $[M+H]^+$ calcd for $C_{15}H_{21}O_3$, 249.1485; found, 249.1491.

*3-(Furan-3-yl)-4-(3-methylbut-2-en-1-yl)cyclohex-2-en-1-one (**15**)*

Sodium methoxide (564 mg, 10.4 mmol) was added to a solution of diketone **14** (1.44 g, 5.80 mmol) in methanol (20 mL) and the resulting solution was stirred at 54 °C for 16 h. After cooling to RT, the mixture was diluted with hydrochloric acid (20 mL, 0.1 M) and ethyl acetate (20 mL). The aqueous phase was separated and extracted with ethyl acetate (3 × 10 mL). The combined organic portions were washed with brine (20 mL), dried (Na_2SO_4), filtered, and concentrated. The crude product was purified by silica gel chromatography (pentane/ethyl acetate, 3:2) to afford the title compound (**15**) (863 mg, 65%) as an orange oil. R_f 0.82 (pentane/ethyl acetate, 7:3); IR (thin film) ν_{max}/cm^{-1} 3121w, 2921w, 1658s, 1609s, 1161s; ^{1}H NMR (400 MHz, $CDCl_3$) δ_H 7.71 (1H, t, J = 1.5 Hz), 7.46 (1H, dd, J = 2.0, 1.5 Hz), 6.59 (1H, dd, J = 2.0 Hz), 6.20 (1H, s), 5.18 (1H, tsept, J = 7.5, 1.5 Hz), 2.72–2.64 (1H, m), 2.55 (1H, app dt, J = 17.0, 10.0 Hz), 2.39 (1H, dt, J = 17.0, 3.0 Hz), 2.31 (2H, t, J = 7.5 Hz), 2.13–2.07 (2H, m), 1.73 (3H, part-resolved q, J = 1.5 Hz), 1.62 (3H, s);

^{13}C NMR (101 MHz, $CDCl_3$) δ_C 199.3, 155.7, 144.4, 142.2, 134.0, 125.1, 122.9, 122.2, 107.9, 37.0, 32.7, 30.6, 25.8, 25.4, 18.0; HRMS (ESI+) *m/z* $[M+Na]^+$ calcd for $C_{15}H_{19}O_2$, 231.1380; found, 231.1380.

*3-(Furan-3-yl)-4-(3-methylbut-2-en-1-yl)-6-methylenecyclohex-2-en-1-one (**16**)*

Ketone **15** (115 mg, 0.500 mmol) was dissolved in a stirred suspension of diisopropylammonium trifluoroacetate (2.15 g, 10.0 mmol) in THF (8 mL) under N_2 then paraformaldehyde (45.0 mg, 1.50 mmol) and trifluoracetic acid (2.0 µL, 26 µmol) were added. This mixture was opened to the atmosphere and heated under reflux for 6 h, after which time it was cooled to RT and filtered. The filtrate was concentrated, dissolved in ether (10 mL), and hydrochloric acid (1 mL, 0.1 M) added. The layers were separated, and the ethereal layer was washed with brine (20 mL), dried (Na_2SO_4), and concentrated. The residue was purified by silica gel chromatography (pentane/ether 9:1) to afford the title compound (**16**) (120 mg, 99%) as a colourless crystalline solid. R_f 0.64 (pentane/ether, 3:2); mp 59−61 °C; IR (thin film) ν_{max}/cm^{-1} 2913w, 1656s, 1602s, 1158s, 873s; ^1H NMR (400 MHz, $CDCl_3$) δ_H 7.73 (1H, t, *J* = 1.0 Hz), 7.48 (1H, dd, *J* = 2.0, 1.5 Hz), 6.62 (1H, dd, *J* = 2.0, 1.0 Hz), 6.32 (1H, s), 6.15 (1H, t, *J* = 2.0 Hz), 5.34–5.31 (1H, m), 5.17–5.11 (1H, m), 2.88–2.78 (2H, m), 2.77–2.70 (1H, m), 2.29–2.13 (2H, m), 1.72 (3H, s), 1.56 (3H, s); ^{13}C NMR (101 MHz, $CDCl_3$) δ_C 188.3, 155.8, 144.7, 142.4, 140.5, 134.5, 125.3, 123.3, 122.0, 121.7, 108.0, 38.9, 34.8, 32.7, 25.9, 18.1; HRMS (ESI+) *m/z* $[M+H]^+$ calcd for $C_{16}H_{19}O_2$, 243.1380; found, 243.1379.

*5-(Furan-3-yl)-2-methyl-4-(3-methylbut-2-en-1-yl)phenol (**17**)*

Methylene ketone **16** (784 mg, 3.24 mmol) was dissolved in neat DBU (4.84 mL, 32.4 mmol) and the mixture was heated at 50 °C for 24 h then cooled to RT. Hydrochloric acid (35 mL, 1.0 M) was added, and the aqueous phase was separated and extracted with ethyl acetate (3 × 15 mL). The combined organic portions were dried (Na_2SO_4), filtered, and concentrated. The crude product was purified by silica gel chromatography (pentane/ethyl acetate, 4:1) to afford the title compound (**17**) (537 mg, 68%) as a yellow oil. R_f 0.70 (pentane/ethyl acetate, 4:1); IR (thin film) ν_{max}/cm^{-1} 3405br w, 2968w, 1512s, 1188s, 873s; ^1H NMR (400 MHz, $CDCl_3$) δ_H 7.46–7.44 (2H, m), 6.99 (1H, s), 6.73 (1H, s), 6.50 (1H, dd, *J* = 1.5, 1.0 Hz), 5.19 (1H, tsept, *J* = 7.0, 1.5 Hz), 4.64 (1H, s), 3.28 (2H, d, *J* = 7.0 Hz), 2.25 (3H, s), 1.72 (3H, part-resolved q, *J* = 1.5 Hz), 1.64 (3H, s); ^{13}C NMR (101 MHz, $CDCl_3$) δ_C 151.9, 142.6, 140.0, 132.2, 132.1, 132.0, 130.8, 125.2, 124.3, 123.1, 116.1, 111.9, 31.6, 25.9, 18.0, 15.6; HRMS (ESI+) *m/z* $[M+H]^+$ calcd for $C_{16}H_{19}O_2$, 243.1380; found, 243.1379.

*4,4,8-Trimethyl-5,6-dihydro-4H-benzo[3,4]cyclohepta[1,2-b]furan-9-ol (**18**)*

A solution of camphorsulfonic acid (7.0 mg, 0.030 mmol) in HFIP (2 mL) was added dropwise to a stirred solution of phenol derivative **17** (483 mg, 1.99 mmol) in chloroform (8 mL) at RT under N_2. Stirring was continued for 16 h, then saturated aqueous NH_4Cl solution (5 mL) was added. The aqueous phase was separated and extracted with ethyl acetate (3 × 5 mL) and the combined organic portions were dried (Na_2SO_4), filtered, and concentrated. The crude product was purified by silica gel chromatography (pentane/ethyl acetate, 4:1) to afford the title compound (**18**) (269 mg, 56%) as a colourless crystalline solid. R_f 0.79 (pentane/ethyl acetate, 4:1); mp 86−89 °C; IR (thin film) ν_{max}/cm^{-1} 3405br w, 2924w, 1512w, 1034s, 873s, 787s; ^1H NMR (400 MHz, $CDCl_3$) δ_H 7.35 (1H, d, J = 2.0 Hz), 6.93 (1H, s), 6.88 (1H, s), 6.53 (1H, d, J = 2.0 Hz), 2.73–2.69 (2H, m), 2.22 (3H, s), 1.83–1.78 (2H, m), 1.35 (6H, s); ^{13}C NMR (101 MHz, $CDCl_3$) δ_C 158.2, 152.1, 140.7, 134.3, 131.6, 130.8, 121.1, 117.6, 113.7, 110.1, 41.1, 38.1, 30.5, 29.6, 15.4; HRMS (ESI+) *m/z* $[M+H]^+$ calcd for $C_{16}H_{19}O_2$, 243.1380; found, 243.1380.

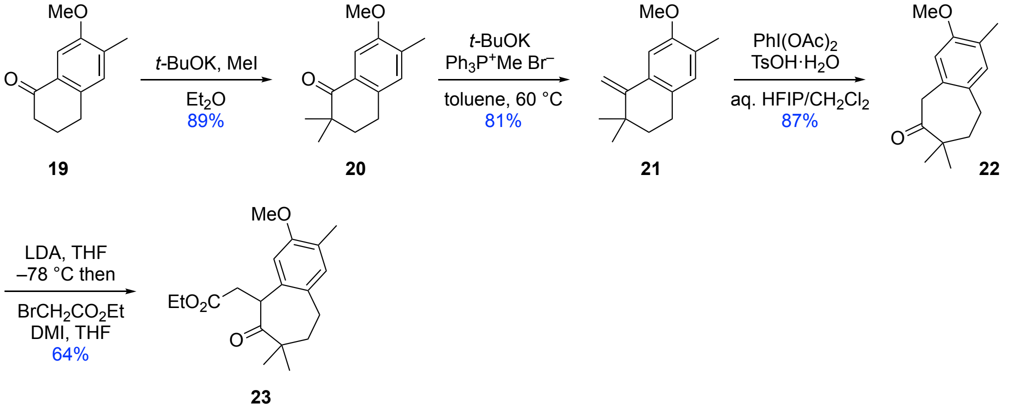

*7-Methoxy-2,2,6-trimethyl-3,4-dihydronaphthalen-1(2H)-one (**20**)*

Potassium *tert*-butoxide (1.82 g, 16.2 mmol) and ketone **19** (1.03 g, 5.41 mmol) were stirred in ether (30 mL) for 30 min. Iodomethane (1.18 mL, 19.0 mmol) was then added and the mixture was stirred for 14 h. The reaction was quenched with water (10 mL) and the mixture extracted with ether (3 × 20 mL). The combined organic extracts were washed with water (20 mL) and brine (20 mL), and were then dried (Na_2SO_4), filtered, and concentrated. The crude product was purified by silica gel chromatography (pentane/ethyl acetate, 9:1), affording ketone **20** as a colourless solid (1.05 g, 89%). R_f 0.64 (pentane/ethyl acetate, 9:1); mp 48–52 °C; IR (thin film) ν_{max}/cm^{-1} 2923w, 2360w, 1678s, 1610m, 1499m, 1260s, 1032m, 882w, 763w; ^1H NMR (400 MHz, $CDCl_3$) δ_H 7.45 (1H, s), 6.98 (1H, s), 3.85 (3H, s), 2.87 (2H, t, J = 6.5 Hz),

2.24 (3H, s), 1.95 (2H, t, J = 6.5 Hz), 1.20 (6H, s); ^{13}C NMR (101 MHz, $CDCl_3$) δ_C 203.0, 156.8, 136.0, 133.5, 130.8, 130.3, 107.6, 55.6, 41.4, 37.1, 25.0, 24.6, 16.7; HRMS (ESI+) *m/z* $[M+H]^+$ calcd for $C_{14}H_{19}O_2$, 219.1380; found, 219.1380.

*7-Methoxy-2,2,6-trimethyl-1-methylene-1,2,3,4-tetrahydronaphthalene (**21**)*

Potassium *tert*-butoxide (1.08 g, 9.62 mmol) and methyltriphenylphosphonium bromide (3.44 g, 9.63 mmol) were stirred in toluene (30 mL) for 1 h then a solution of ketone **20** (1.05 g, 4.81 mmol) in toluene (5 mL) was added, and the mixture was stirred at 60 °C for 14 h. The reaction was quenched with water (10 mL), the phases separated, and the aqueous phase was extracted with dichloromethane (3 × 30 mL). The combined organic portions were washed with water (20 mL) and brine (20 mL), and were then dried (Na_2SO_4), filtered, and concentrated. The crude product was purified by silica gel chromatography (pentane/ethyl acetate, 99:1), affording alkene **21** as a pale yellow oil (843 mg, 81%). R_f 0.39 (pentane/ethyl acetate, 99:1); IR (thin film) ν_{max}/cm^{-1} 2920w, 2851m, 2360w, 1501s, 1260s, 1209s, 1067m, 880s; ^{1}H NMR (400 MHz, $CDCl_3$) δ_H 7.05 (1H, s), 6.89 (1H, s), 5.42 (1H, s), 5.06 (1H, s), 3.86 (3H, s), 2.78 (2H, t, J = 6.5 Hz), 2.21 (3H, s), 1.68 (2H, t, J = 6.5 Hz), 1.17 (6H, s); ^{13}C NMR (101 MHz, $CDCl_3$) δ_C 156.2, 153.0, 133.5, 131.0, 128.2, 126.7, 106.3, 105.2, 55.5, 37.7, 34.8, 27.9, 25.8, 16.0; HRMS (ESI+) *m/z* $[M+H]^+$ calcd for $C_{15}H_{21}O$, 217.1587; found, 217.1588.

*3-Methoxy-2,7,7-trimethyl-5,7,8,9-tetrahydro-6H-benzo[7]annulen-6-one (**22**)*

Iodobenzene diacetate (2.62 g, 8.13 mmol) and *para*-toluenesulfonic acid monohydrate (0.93 g, 4.89 mmol) were stirred in a mixture of HFIP and dichloromethane (1:6, 50 mL) for 30 min. The reaction mixture was cooled to 0 °C, water (1.5 mL) was added, followed by alkene **21** (1.60 g, 7.40 mmol), and stirring was continued for 10 min at 0 °C. Saturated aqueous $NaHCO_3$ solution (30 mL) was added and the mixture extracted with dichloromethane (3 × 50 mL). The combined organic extracts were washed with saturated aqueous $NaHCO_3$ solution (50 mL) then dried ($MgSO_4$), filtered, and concentrated. The crude product was purified using silica gel chromatography (pentane/ethyl acetate, 92:8) to afford ketone **22** as a colourless solid (1.50 g, 87%). R_f 0.76 (pentane/ethyl acetate, 92:8); IR (thin film) ν_{max}/cm^{-1} 2924m, 2360w, 2341w, 1696s, 1503m, 1206s, 1094s, 1067s; ^{1}H NMR (400 MHz, $CDCl_3$) δ_H 6.86 (1H, s), 6.63 (1H, s), 3.80 (3H, s), 3.77 (2H, s), 2.90–2.87 (2H, m), 2.15 (3H, s), 1.80–1.77 (2H, m), 1.21 (6H, s); ^{13}C NMR (101 MHz, $CDCl_3$) δ_C 210.5,

156.6, 132.8, 131.7, 131.3, 125.1, 111.6, 55.6, 48.5, 46.6, 41.8, 29.8, 25.5, 15.8; HRMS (ESI+) *m/z* $[M+H]^+$ calcd for $C_{15}H_{21}O_2$, 233.1536; found, 233.1537.

*Ethyl 2-(3-methoxy-2,7,7-trimethyl-6-oxo-6,7,8,9-tetrahydro-5H-benzo[7]annulen-5-yl)acetate (**23**)*

Butyllithium (0.47 mL, 1.60 M solution in hexanes, 0.75 mmol) was added to a stirred solution of diisopropylamine (0.11 mL, 0.78 mmol) in THF (4 mL) at –10 °C and the mixture was stirred for 15 min then cooled to –78 °C. A solution of ketone **21** (116 mg, 0.500 mmol) in THF (1 mL) was added dropwise, then the mixture was warmed to 0 °C over 2 h. Ethyl bromoacetate (0.066 mL, 0.60 mmol) and 1,3-dimethyl-2-imidazolidinone (DMI; 0.065 mL, 0.60 mmol) were added, and the resulting mixture was warmed to RT and stirred for a further 16 h. The mixture was quenched with saturated aqueous NH_4Cl solution (15 mL) and extracted with ether (3 × 5 mL). The combined organic extracts were washed with brine (20 mL), dried (Na_2SO_4), filtered, and concentrated. The residue was purified using silica gel chromatography (pentane/ethyl acetate, 9:1) to afford the title compound (**23**) (102 mg, 64%) as a pale yellow oil. R_f 0.60 (pentane/ethyl acetate, 9:1); IR (thin film) ν_{max}/cm^{-1} 2979w, 1730s, 1703s, 1180s, 1079s; ^{1}H NMR (400 MHz, $CDCl_3$) δ_H 6.88 (1H, s), 6.54 (s, 1H), 4.83 (1H, dd, *J* = 11.0, 4.0 Hz), 4.14 (2H, q, *J* = 7.0 Hz), 3.80 (3H, s), 3.49 (1H, dd, *J* = 16.5, 11.0 Hz), 3.28 (1H, app t, *J* = 14.0 Hz), 2.72 (1H, dd, *J* = 16.5, 4.0 Hz), 2.61 (1H, ddd, *J* = 14.0, 7.0, 2.0 Hz), 2.14 (3H, s), 1.92 (1H, ddd, *J* = 14.0, 7.0, 2.0 Hz), 1.61–1.52 (1H, m, part-obscured by H_2O peak), 1.50 (3H, s), 1.27 (3H, t, *J* = 7.0 Hz), 1.04 (3H, s); ^{13}C NMR (101 MHz, $CDCl_3$) δ 210.6, 172.2, 156.9, 134.2, 133.1, 131.5, 125.3, 107.2, 60.8, 55.6, 48.8, 45.5, 44.1, 33.6, 29.8, 26.6, 25.1, 15.8, 14.4; HRMS (ESI+) *m/z* $[M+H]^+$ calcd for $C_{19}H_{27}O_4$, 319.1904; found, 319.1903.

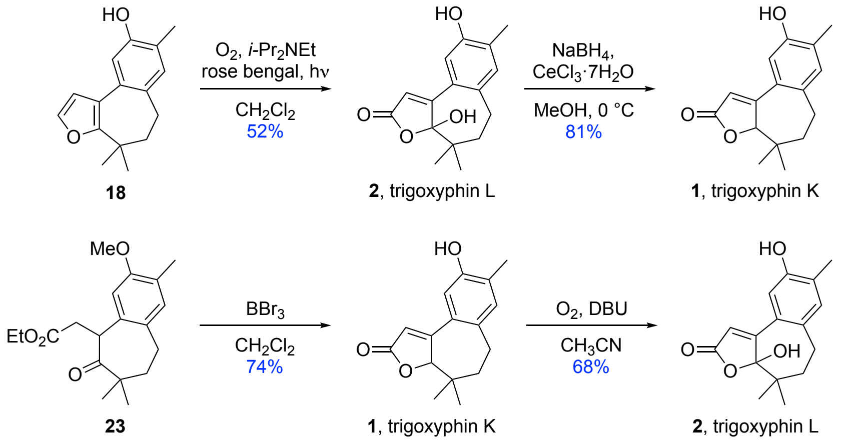

*Trigoxyphin K (**1**)*

Method 1 (from trigoxyphin L) $NaBH_4$ (14 mg, 0.37 mmol) then $CeCl_3·7H_2O$ (3.5 mg, 9.4 μmol) were added to a solution of trigoxyphin L (25.0 mg, 0.091 mmol) in methanol (3 mL) at 0 °C. The solution was stirred at 0 °C for 4 h then the reaction was quenched with hydrochloric acid (8.0 mL, 0.1 M). The mixture was extracted with ethyl acetate (3 × 5 mL) and the combined organic extracts were washed with brine (15 mL), dried (Na_2SO_4), filtered, and concentrated. The crude product was purified by flash chromatography (pentane/ethyl acetate, 2:3) to afford trigoxyphin K (19 mg, 81%) as a colourless crystalline solid.

Method 2 (from ketoester 23) BBr_3 solution (0.30 mL, 1.0 M in dichloromethane, 0.30 mmol) was added to a stirred solution of ketoester **23** (31 mg, 0.10 mmol) in dichloromethane (2 mL) at RT. The mixture was stirred for 15 h then water (5 mL) was added. The separated aqueous layer was extracted with dichloromethane (3 × 5 mL) then the combined organic portions were washed with brine (20 mL), dried (Na_2SO_4), filtered, and concentrated. The residue was purified using silica gel chromatography (pentane/ethyl acetate, 2:3) to afford trigoxyphin K (19 mg, 74%) as a colourless crystalline solid. R_f 0.73 (pentane/ethyl acetate, 2:3); mp 146−148 °C (no lit. mp available); IR (thin film) ν_{max}/cm^{-1} 2971w, 1713s, 1597w, 949s, 873s; ^1H NMR (400 MHz, $CDCl_3$) δ_H 6.94 (1H, s), 6.86 (1H, s), 6.15 (d, J = 1.5 Hz), 5.11 (1H, br s), 4.88 (d, J = 1.5 Hz), 2.85 (1H, dd, J = 16.0, 11.0 Hz), 2.66 (1H, ddd, J = 16.0, 8.0, 2.0 Hz), 2.25 (3H, s), 1.95–1.88 (1H, m), 1.74–1.62 (1H, m), 1.27 (3H, s), 0.91(3H, s); ^{13}C NMR (101 MHz, $CDCl_3$) δ_C 174.1, 169.8, 153.3, 133.9, 133.5, 128.6, 128.2, 115.7, 114.8, 90.7, 40.3, 38.2, 30.3, 29.8, 19.1, 15.9;* HRMS (ESI+) m/z $[M+H]^+$ calcd for $C_{16}H_{19}O_3$, 259.1329; found, 259.1333.

* Lit. ^{13}C NMR (100 MHz, $CDCl_3$) δ_C 173.4, 169.4, 153.0, 134.1, 133.6, 128.8, 127.8, 115.7, 115.2, 90.5, 40.3, 38.2, 30.3, 29.8, 19.0, 15.9. Resonances in both the ^1H and ^{13}C NMR spectra show minor shifts depending on the sample purity and concentration. See spectra pp. S15–S16.

*Trigoxyphin L (**2**)*

Method 1 (from tricyclic furan 18) *N,N*-Diisopropylethylamine (0.290 mL, 1.66 mmol) and rose bengal (27 mg, 27 µmol) were added to a stirred solution of tricyclic furan derivative **18** (134 mg, 0.553 mmol) in dichloromethane (6 mL) at RT. O_2 gas was bubbled through the solution for 15 min then bubbling was stopped and the stirred mixture was irradiated [unfiltered 160 W daylight 'Solar Glo Sun Simulating' lamp; www.exo-terra.com; lamp ~20 cm from reaction flask] under an O_2 atmosphere for 18 h. The mixture was diluted with saturated aqueous NH_4Cl solution (10 mL) and extracted with ethyl acetate (3 × 5 mL). The combined organic extracts was dried (Na_2SO_4), filtered, and concentrated. The crude product was purified by silica gel chromatography (pentane/ethyl acetate, 2:3) to afford trigoxyphin L (78.5 mg, 52%) as a colourless crystalline solid.

Method 2 (from trigoxyphin K) O_2 gas was bubbled through a solution of trigoxyphin K (11.0 mg, 4.26 µmol) and DBU (9.5 µL, 6.4 µmol) in acetonitrile (1 mL) for 15 min at RT. The O_2 flow was stopped and the mixture was stirred under the O_2 atmosphere for 18 h. Saturated aqueous NH_4Cl solution (5 mL) was added, then hydrochloric acid (1.0 M) was added dropwise until the pH = 4. The layers were separated and the aqueous layer was extracted with ethyl acetate (3 × 5 mL). The combined organic extracts were dried (Na_2SO_4), filtered, and concentrated. The crude product was purified by silica gel chromatography (pentane/ethyl acetate, 2:3) to afford trigoxyphin L (8.0 mg, 68%) as an off-white solid. R_f 0.58 (pentane/ethyl acetate, 2:3); mp 197−199 °C (no lit. mp available); IR (thin film) ν_{max}/cm^{-1} 3370br w, 2980w, 1735s, 1618w, 1252s, 952s, 859s; ^1H NMR (400 MHz, CD_3OD) δ_H 6.89 (1H, s), 6.79 (1H, s), 6.05 (1H, s), 2.89 (1H, t, *J* = 15.0 Hz), 2.62 (1H, dd, *J* = 15.0, 6.5 Hz), 2.26–2.16 (1H, m) overlaying 2.17 (3H, s), 1.50 (1H, dd, *J* = 14.0, 6.0 Hz), 1.22 (3H, s), 1.03 (3H, s); ^1H NMR (400 MHz, $(CD_3)_2CO$) δ_H 8.31 (1H, br s), 6.93 (1H, s), 6.81 (1H, s), 6.19 (1H, br s), 6.02 (1H, s), 2.90 (1H, t, *J* = 14.5 Hz), 2.62 (1H, dd, *J* = 14.5, 5.5 Hz), 2.23 (1H, t, *J* = 12.5 Hz), 2.18 (3H, s), 1.52 (1H, dd, *J* = 12.5, 5.5 Hz), 1.22 (3H, s), 1.03 (3H, s); ^{13}C NMR (101 MHz, CD_3OD) δ_C 173.3, 172.3, 154.9, 134.0, 133.3, 130.0, 128.8, 117.6, 117.0, 42.0, 39.7, 31.6, 25.6, 21.5, 16.0;[†] ^{13}C NMR (101 MHz, $(CD_3)_2CO$) δ_C 170.6, 170.3, 154.4, 133.7, 132.8, 130.0, 127.7, 117.6, 117.5, 111.3, 41.7, 39.2, 31.2, 25.5, 21.3, 15.9. HRMS (ESI+) *m/z* $[M+H]^+$ calcd for $C_{16}H_{19}O_4$, 275.1278; not found.

[†] Lit. ^{13}C NMR (100 MHz, CD_3OD) δ_C 173.3, 172.3, 154.9, 134.0, 133.3, 130.0, 128.8, 117.6, 117.0, 42.0, 39.7, 31.6, 25.6, 21.5, 16.0. In NMR spectra of relatively concentrated samples in methanol-d_4, ^1H resonances are broad and some ^{13}C resonances are of low intensity; the ^1H resonances sharpen and the relative intensities of ^{13}C peaks are restored in dilute solution. In acetone-d_6 the ^1H NMR resonances remain resolved in relatively concentrated samples and the resonances in the ^{13}C NMR spectrum are all present and of reasonable intensity. See spectra pp. S17–S19.

^{1}H NMR (400 MHz, CDCl$_3$) (1), trigoxyphin K *(concentrated sample + minor intermediate)*

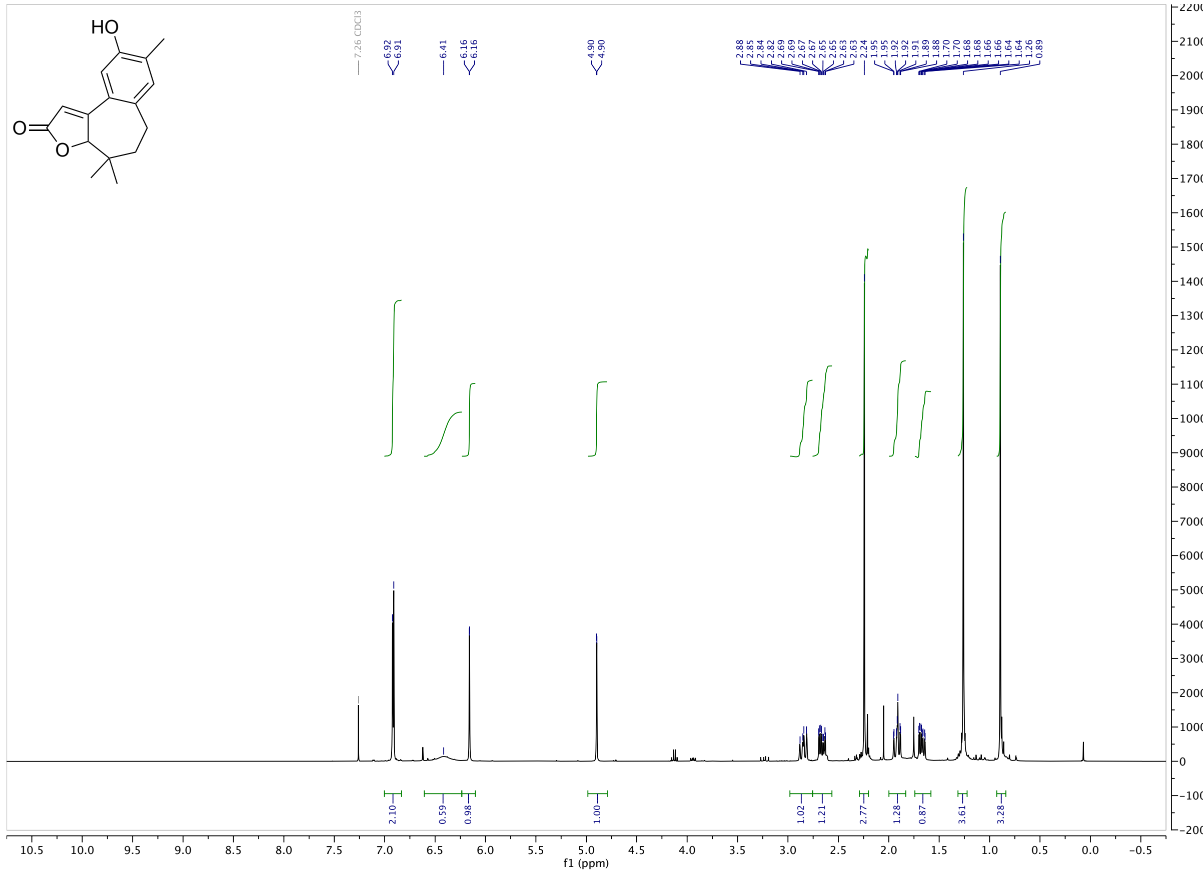

^{1}H NMR (400 MHz, CDCl$_3$) (1), trigoxyphin K *(less concentrated sample)*

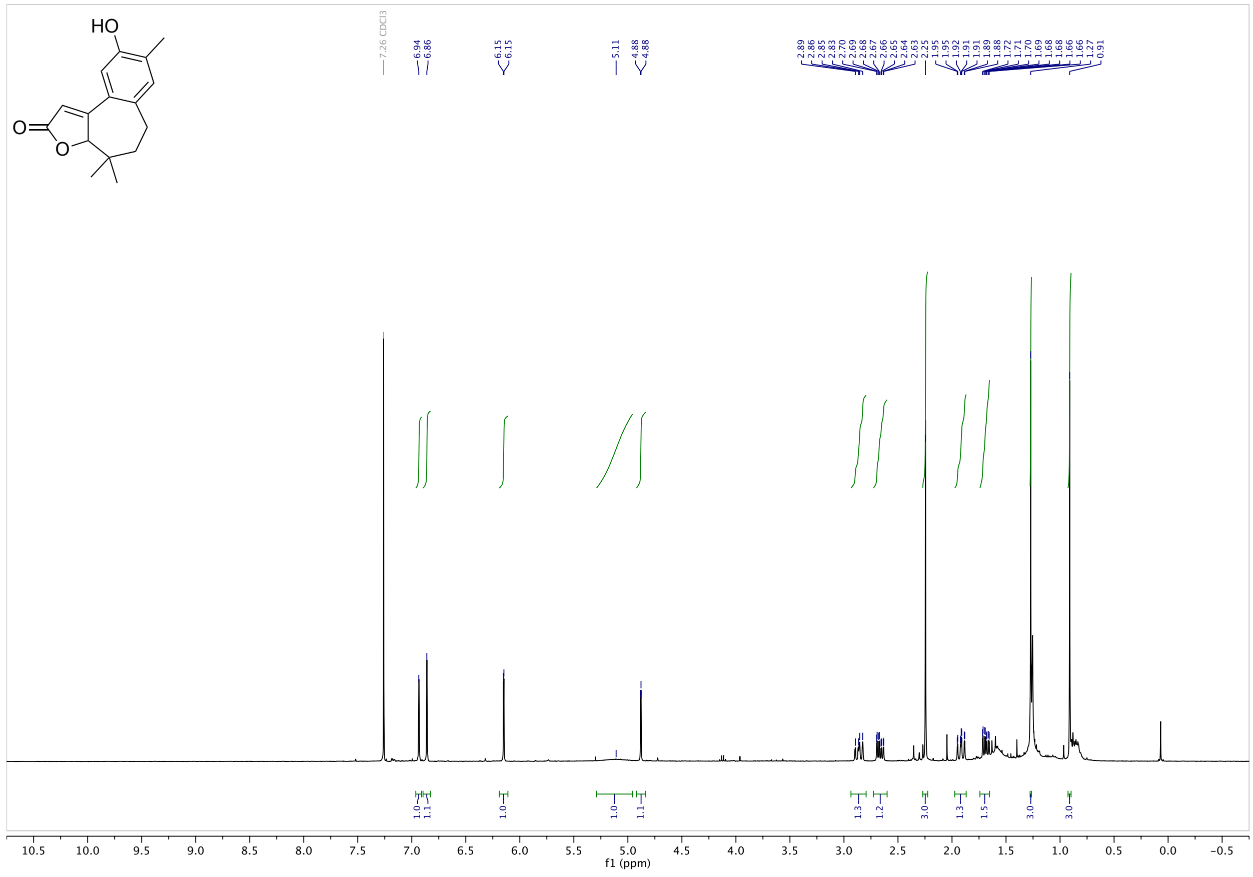

^{13}C NMR (101 MHz, $CDCl_3$) (1), trigoxyphin K *(+ minor intermediate)*

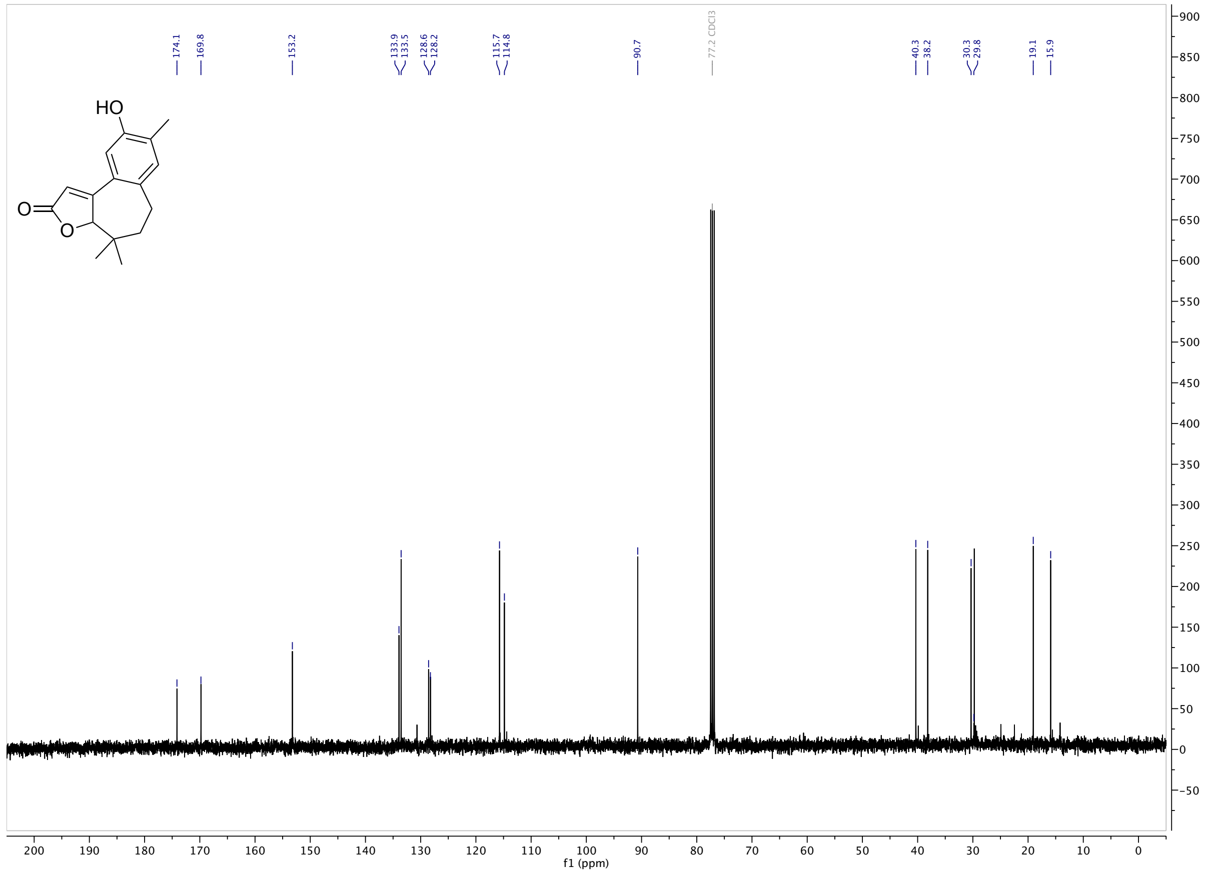

^{1}H NMR (400 MHz, CD_3OD) (2), trigoxyphin L *(strong sample as in isolation paper)*

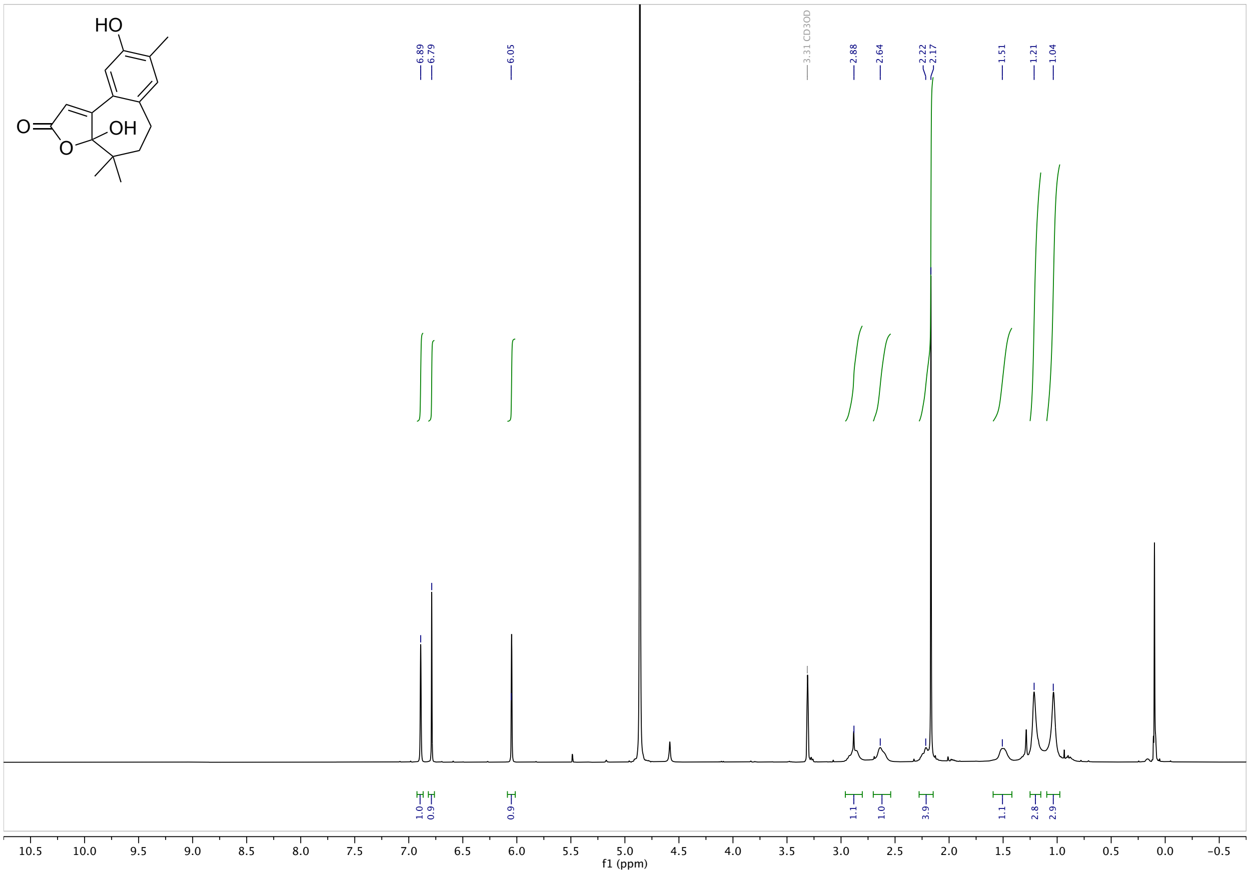

^{13}C NMR (101 MHz, CD_3OD) (2), trigoxyphin L *(strong sample as in isolation paper)*

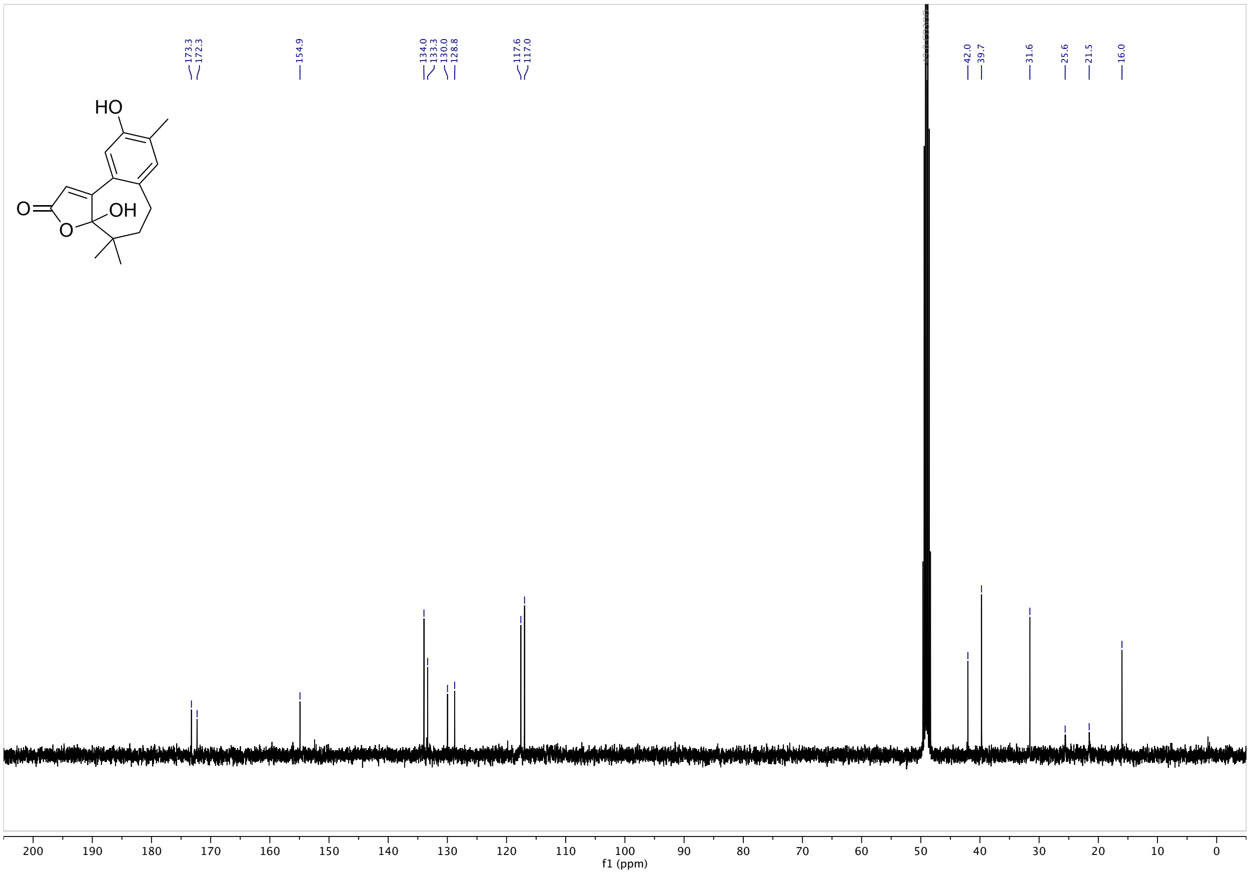

^{1}H NMR (400 MHz, CD_3OD) (2), trigoxyphin L *(weak sample)*

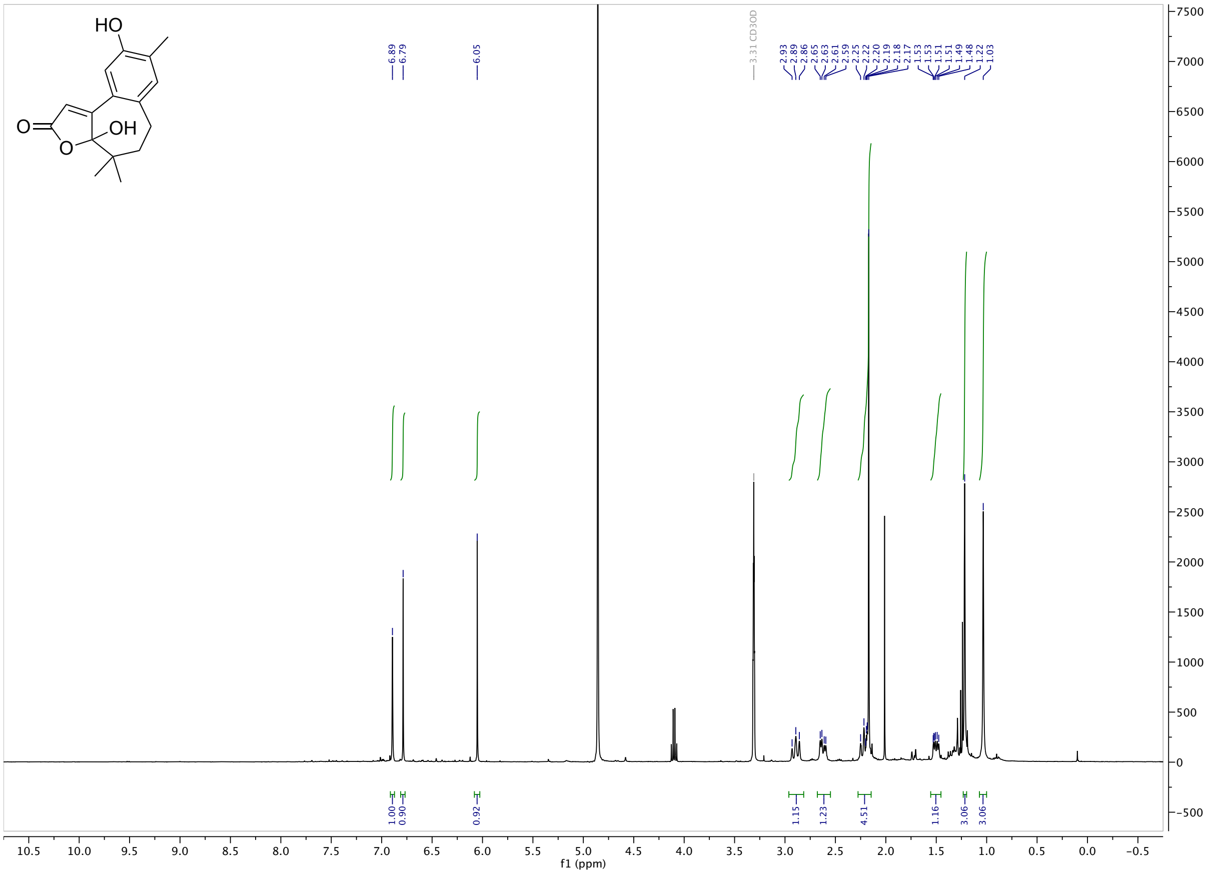

^{13}C NMR (101 MHz, CD_3OD) (2), trigoxyphin L *(weak sample)*

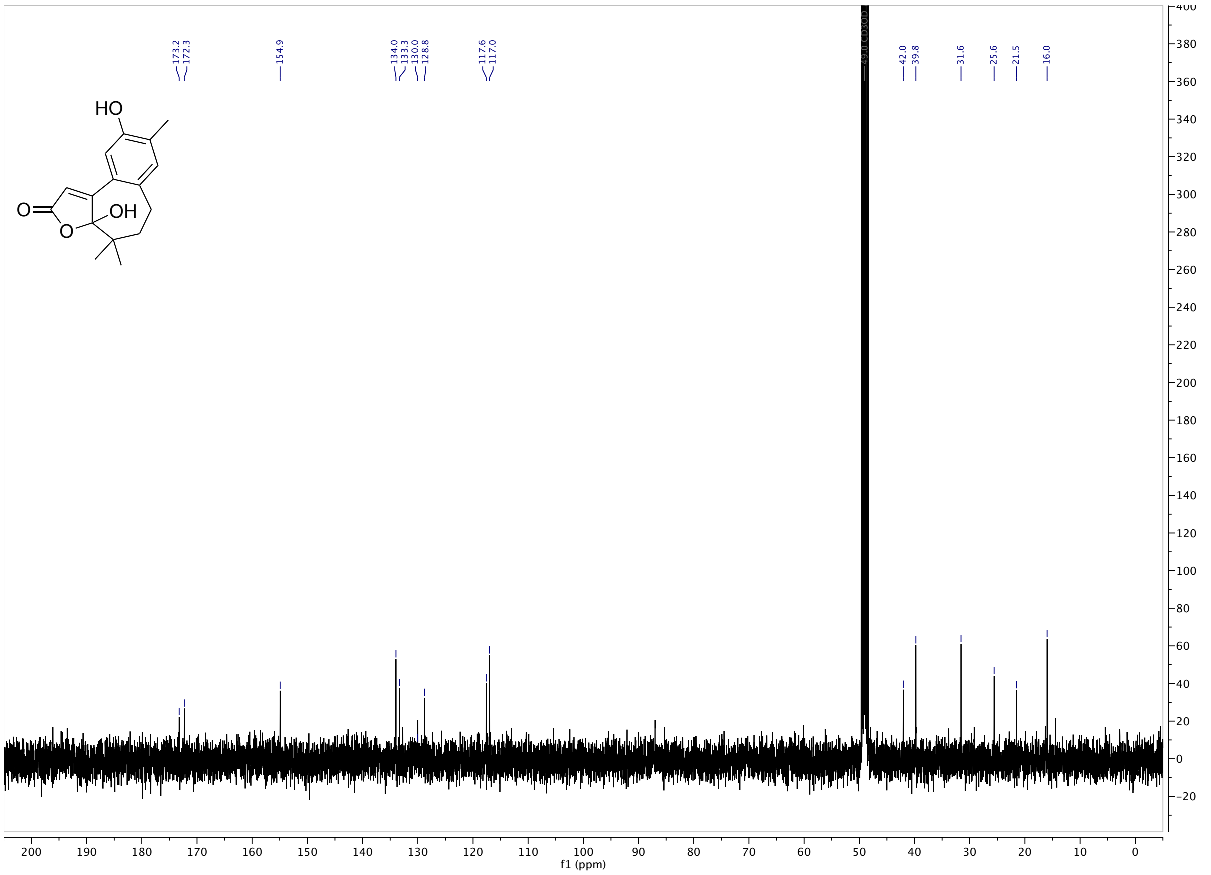

^{1}H NMR (400 MHz, $(CD_3)_2CO$) (2), trigoxyphin L

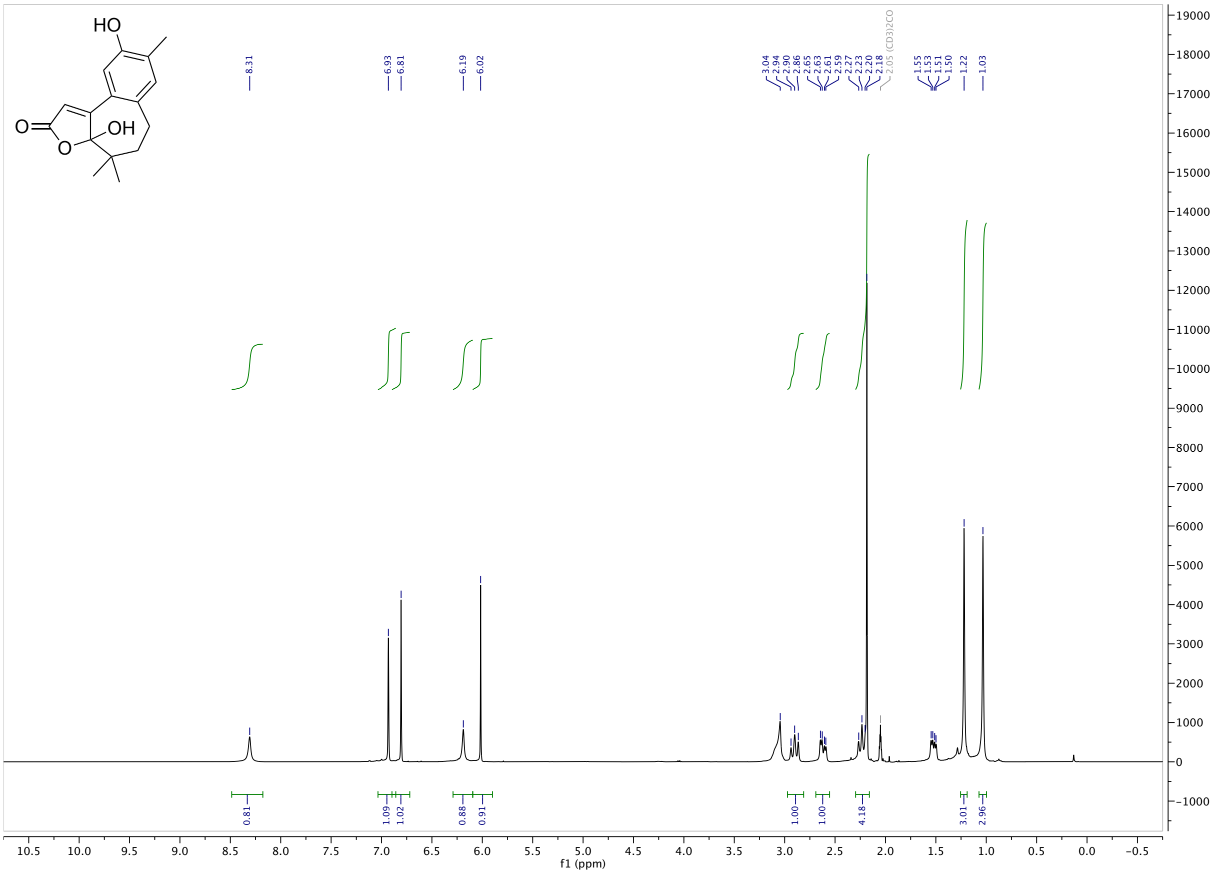

^{13}C NMR (101 MHz, $(CD_3)_2CO$) (2), trigoxyphin L

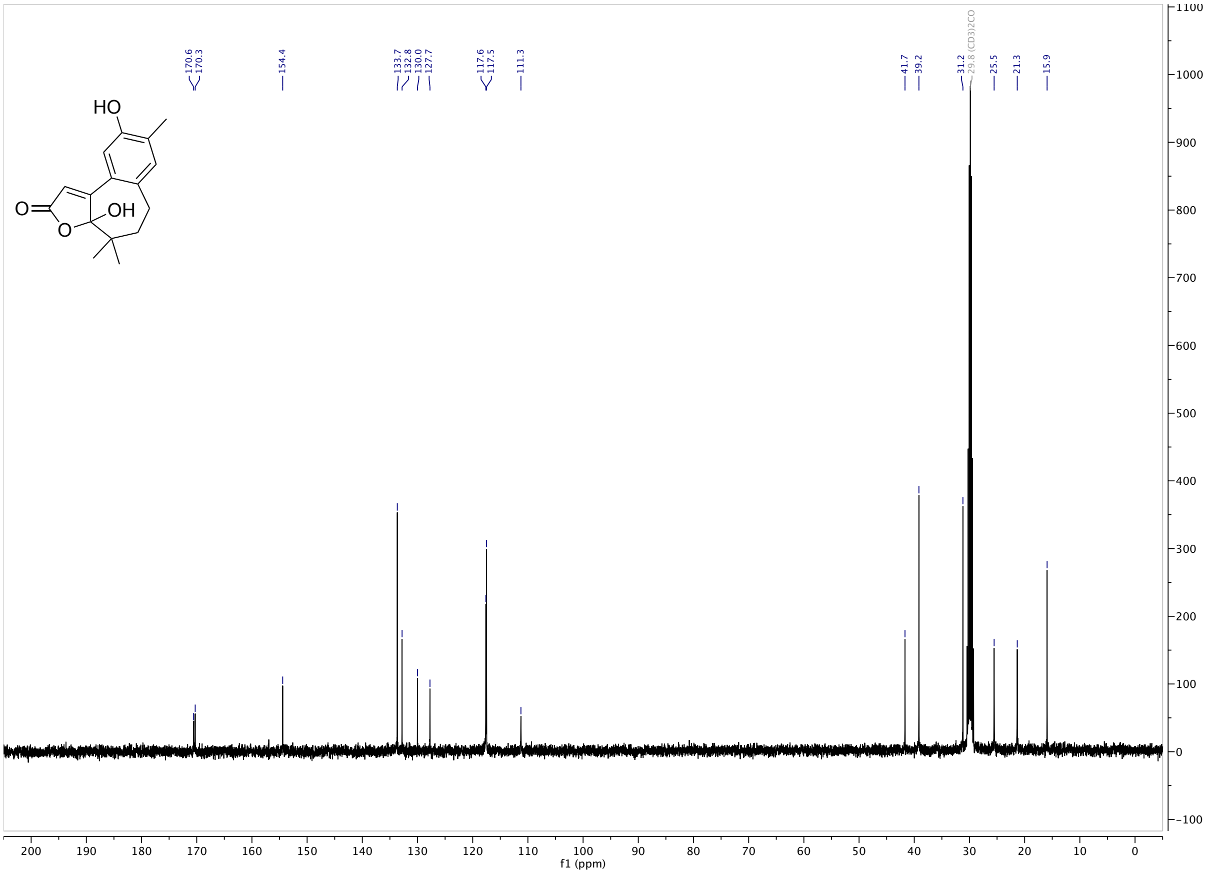

^{1}H NMR (400 MHz, CDCl$_3$) (3)

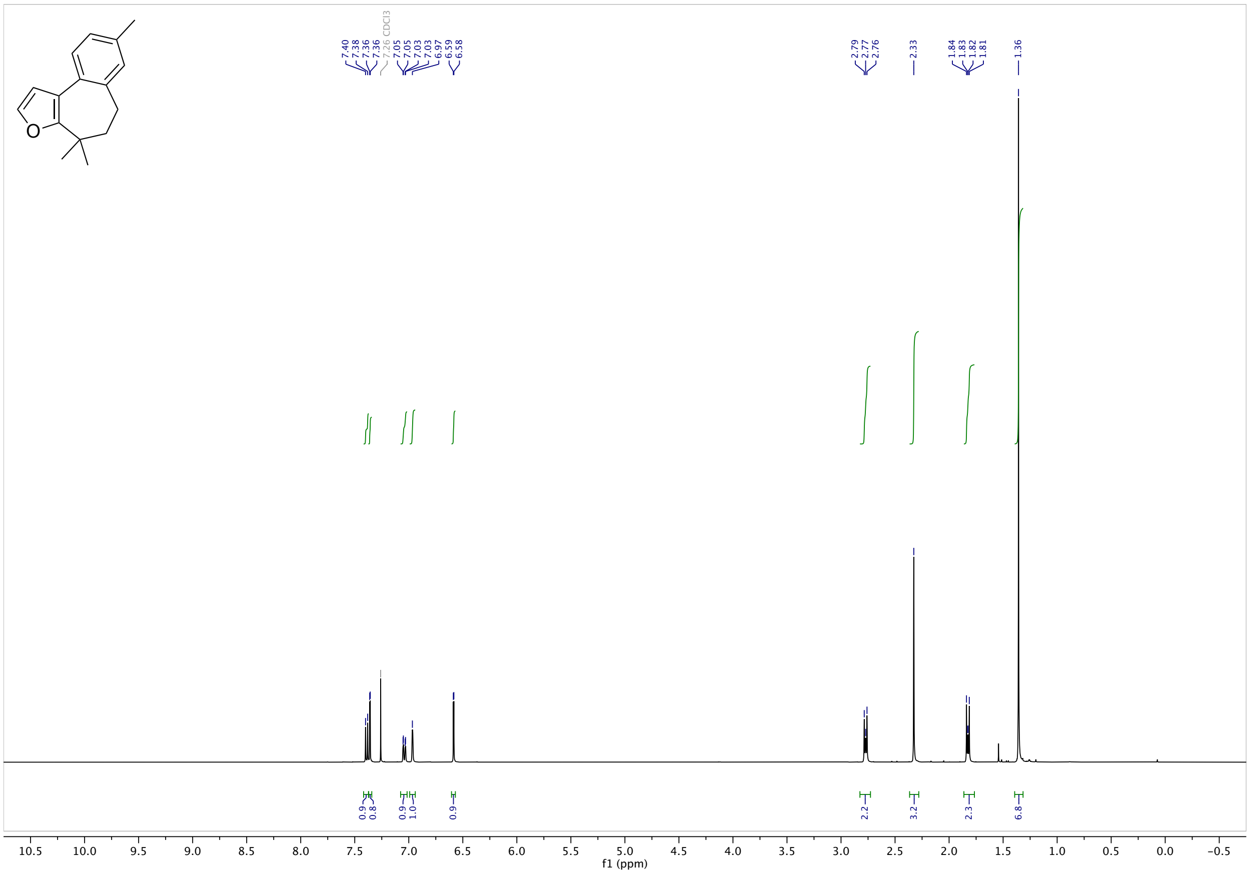

^{13}C NMR (101 MHz, CDCl$_3$) (3)

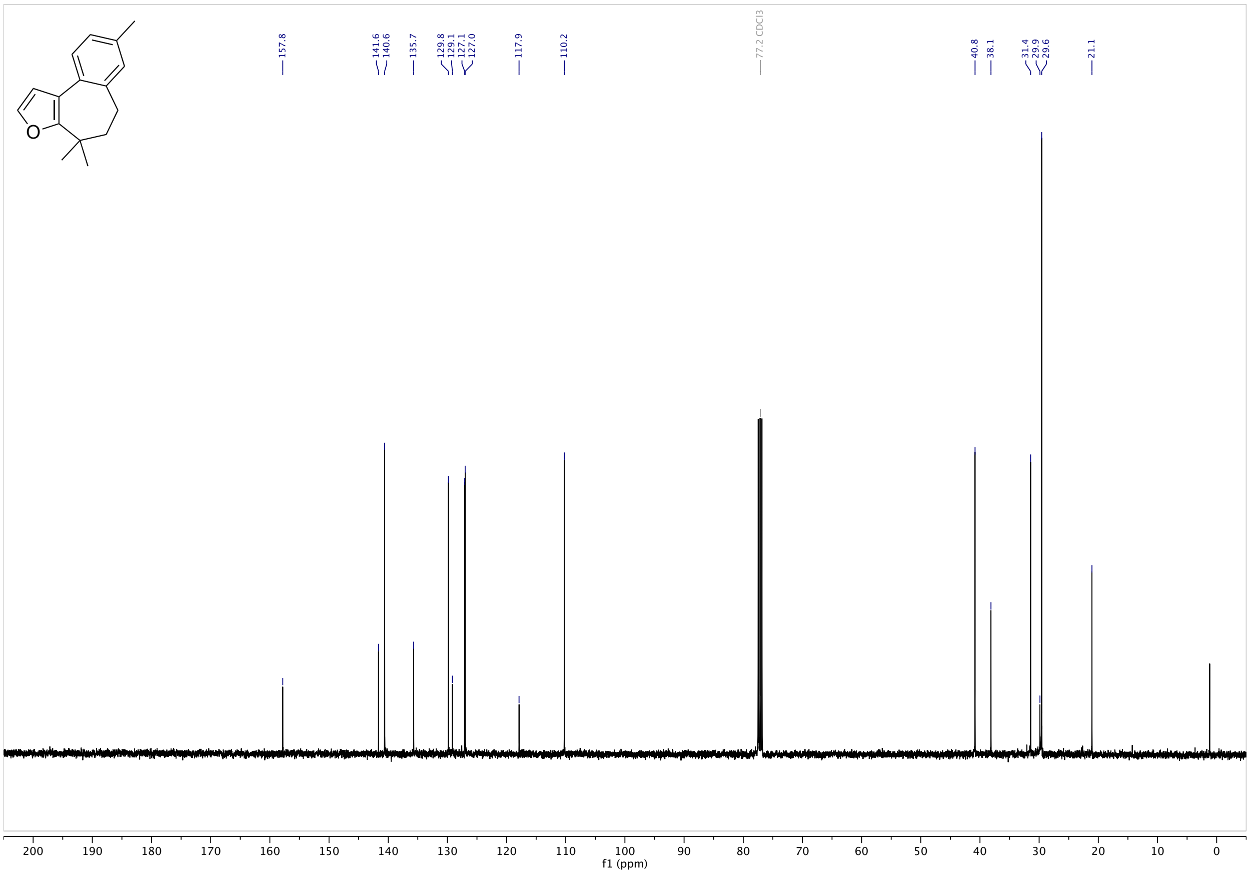

^{1}H NMR (400 MHz, $CDCl_3$) (5)

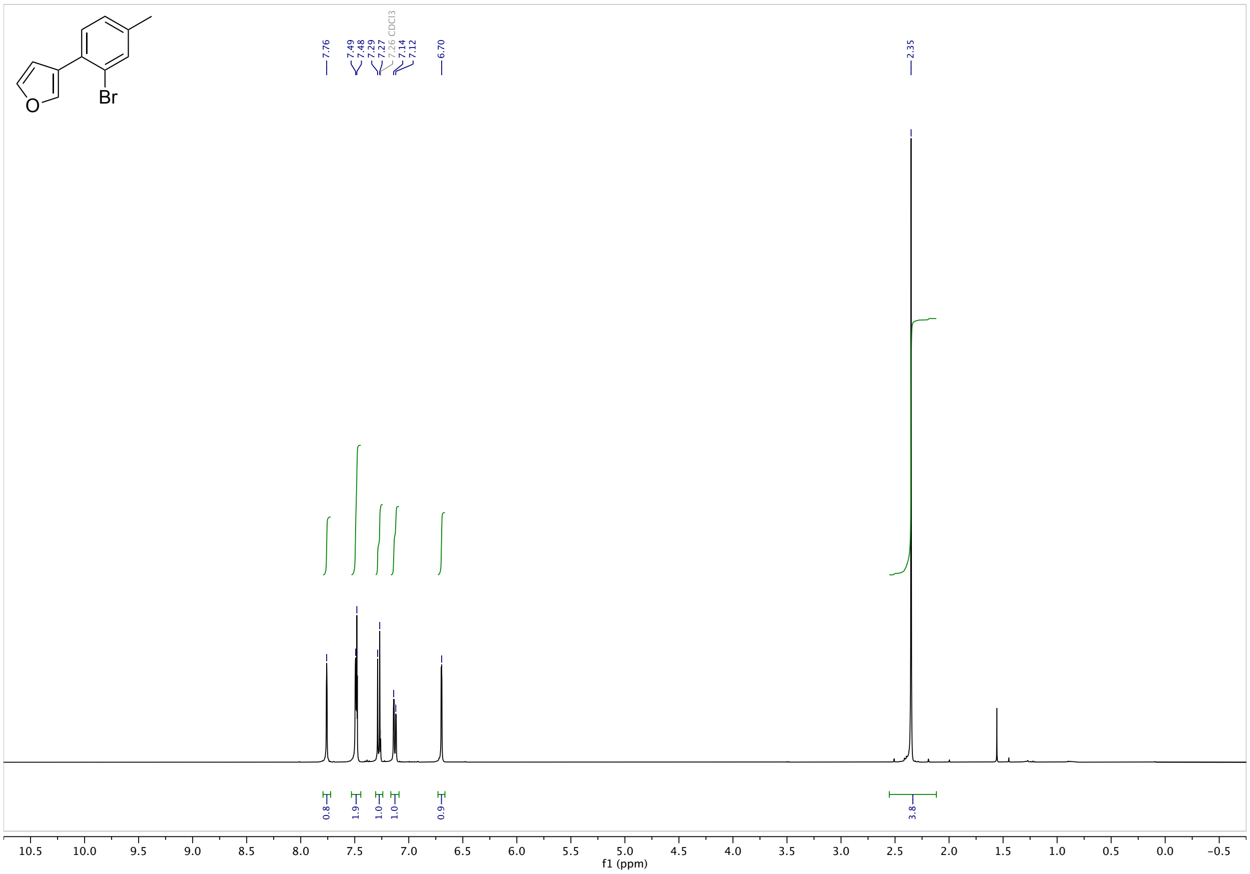

^{13}C NMR (101 MHz, $CDCl_3$) (5)

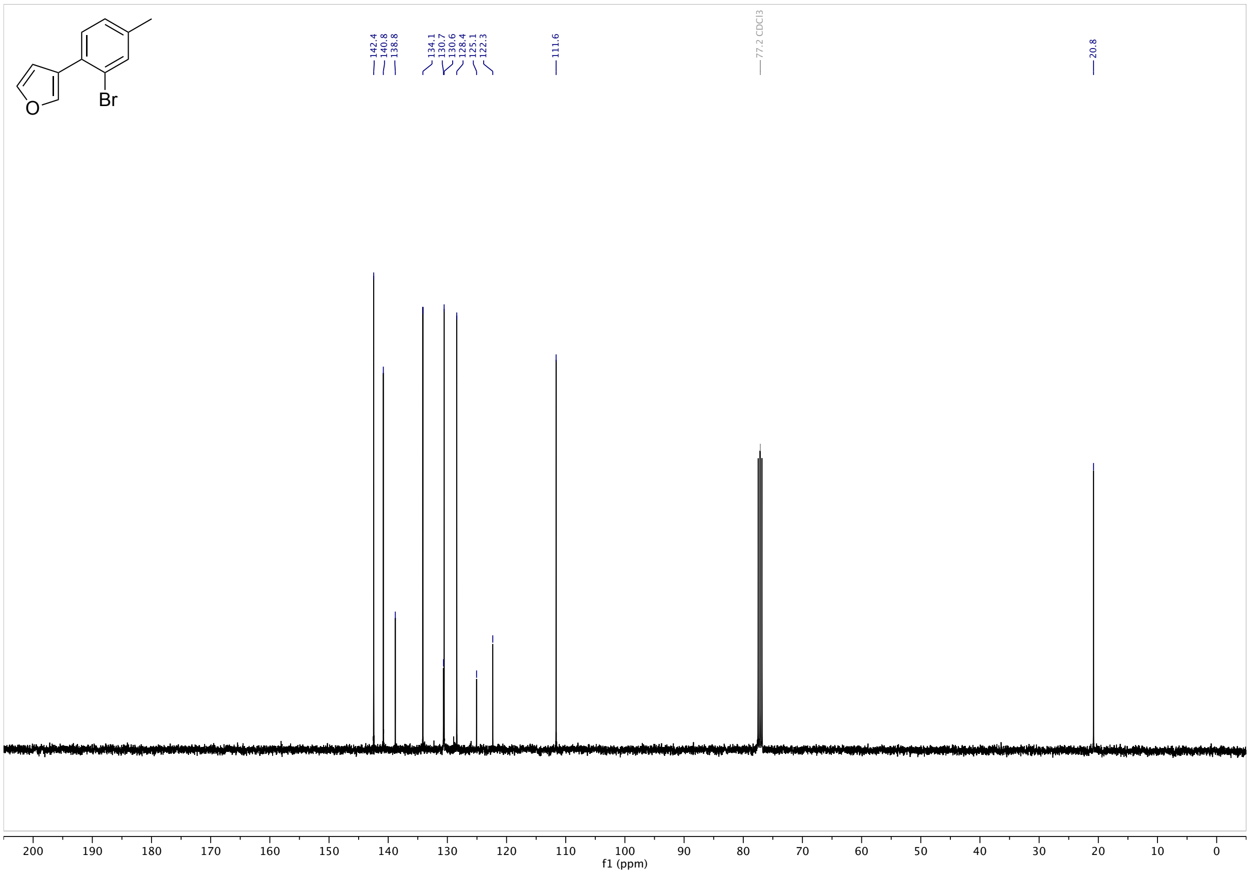

^{1}H NMR (400 MHz, C_6D_6) (6)

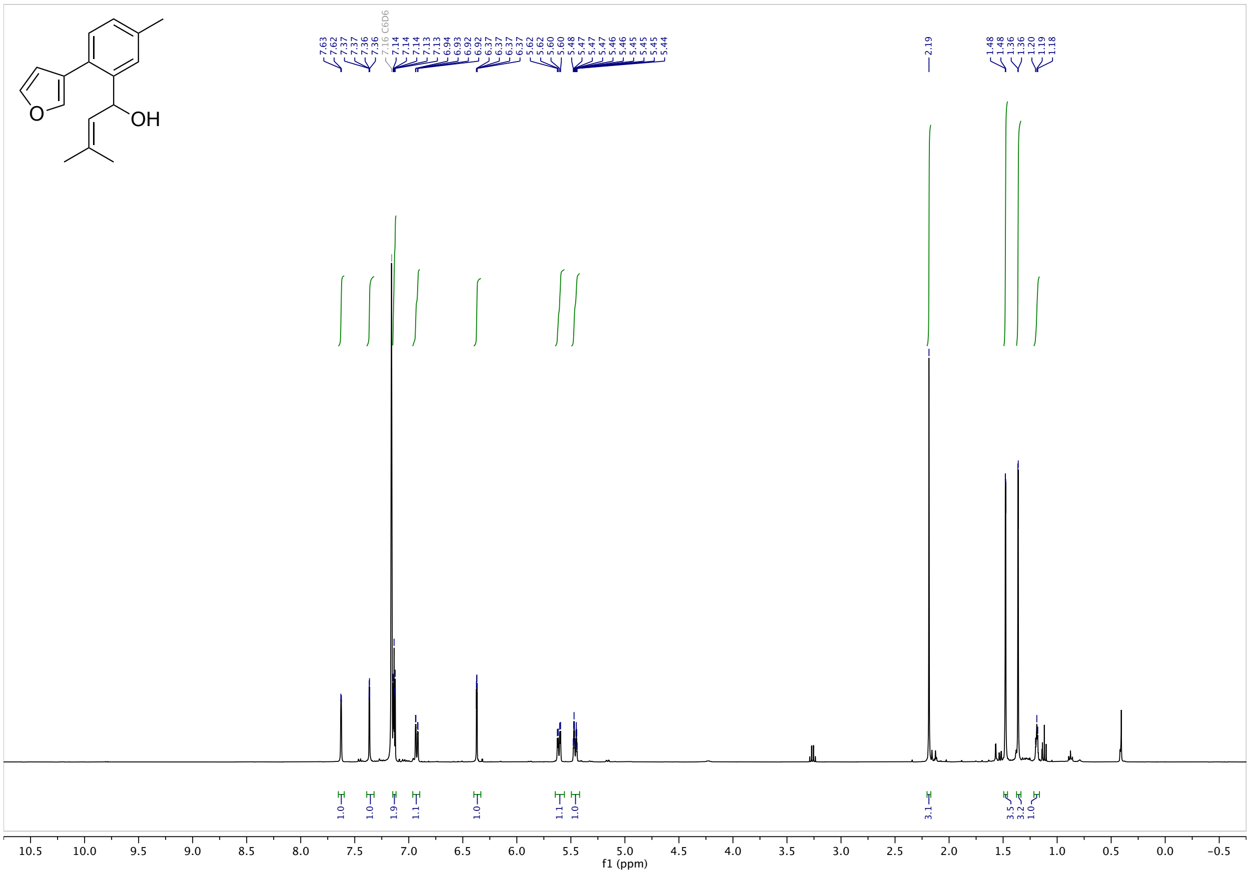

^{13}C NMR (101 MHz, C_6D_6) (6)

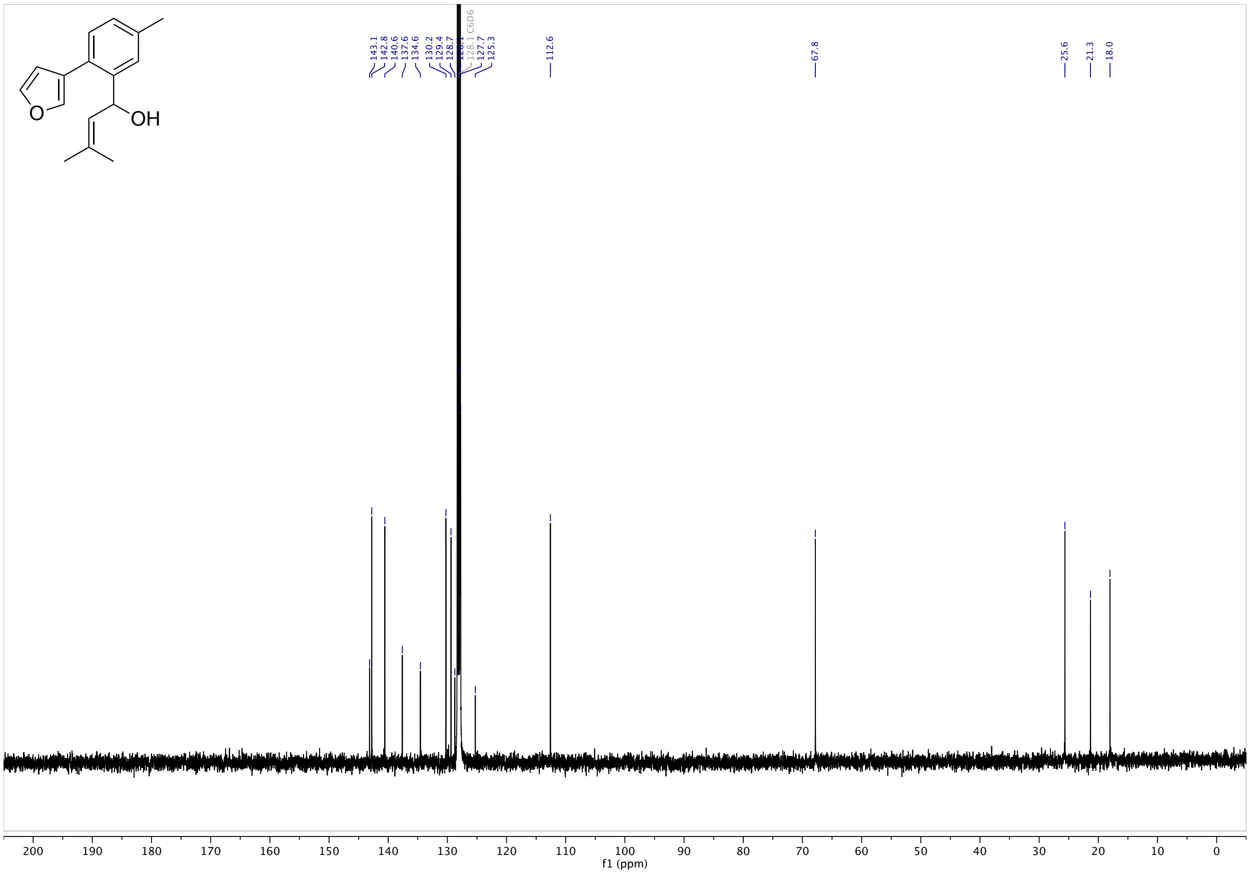

^{1}H NMR (400 MHz, $CDCl_3$) (7)

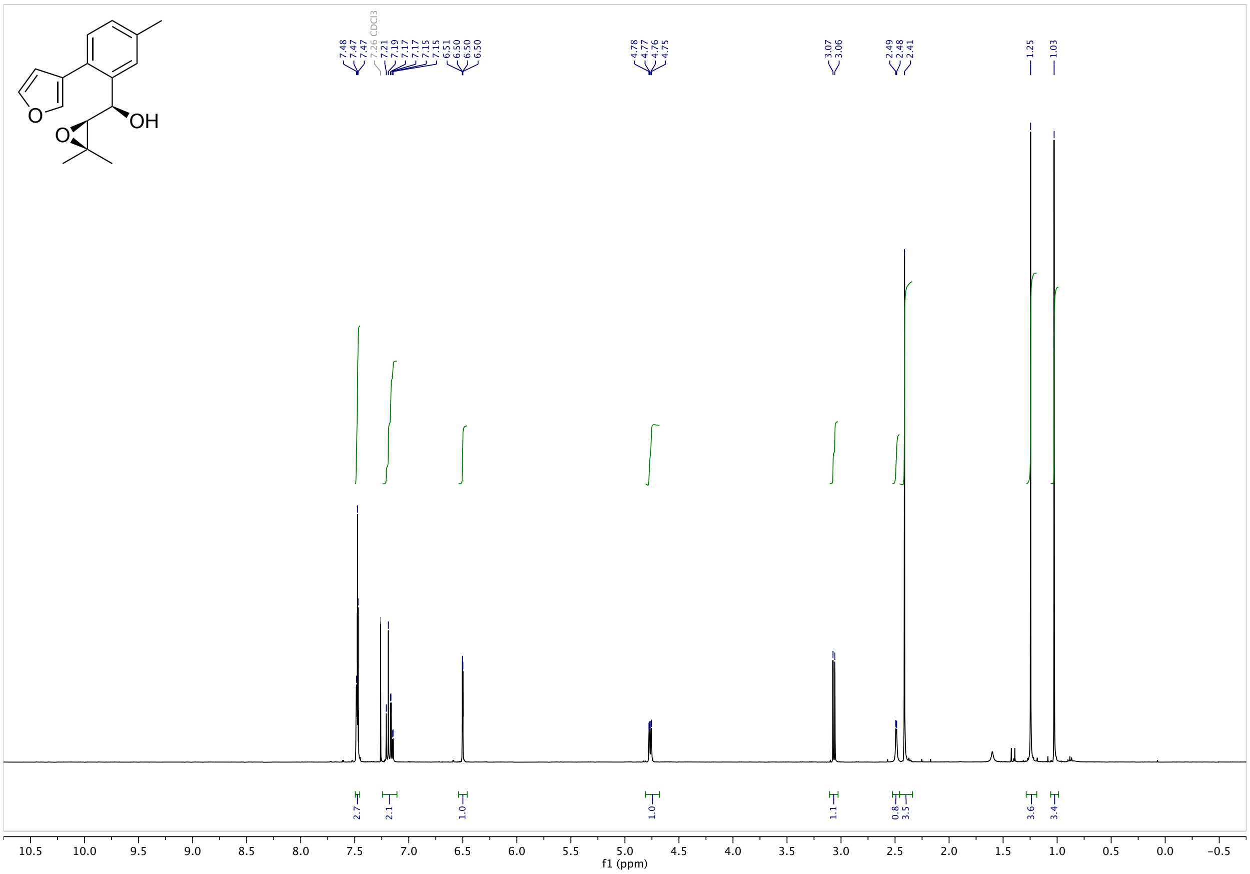

^{13}C NMR (101 MHz, $CDCl_3$) (7)

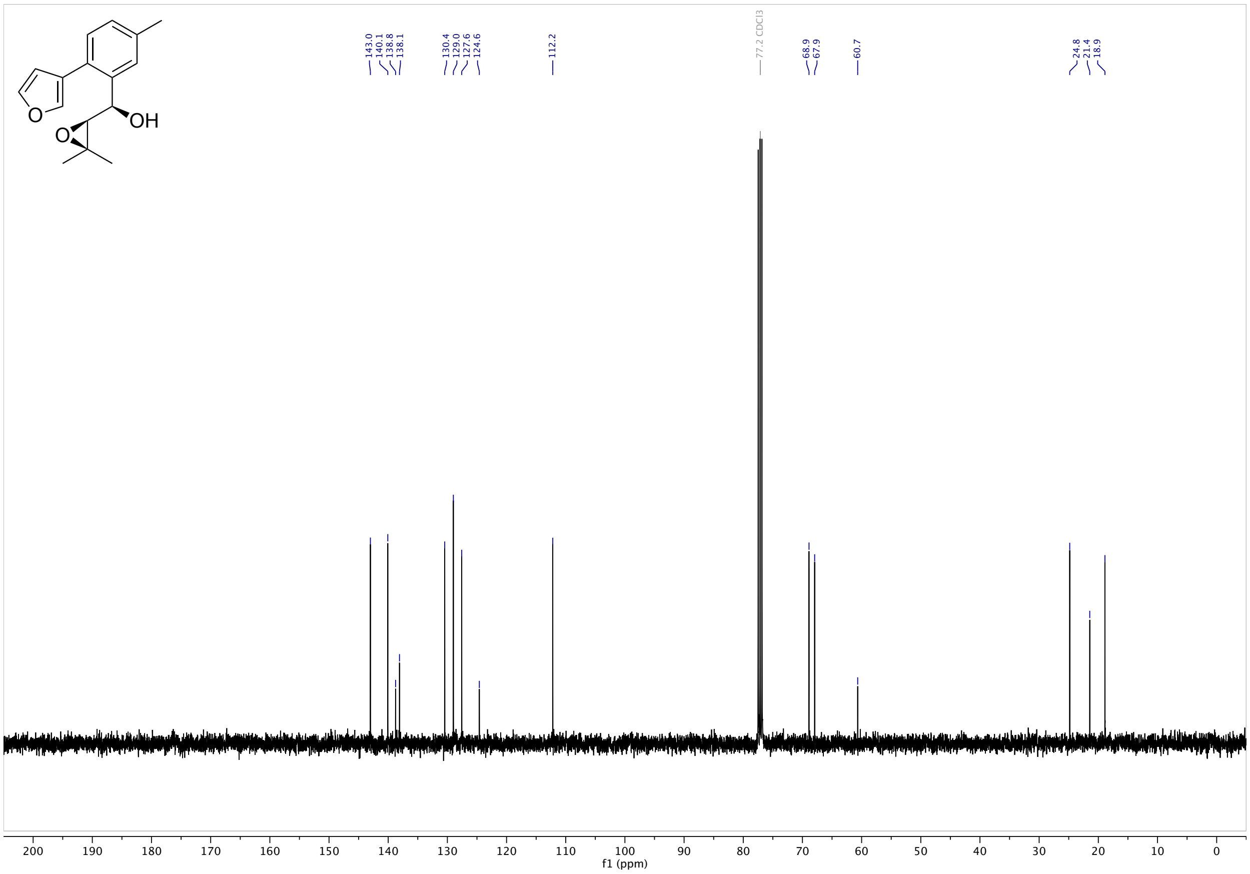

^{1}H NMR (400 MHz, $CDCl_3$) (8)

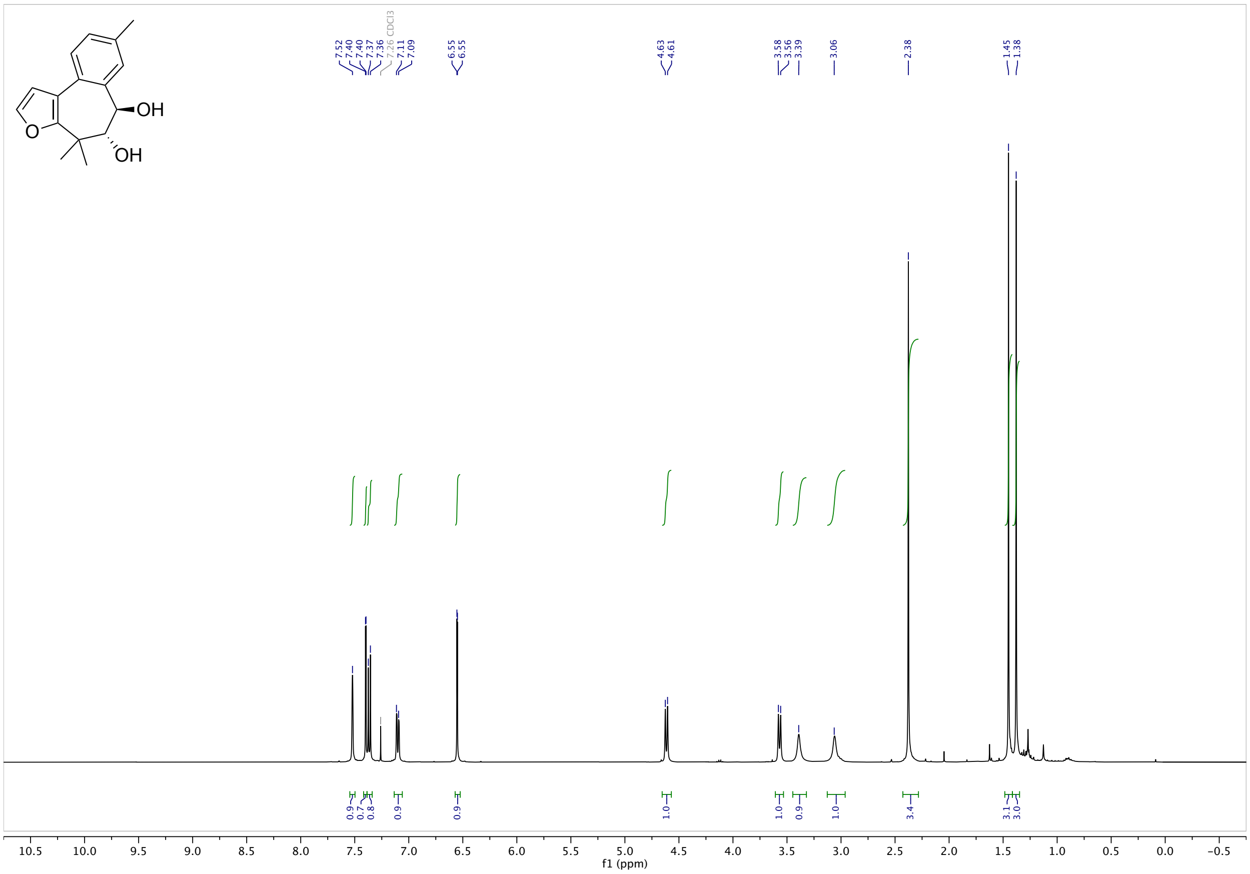

^{13}C NMR (101 MHz, $CDCl_3$) (8)

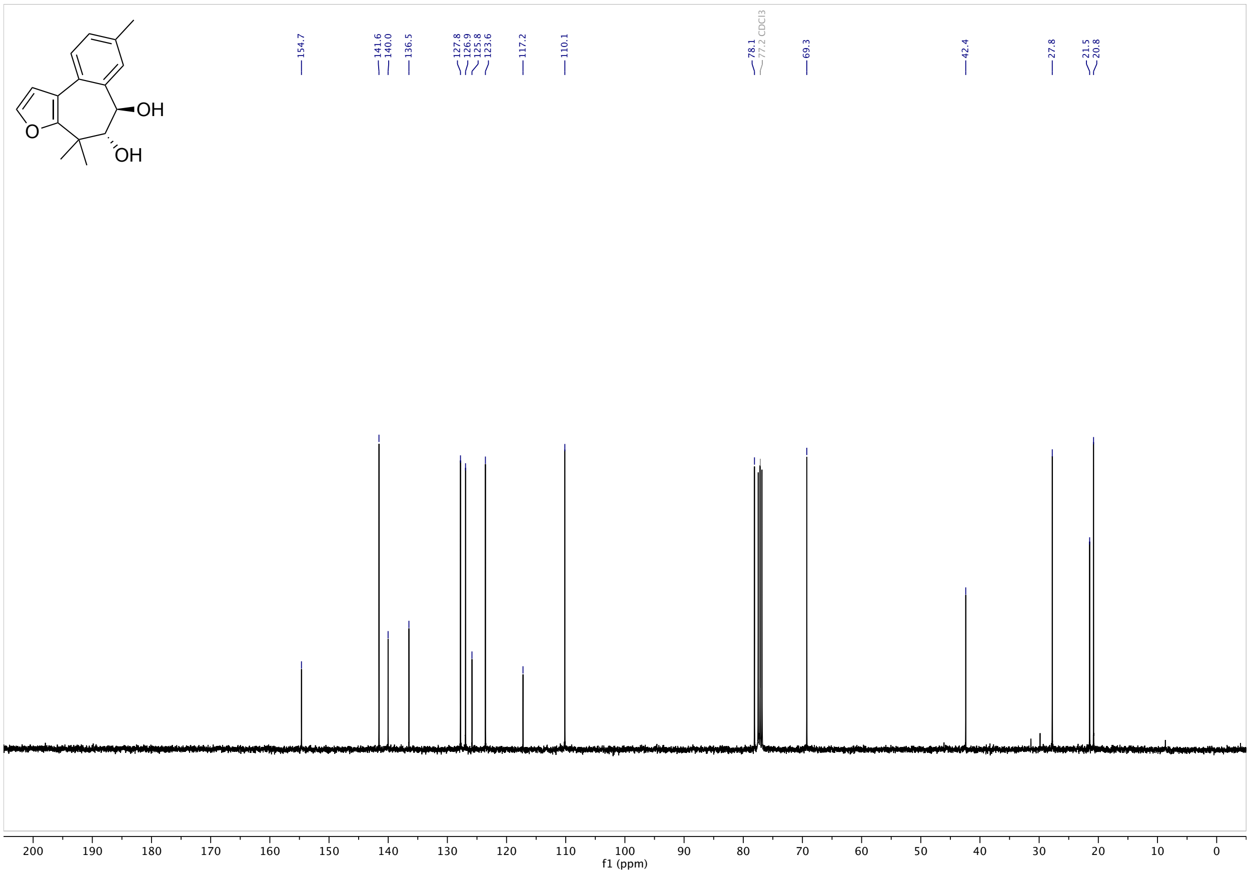

^{1}H NMR (400 MHz, CDCl$_3$) (9)

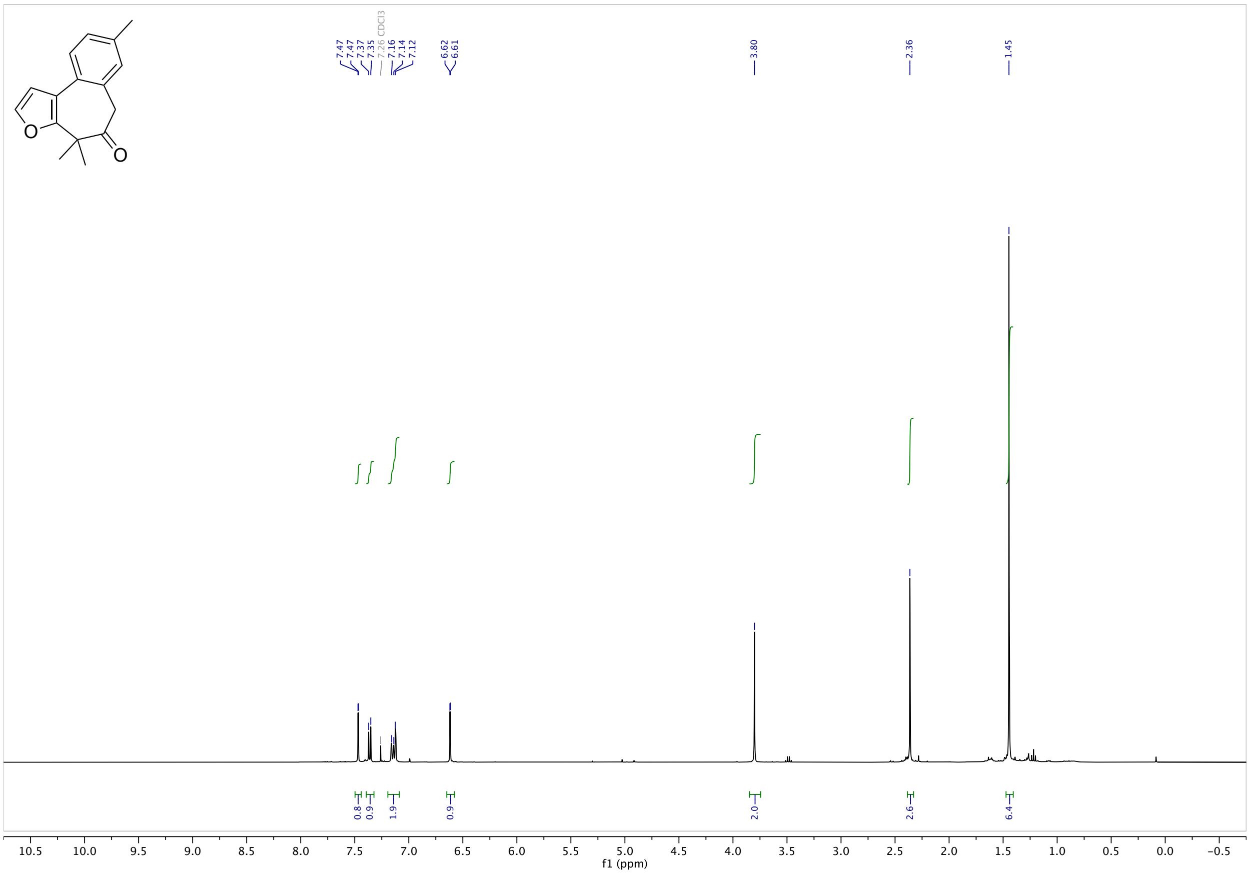

^{13}C NMR (101 MHz, CDCl$_3$) (9)

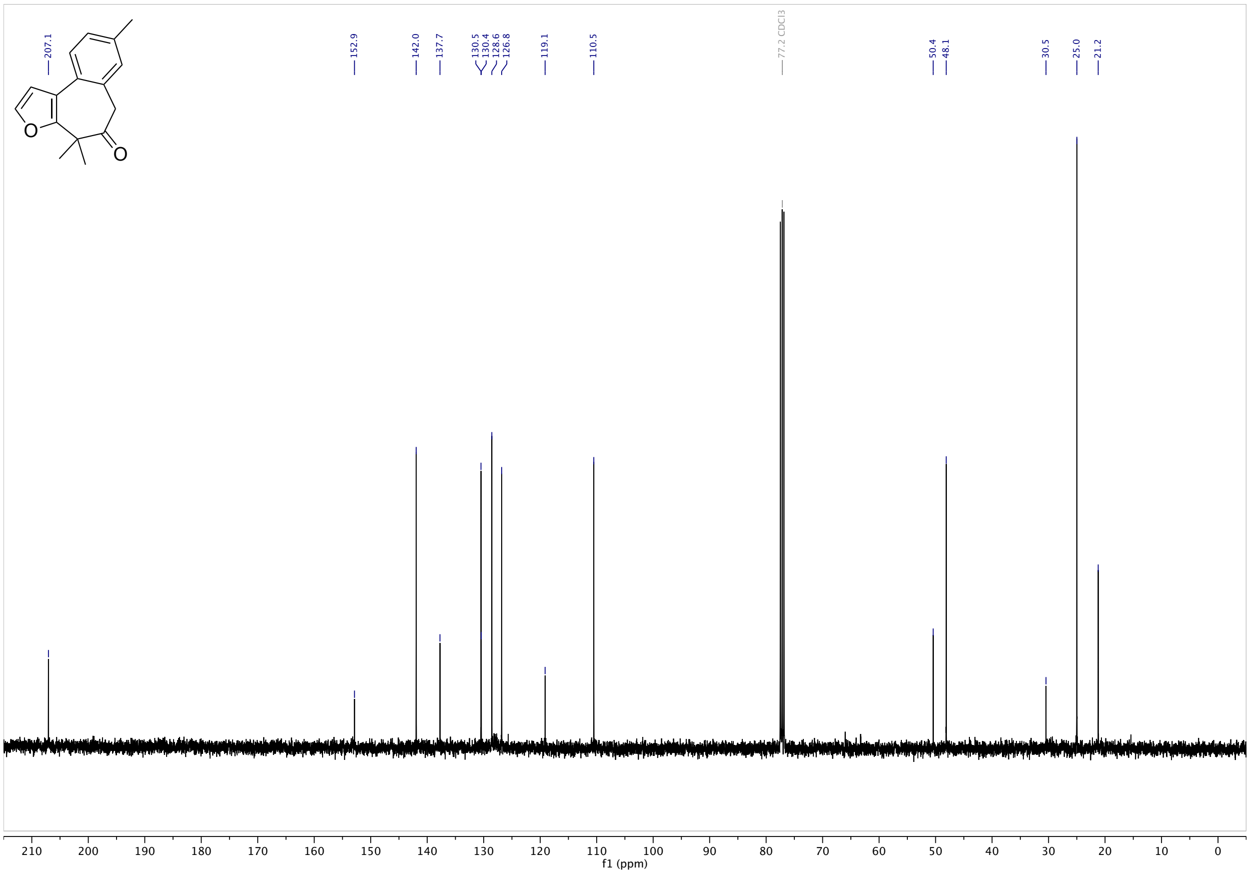

^{1}H NMR (400 MHz, $CDCl_3$)   (10)

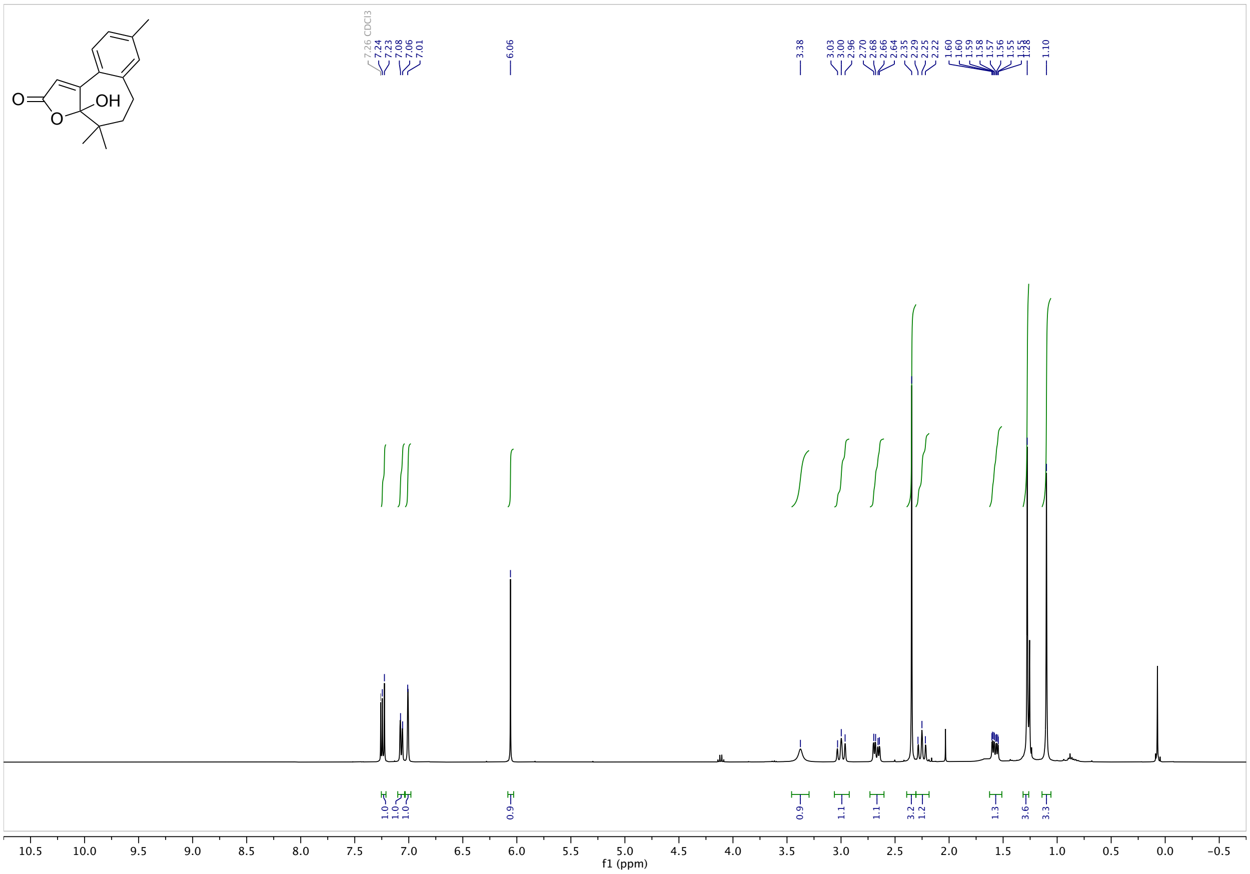

^{13}C NMR (101 MHz, $CDCl_3$)   (10)

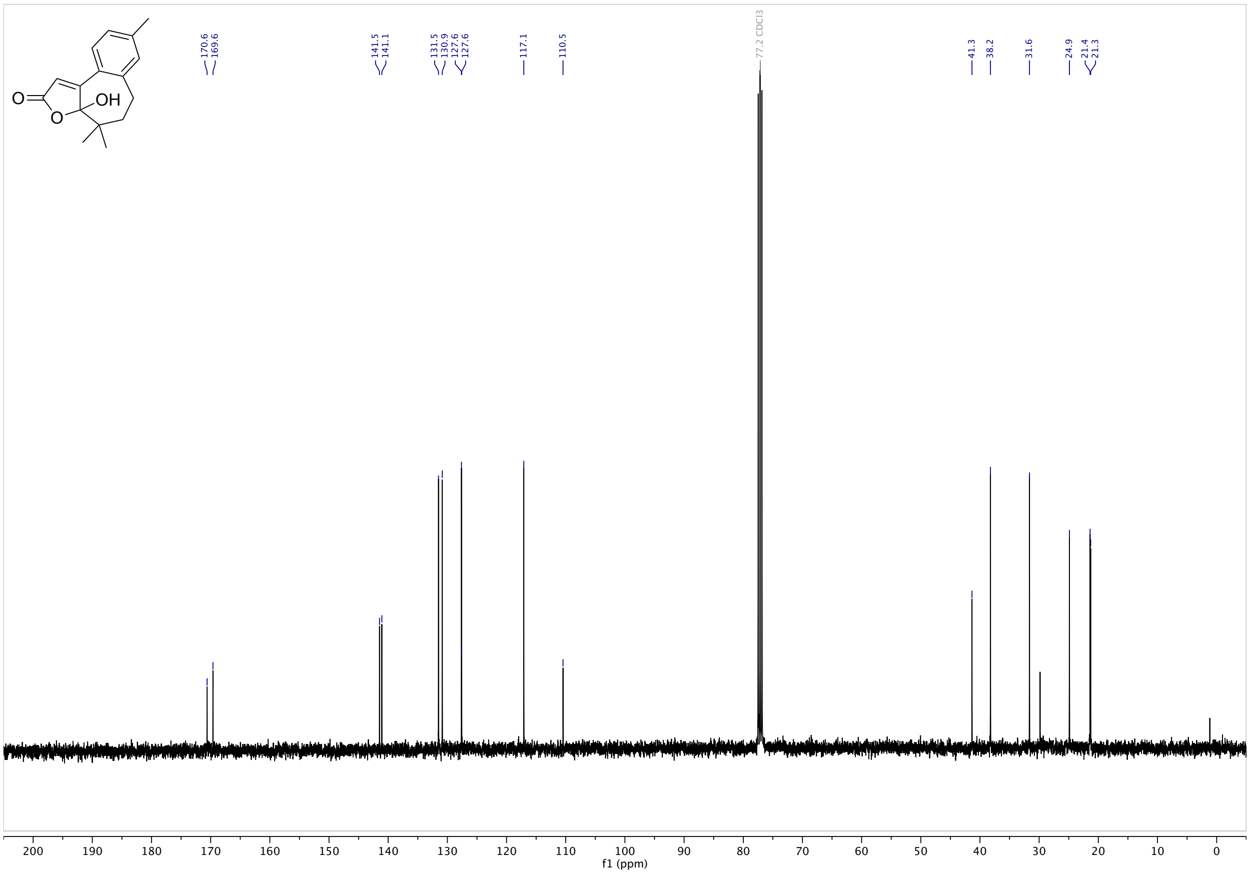

^{1}H NMR (400 MHz, $CDCl_3$) (11)

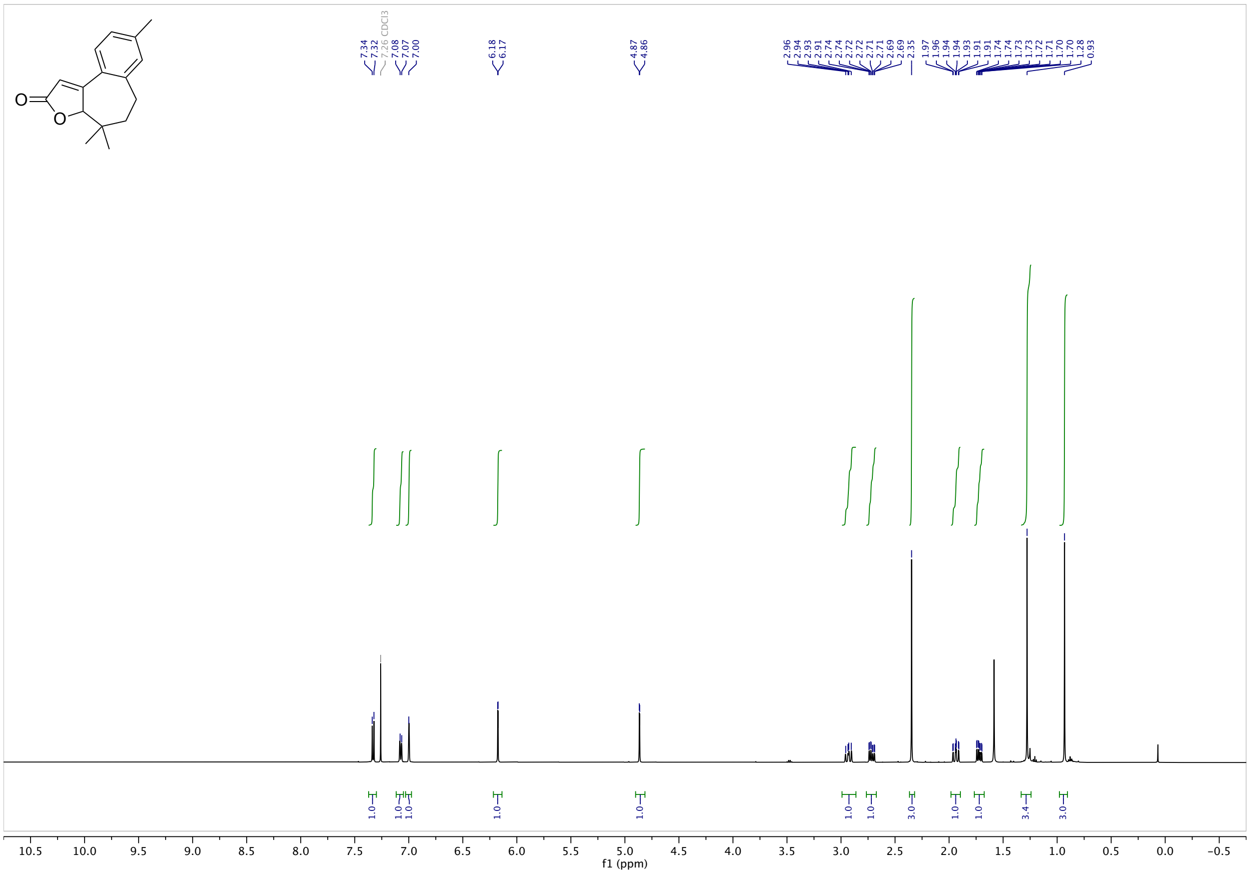

^{13}C NMR (101 MHz, $CDCl_3$) (11)

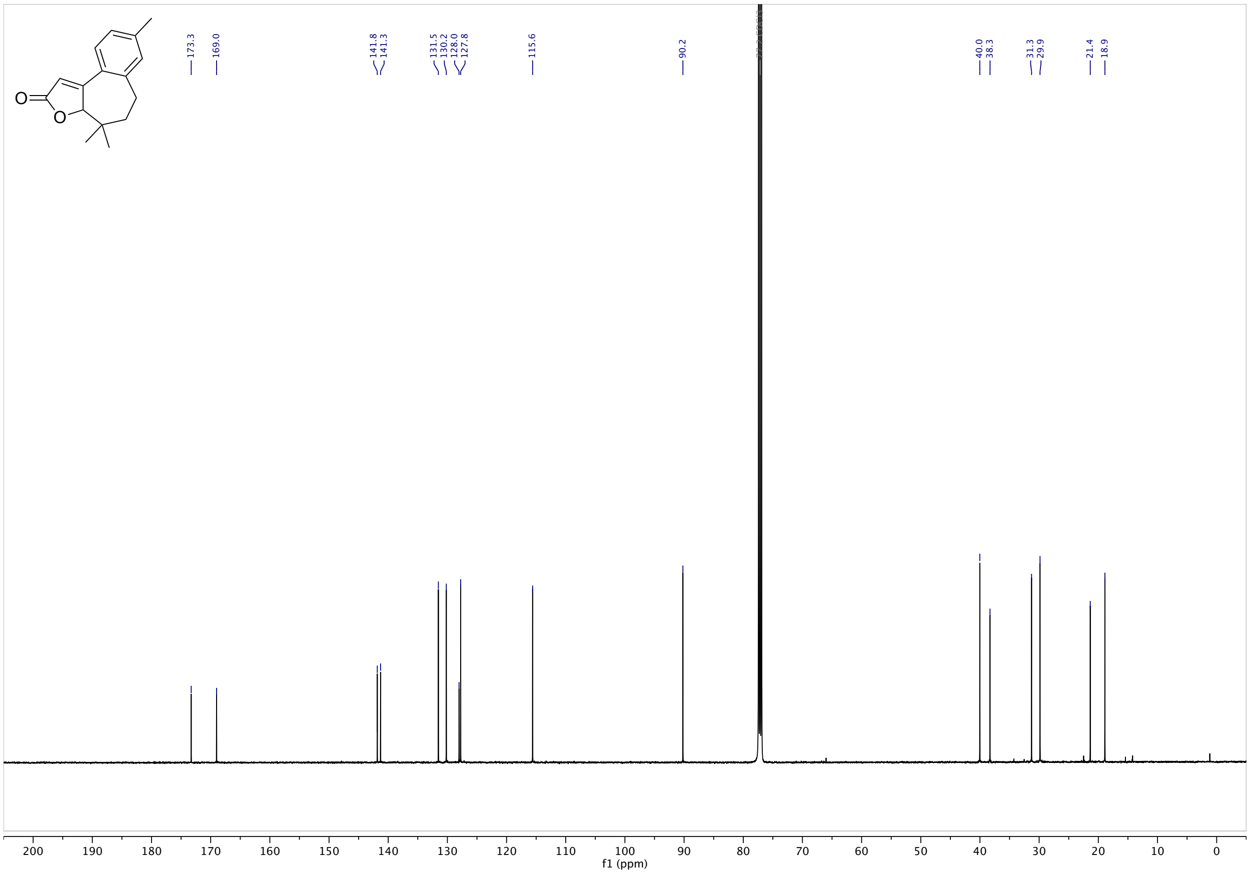

^{1}H NMR (400 MHz, $CDCl_3$) (13)

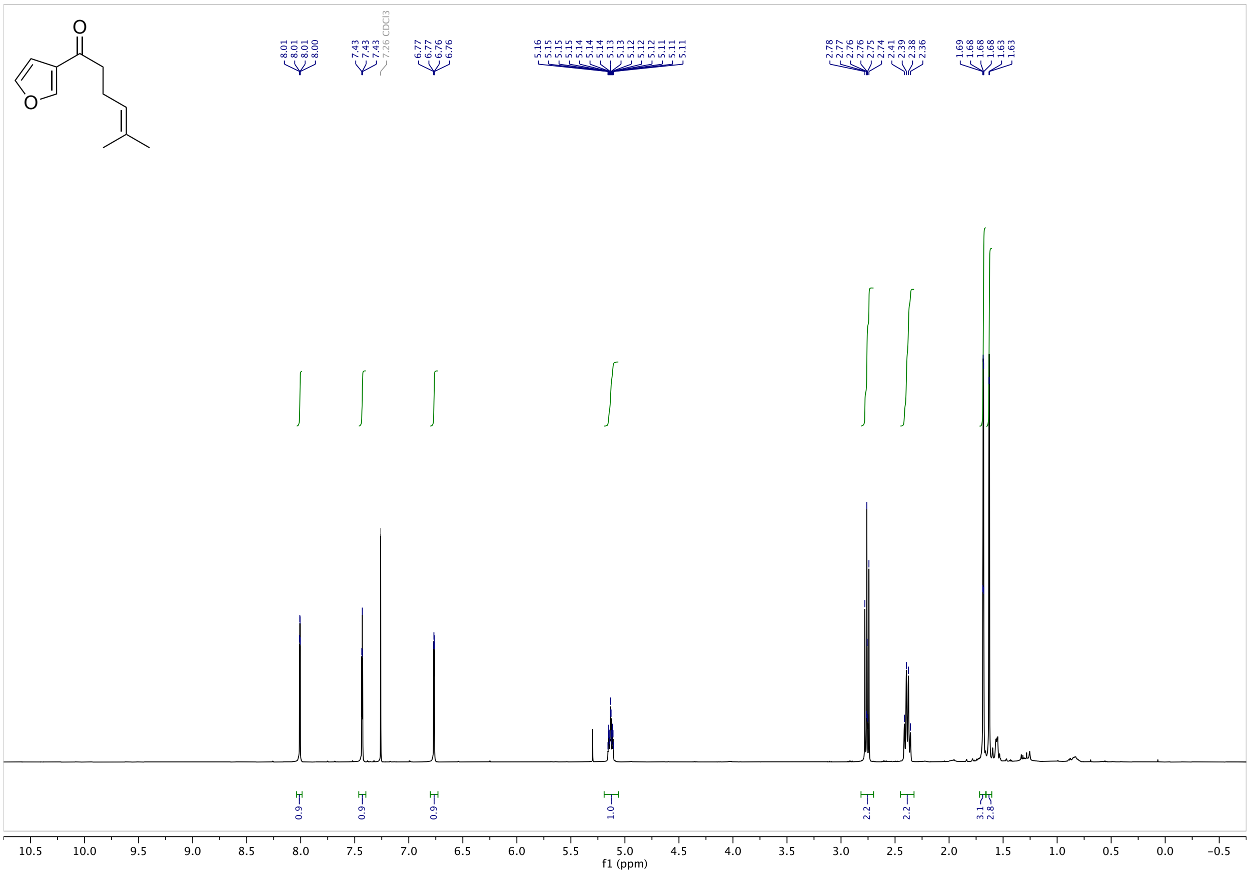

^{13}C NMR (101 MHz, $CDCl_3$) (13)

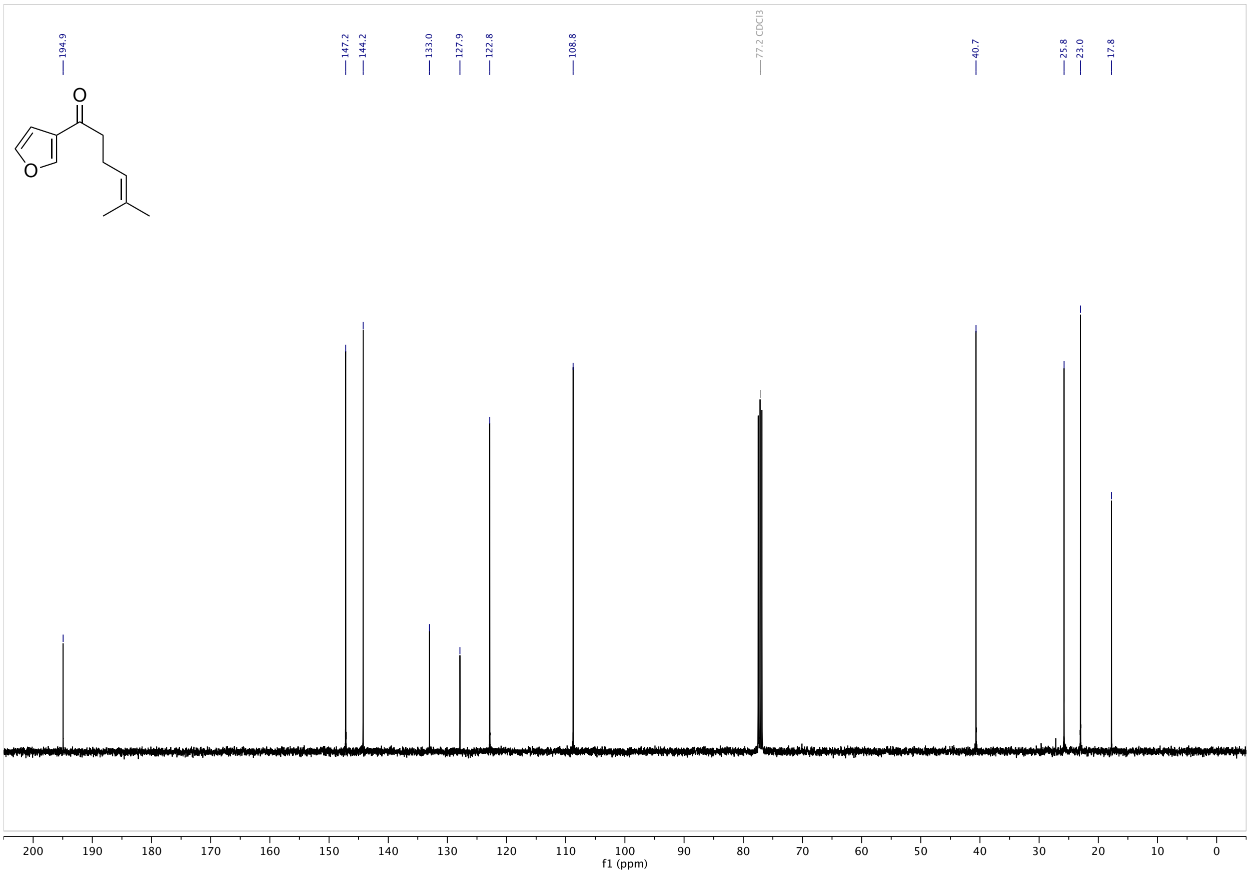

^{1}H NMR (400 MHz, C_6D_6) (14)

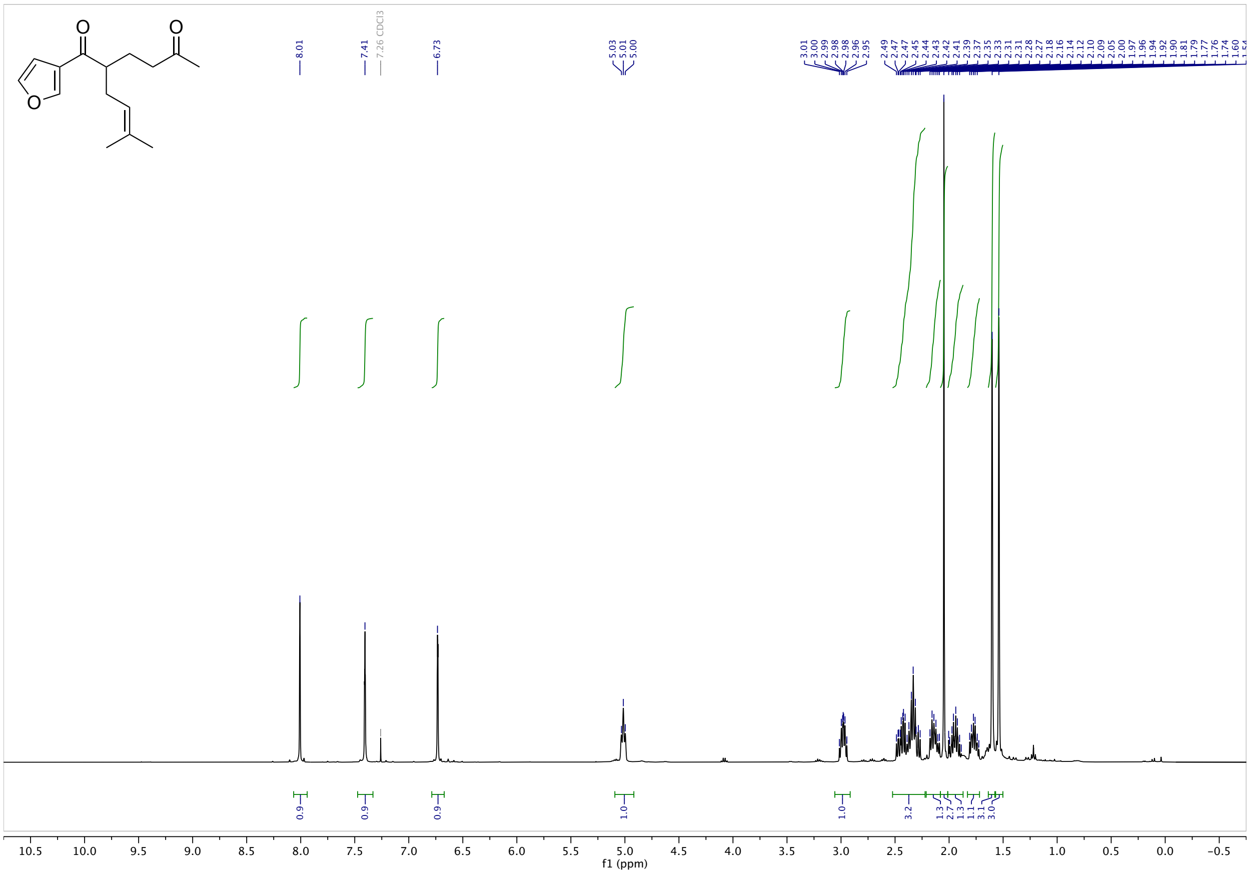

^{13}C NMR (101 MHz, C_6D_6) (14)

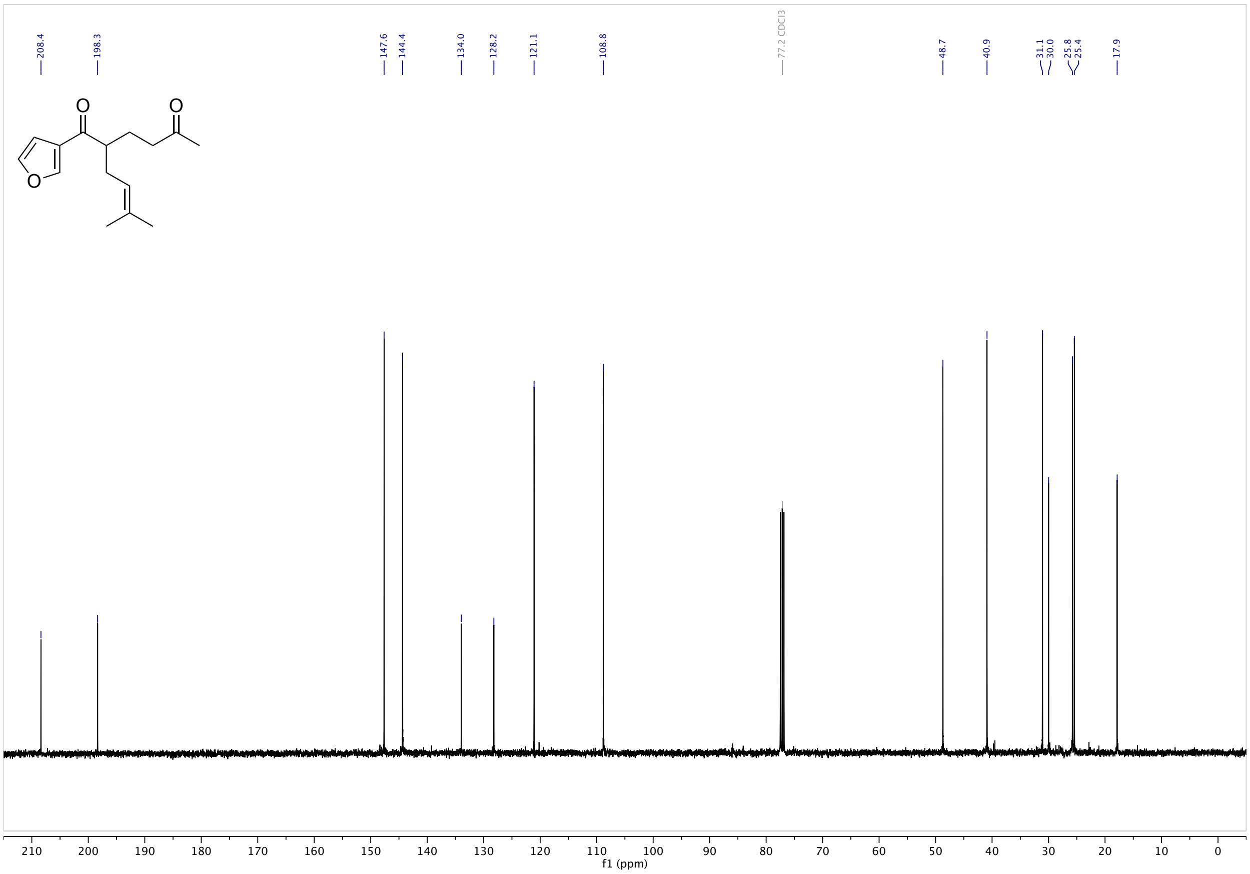

^{1}H NMR (400 MHz, CDCl$_3$) (15)

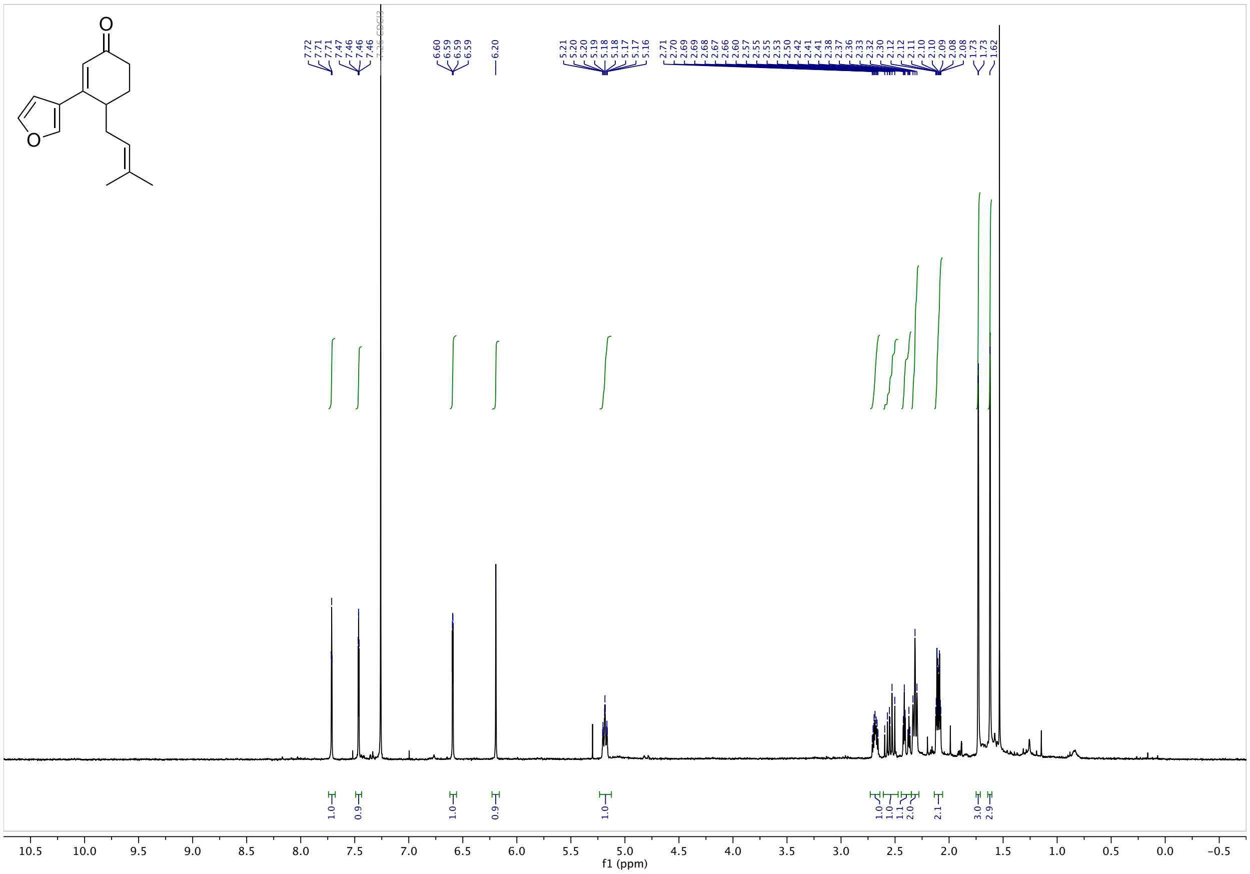

^{13}C NMR (101 MHz, CDCl$_3$) (15)

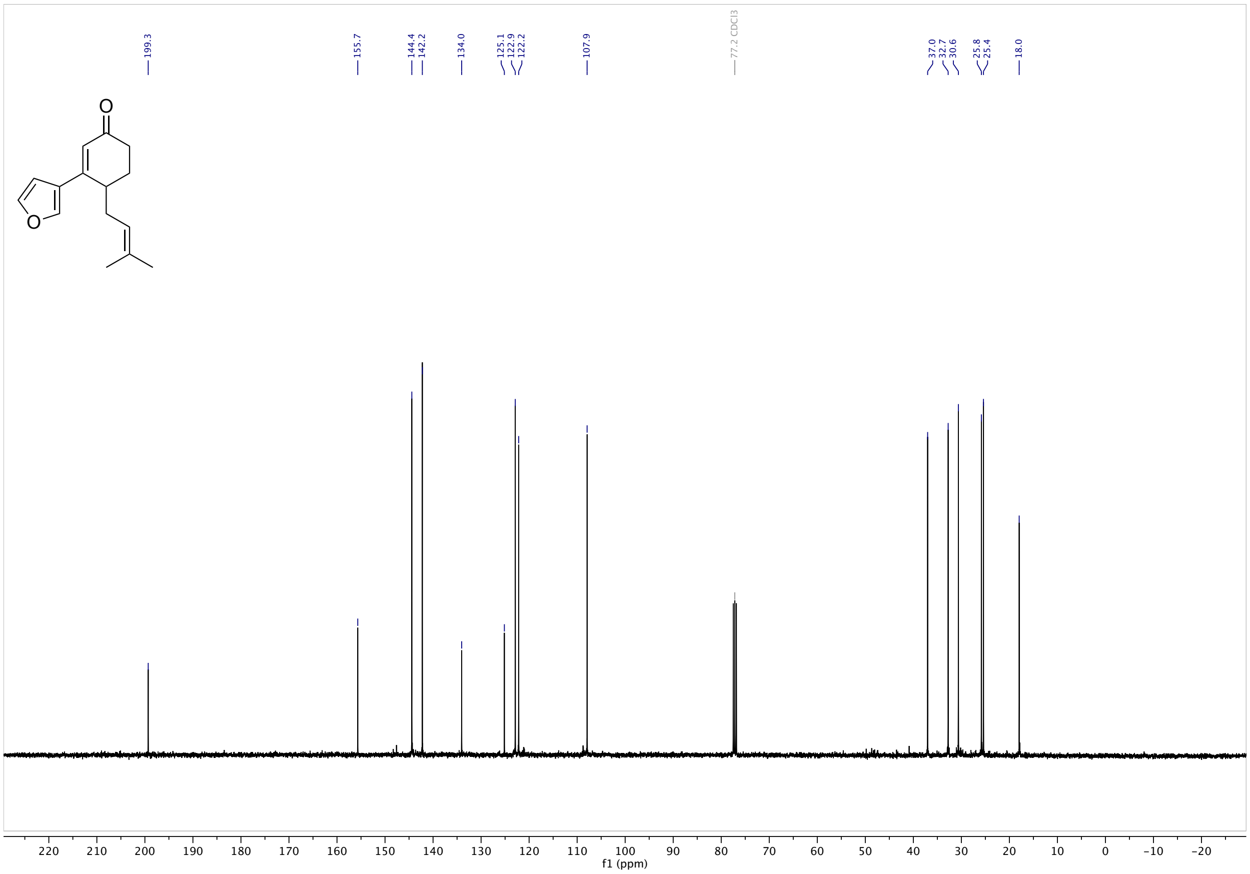

^{1}H NMR (400 MHz, CDCl$_3$) (16)

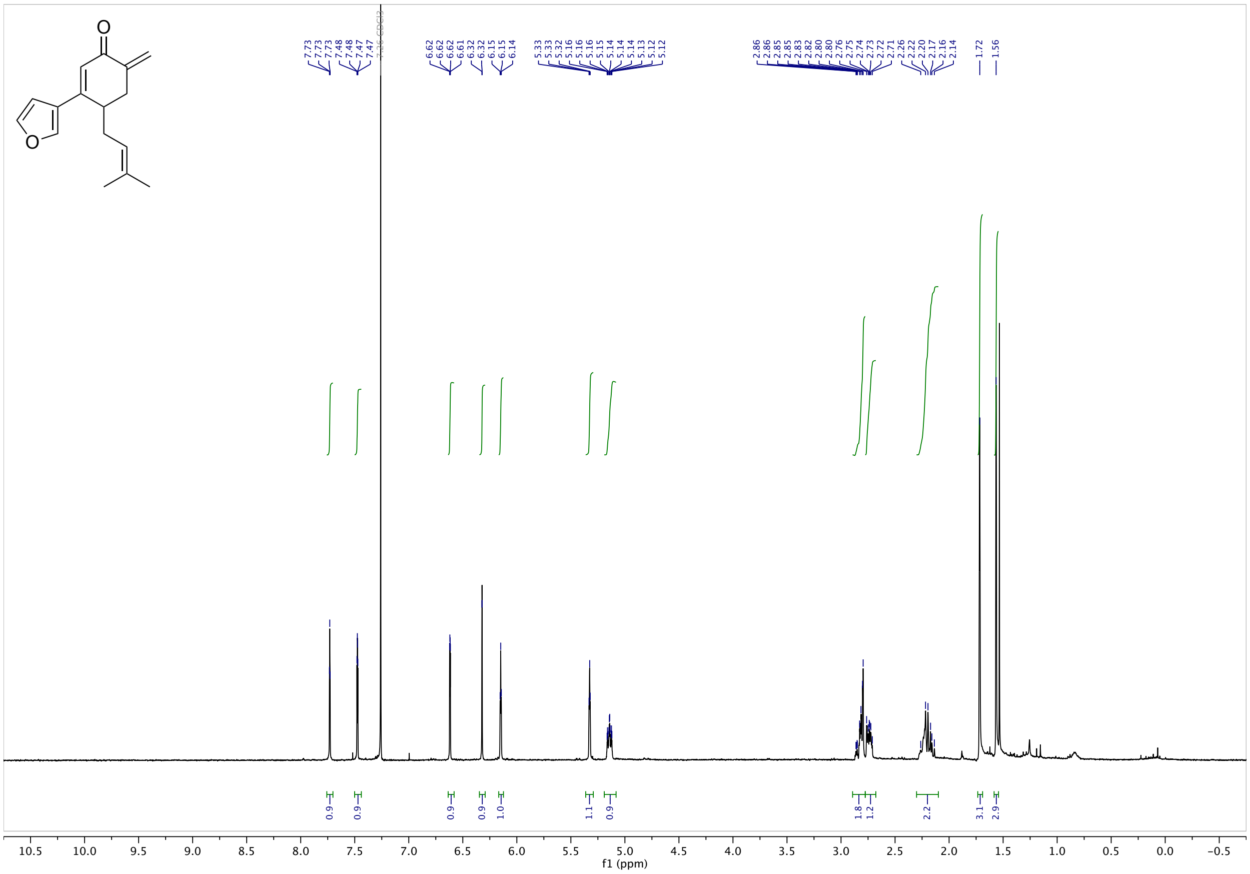

^{13}C NMR (101 MHz, CDCl$_3$) (16)

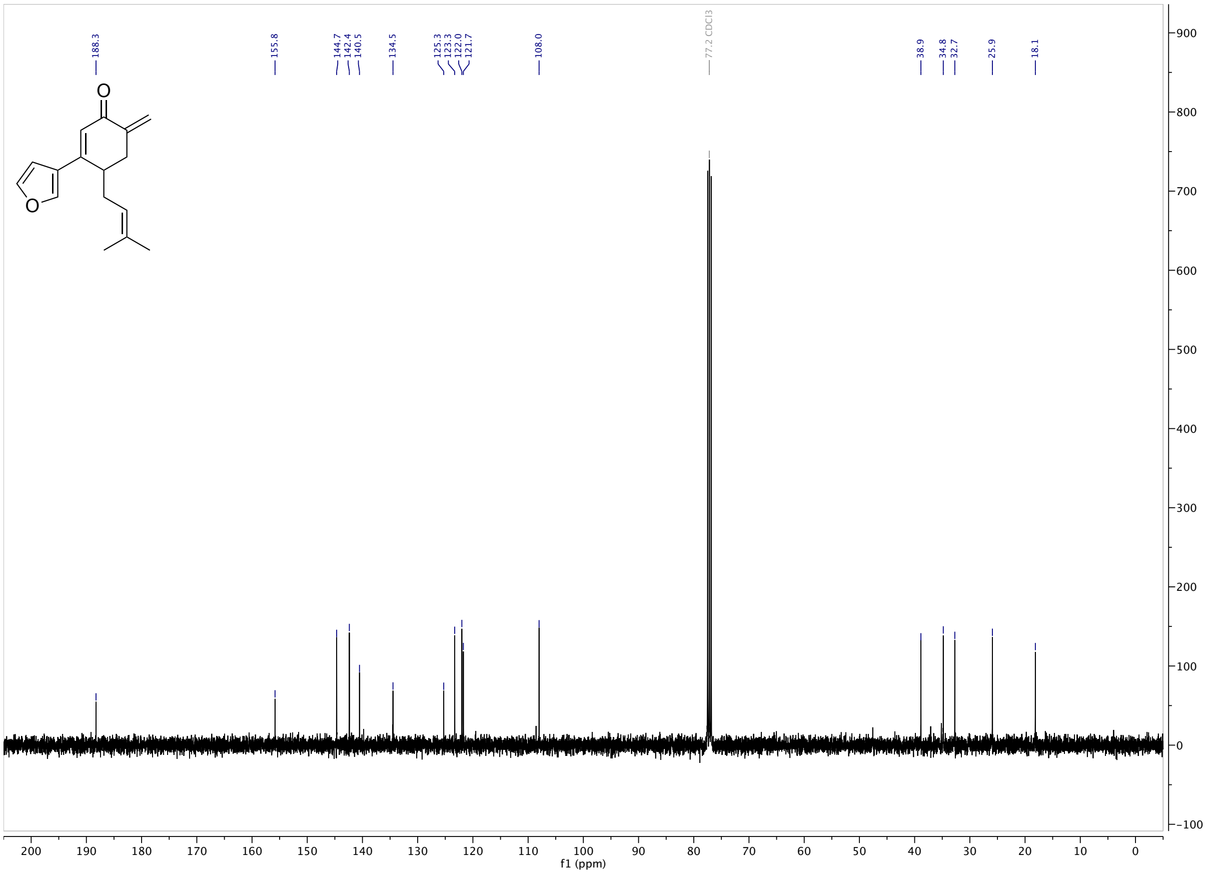

^{1}H NMR (400 MHz, $CDCl_3$) (17)

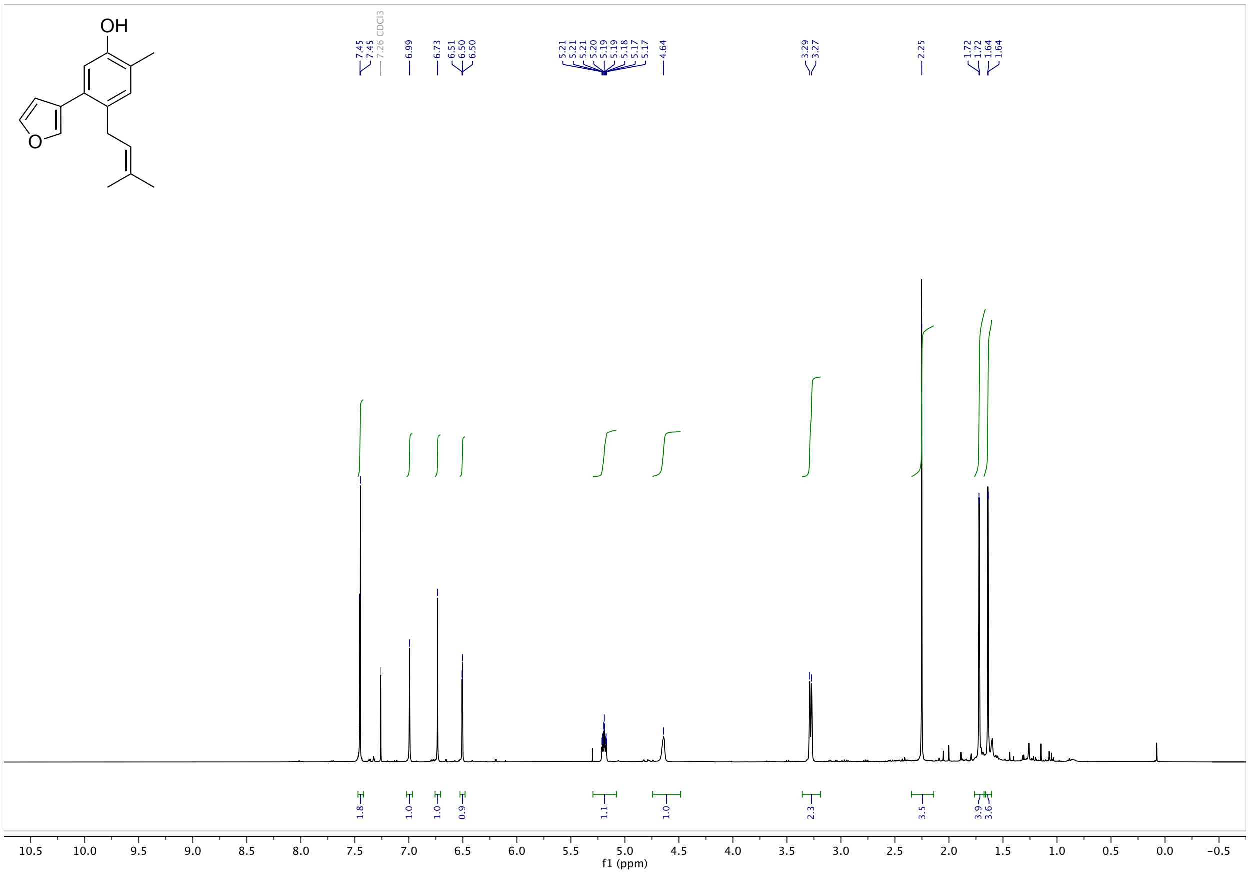

^{13}C NMR (101 MHz, $CDCl_3$) (17)

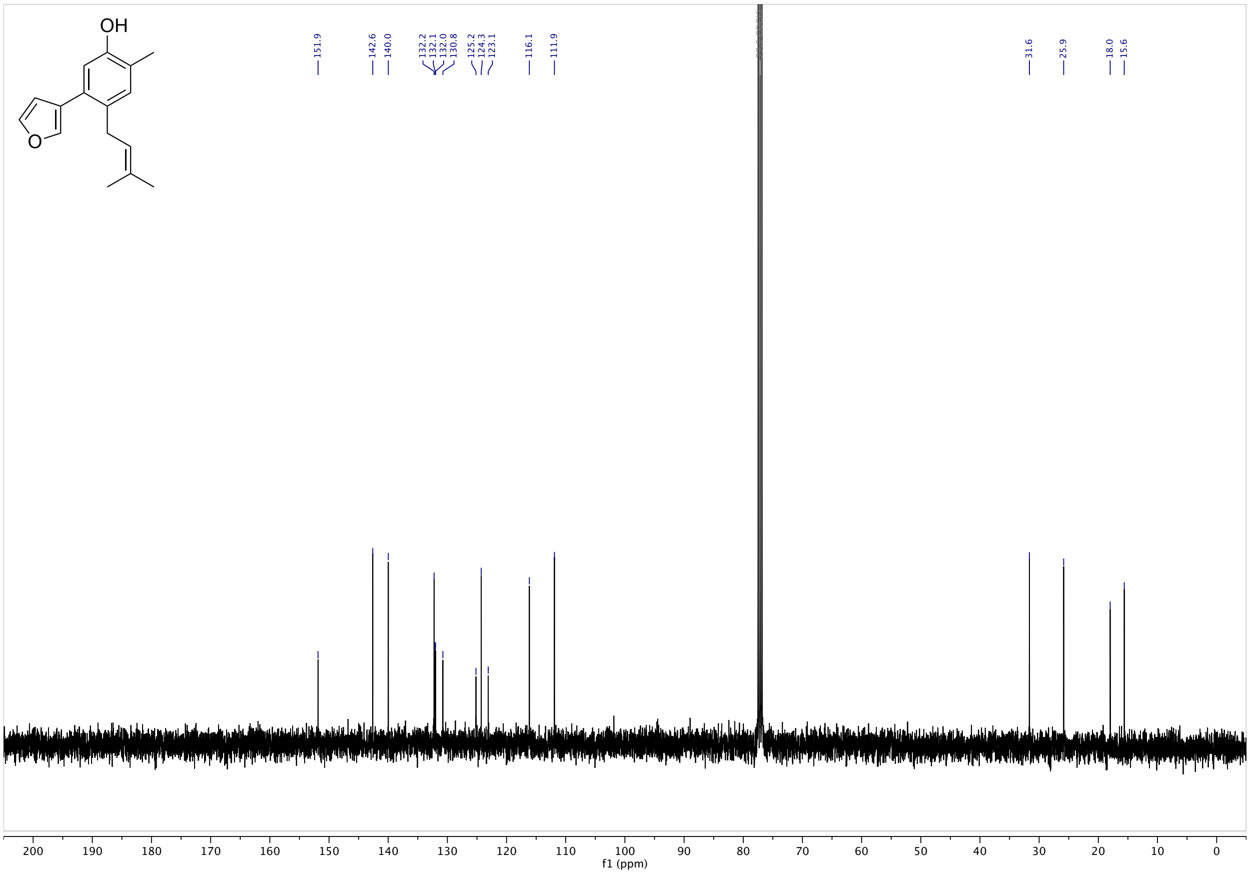

^{1}H NMR (400 MHz, $CDCl_3$)   (18)

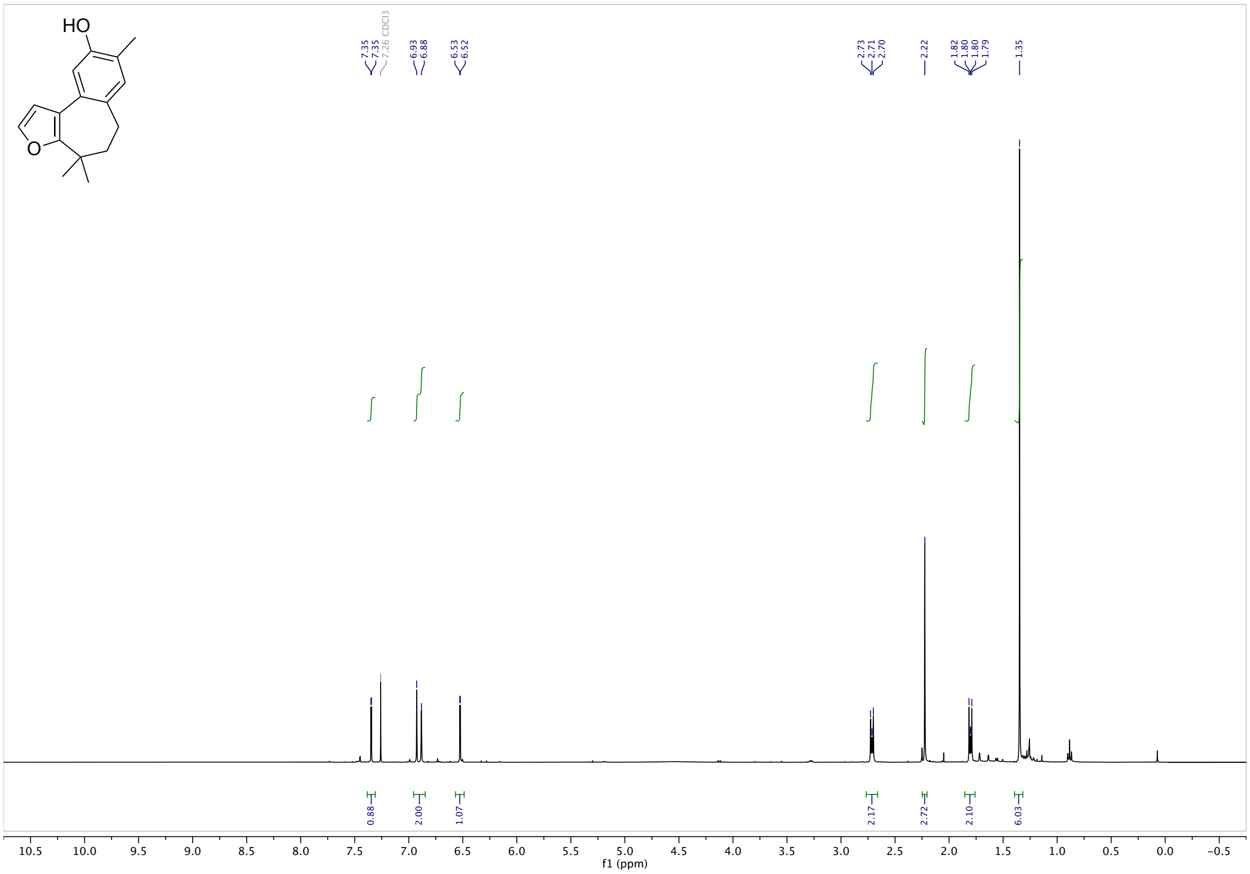

^{13}C NMR (101 MHz, $CDCl_3$)   (18)

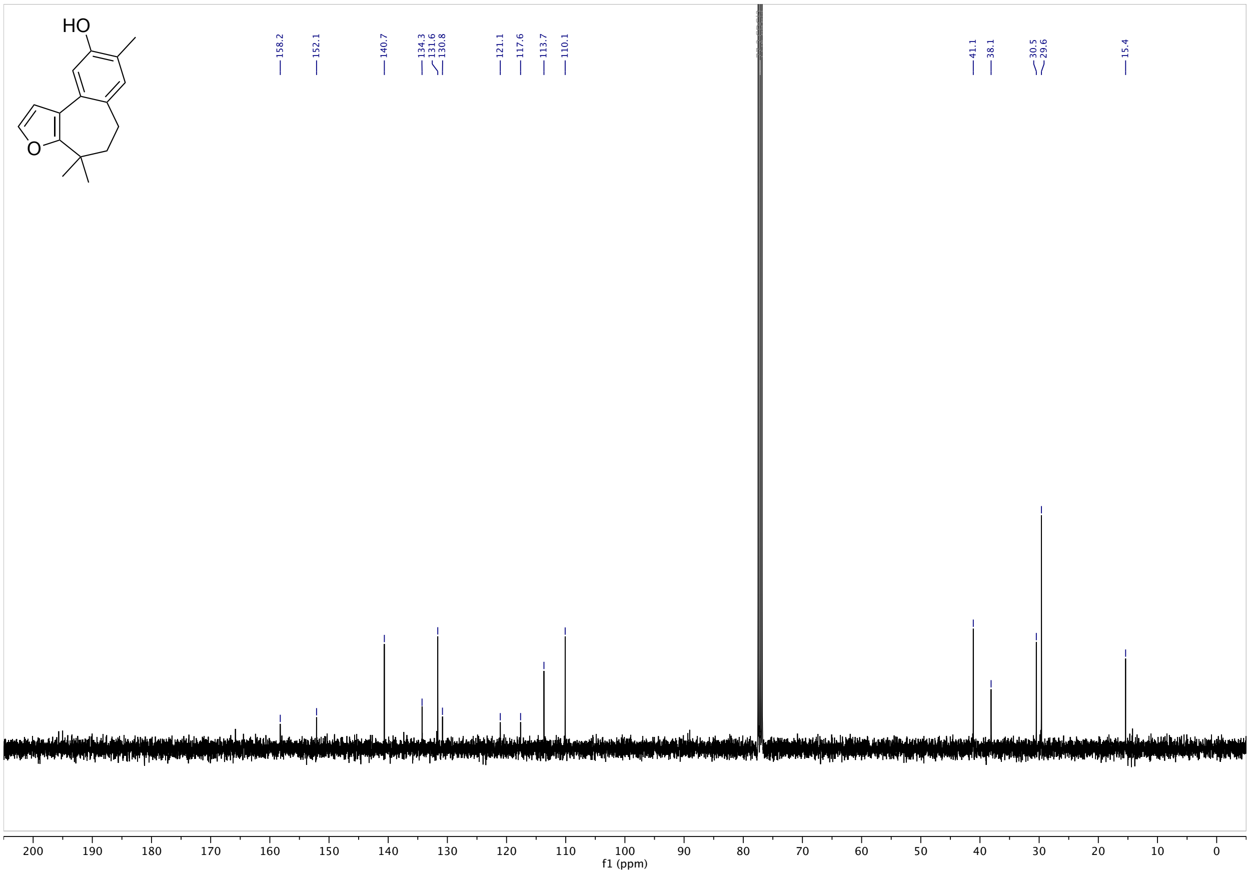

^{1}H NMR (400 MHz, $CDCl_3$) (20)

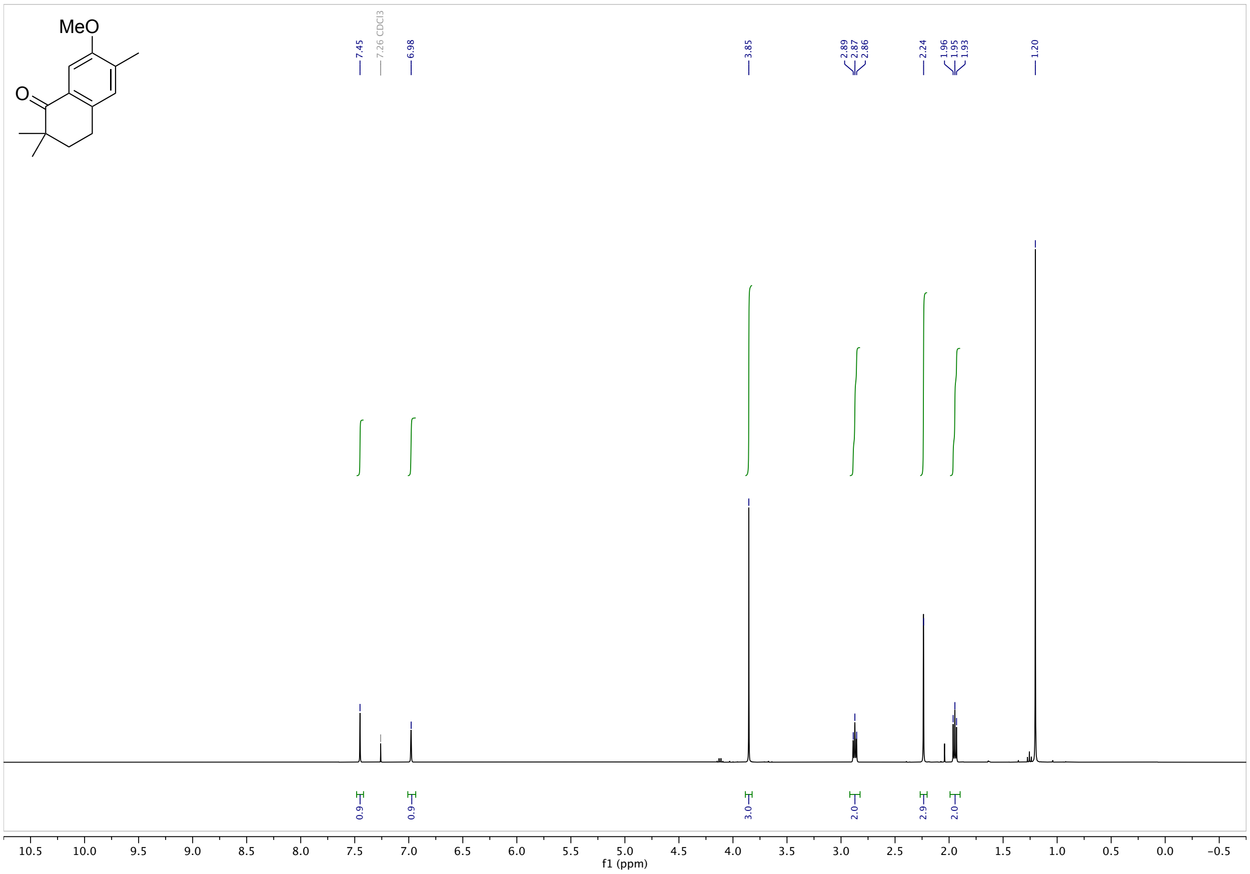

^{13}C NMR (101 MHz, $CDCl_3$) (20)

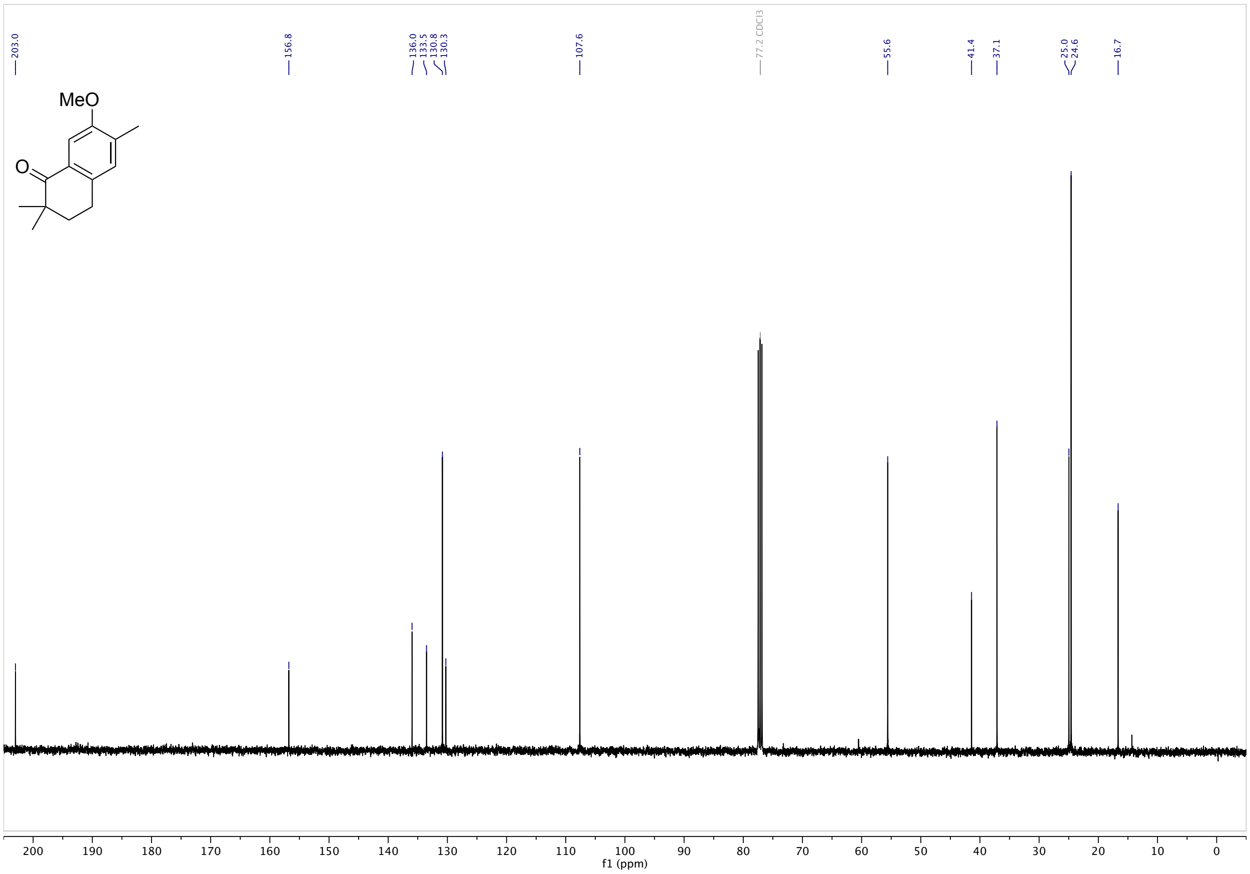

^{1}H NMR (400 MHz, $CDCl_3$) (21)

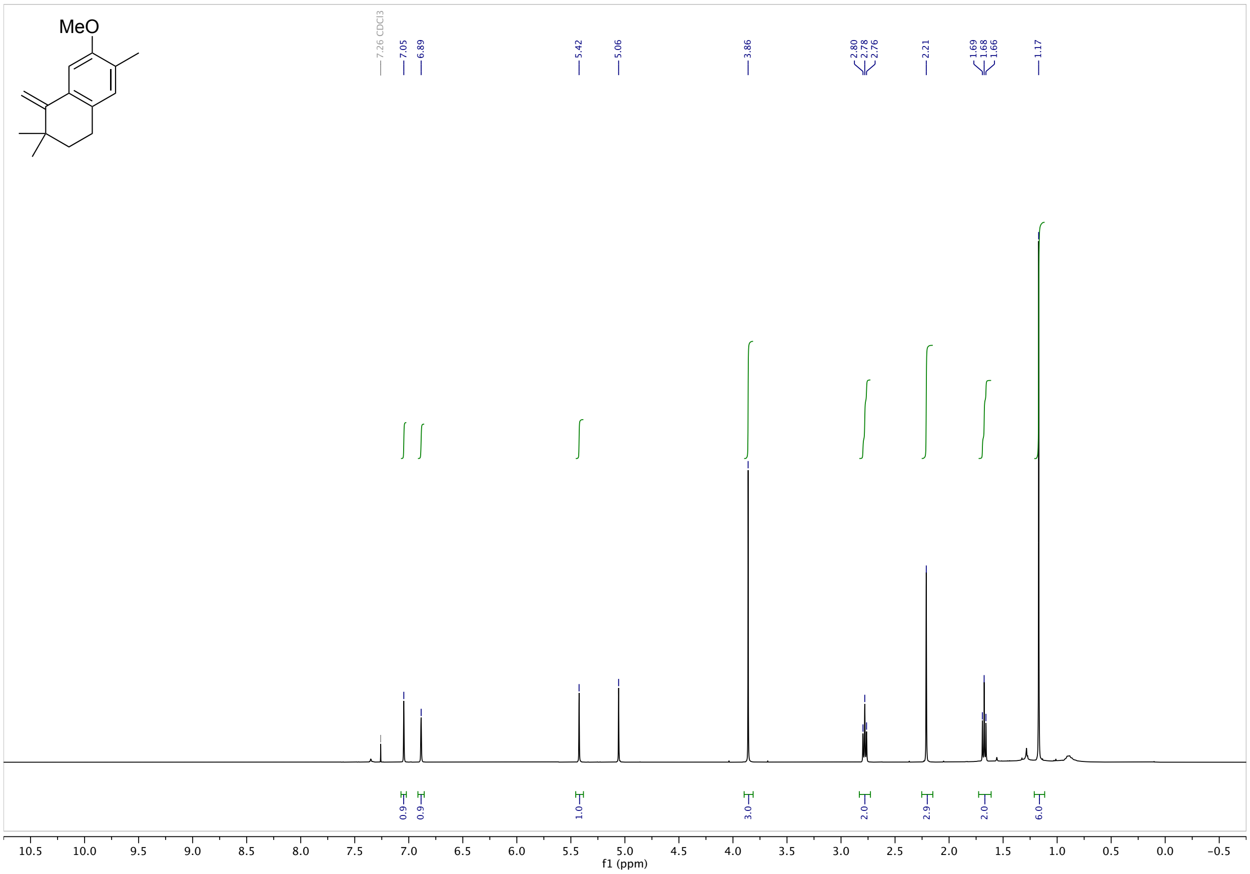

^{13}C NMR (101 MHz, $CDCl_3$) (21)

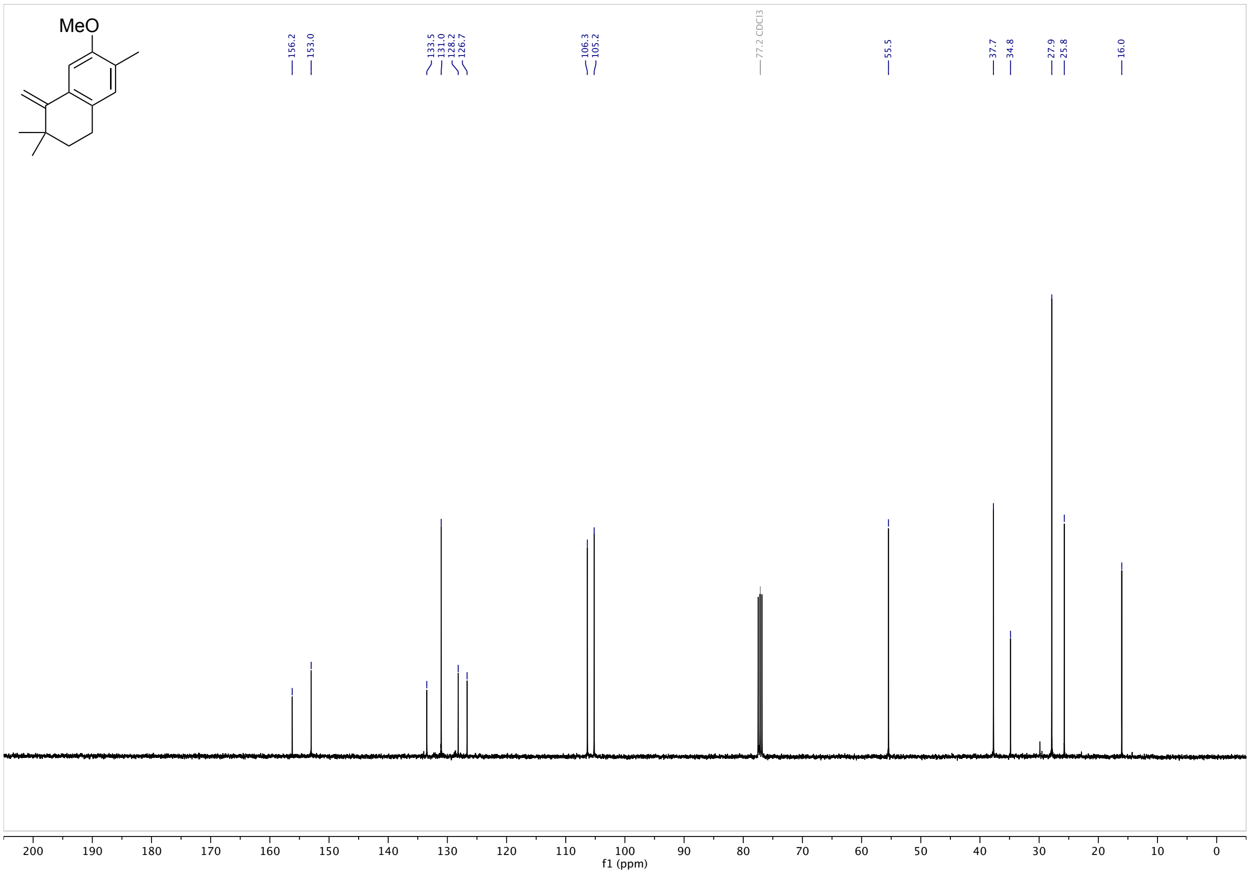

^{1}H NMR (400 MHz, $CDCl_3$) (22)

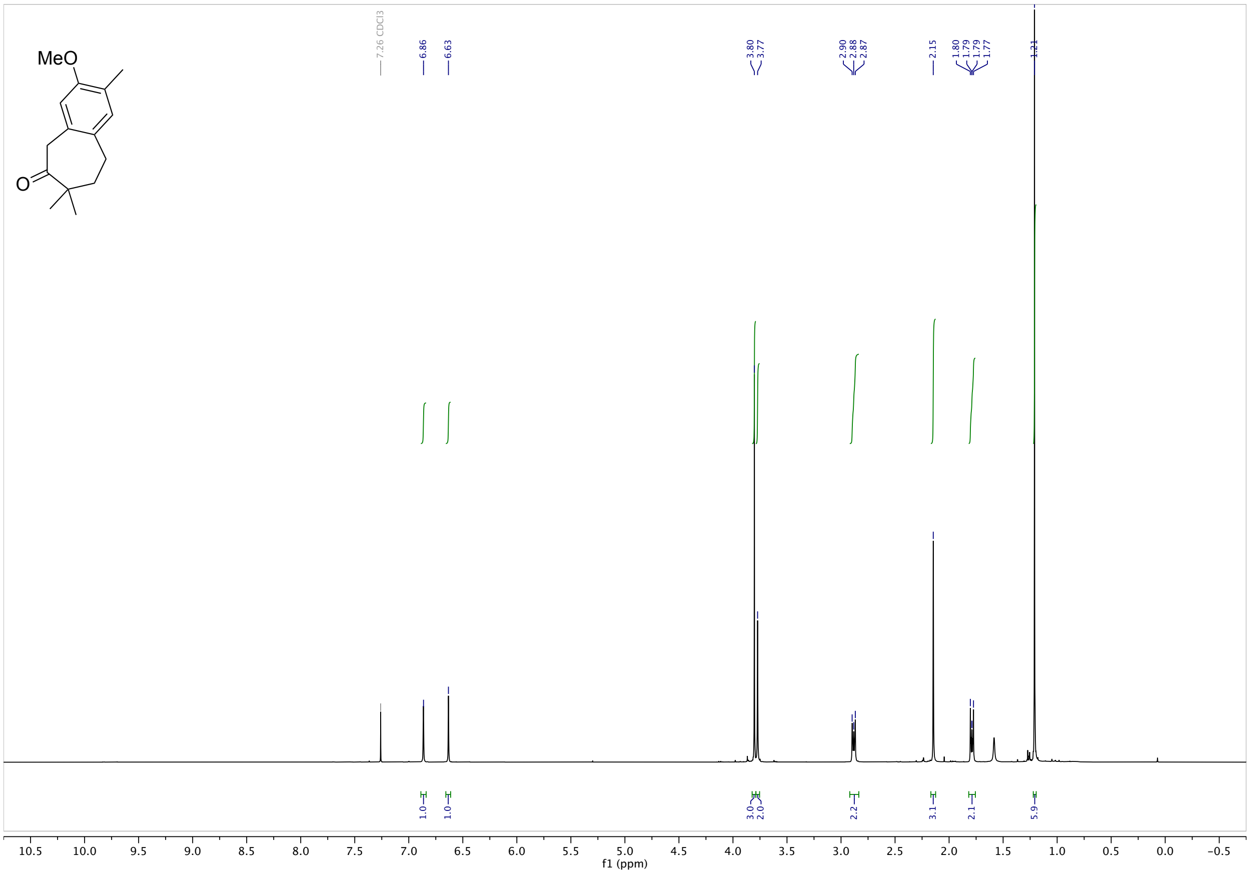

^{13}C NMR (101 MHz, $CDCl_3$) (22)

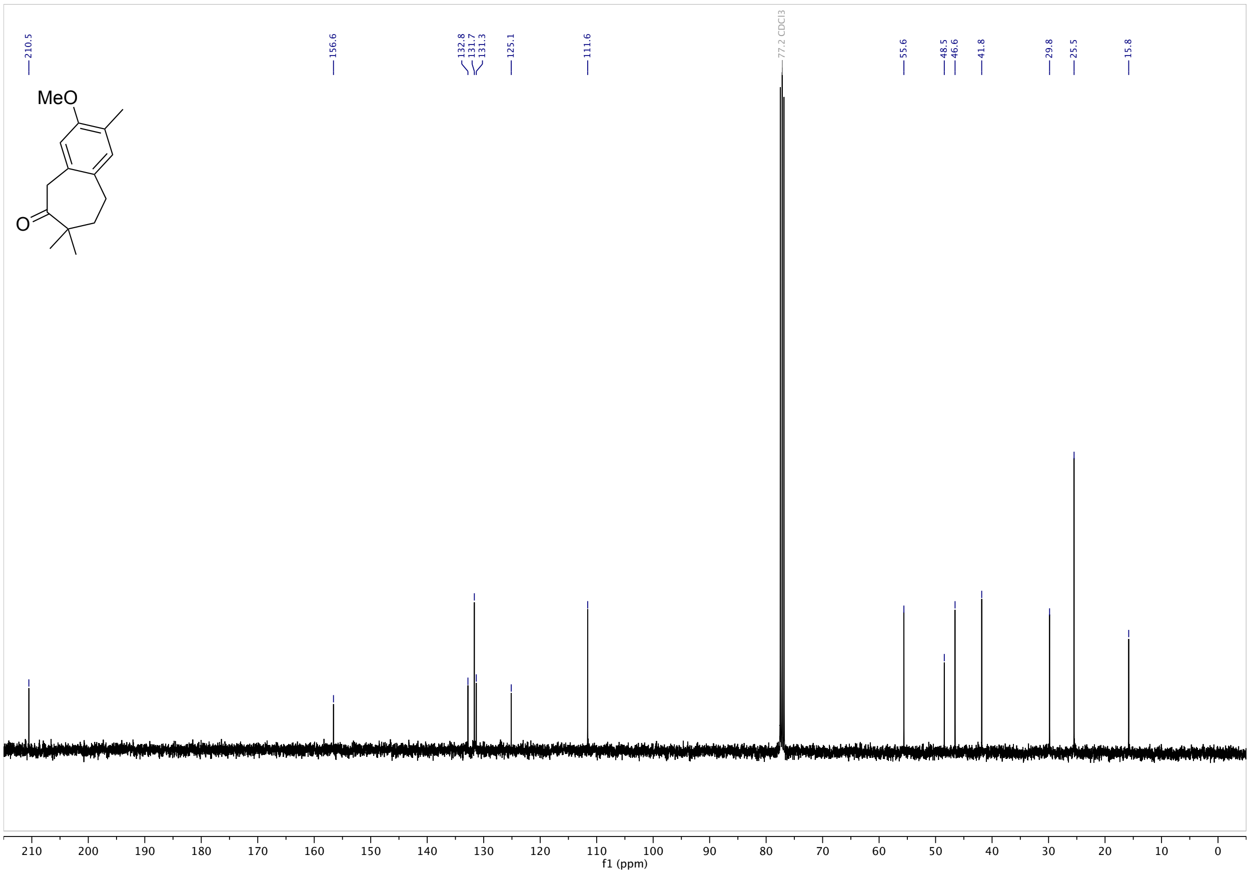

^{1}H NMR (400 MHz, $CDCl_3$) (23)

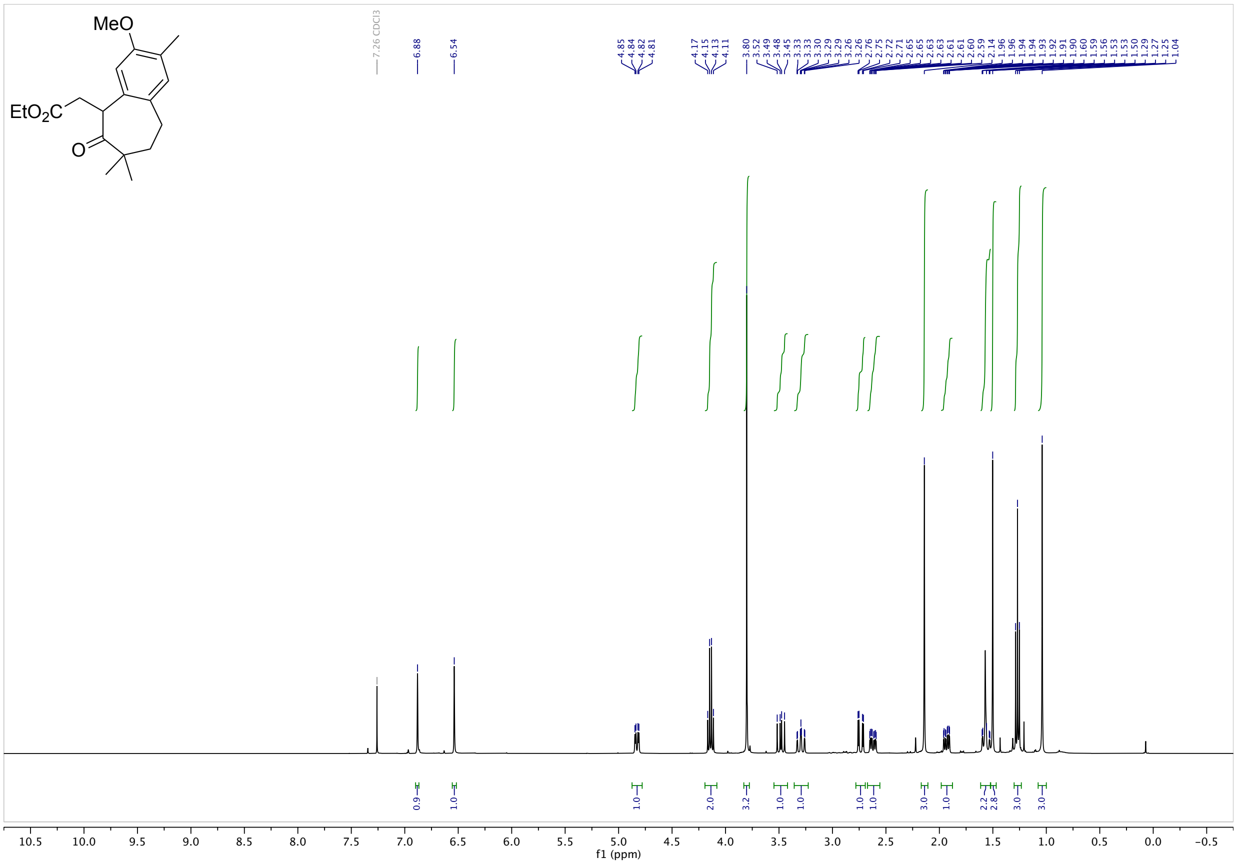

^{13}C NMR (101 MHz, $CDCl_3$) (23)

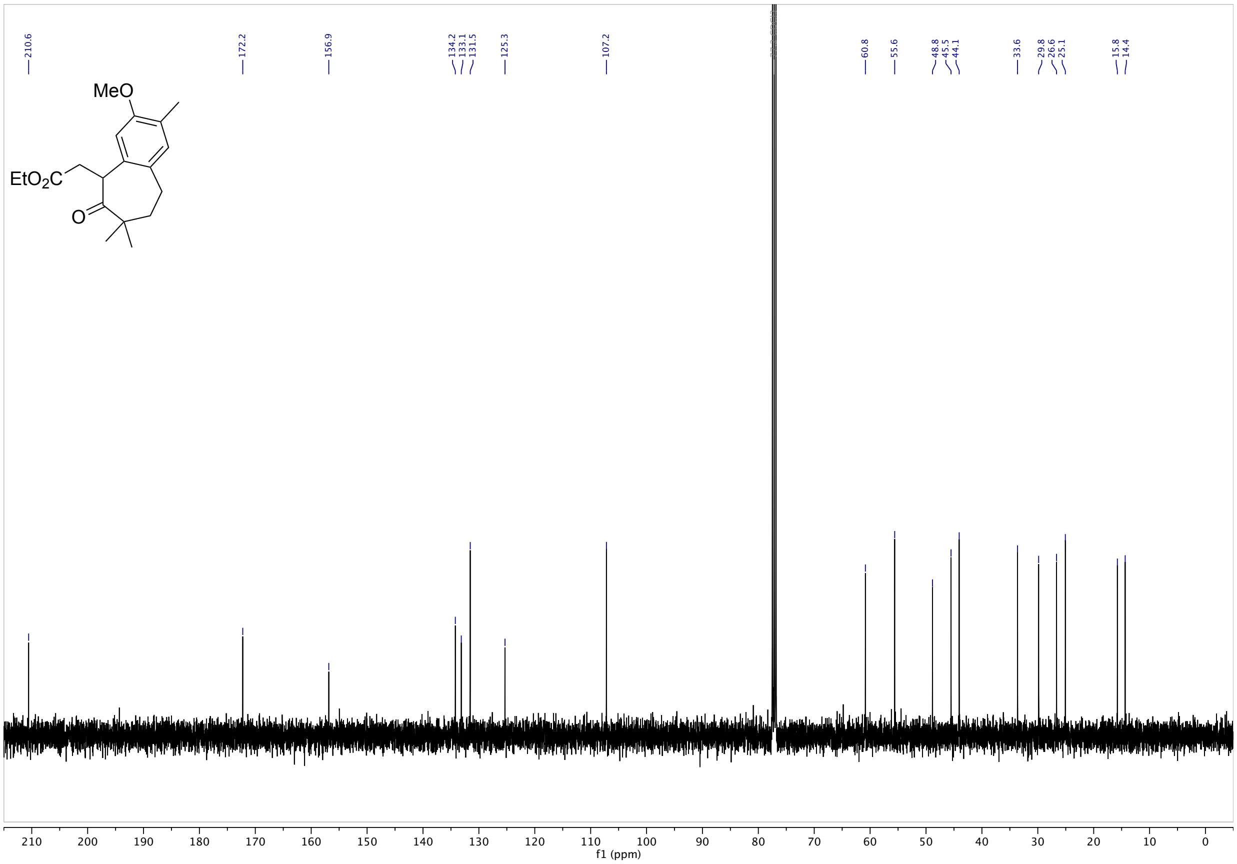

Supplement: Supplementary file 1 — ol3c02796_si_001.pdf [file ol3c02796_si_001.pdf]
